# Supplementary material for: Health-related quality of life and psychosocial impacts of a diagnosis of non-specific genital infection in symptomatic heterosexual men attending UK sexual health clinics: a feasibility study
Source: BMJ Open. 2018 Jun 30;8(6):e018213. doi: 10.1136/bmjopen-2017-018213 (PMC6042625; doi:10.1136/bmjopen-2017-018213)
Supplement: Supplementary file 4 [file bmjopen-2017-018213supp004.pdf]

Supplementary Appendix 4.

Full list of themes for Qualitative Interview (Follow-up 2)

**List of nodes**
antibiotics
attitudes to safe sex and sexual health
consultation
different SHCs
familiarity with SHC
feelings about attending SHC
feelings about being interviewed
feelings about participation in the research
feelings about positive diagnosis
future research
HIV vs. other STIs
interview
invasive tests
NSGI vs. CT
obstacles to participation in research
perception of CT
reaction to NSGI
reasons for participating in research
relationship
reasons for attending SHC
results
risk perception
social support
STI prevention
suggestions for research
survey - was it easy
survey - was it engaging
survey - was it relevant
survey comfort
treatment
waiting for results
waiting time in SHC
