# Supplementary material for: Health-related quality of life and psychosocial impacts of a diagnosis of non-specific genital infection in symptomatic heterosexual men attending UK sexual health clinics: a feasibility study
Source: BMJ Open. 2018 Jun 30;8(6):e018213. doi: 10.1136/bmjopen-2017-018213 (PMC6042625; doi:10.1136/bmjopen-2017-018213)
Supplement: Supplementary file 1 [file bmjopen-2017-018213supp001.pdf]

Supplementary Appendix 1.

Baseline CASI Questionnaire

The following questions are about your background

1. Are you in full time employment? Yes/No/ Prefer not to answer

2. How old were you when you left full-time education?
   - ☐ 16 years or less
   - ☐ 17 or 18 years
   - ☐ 19 years or over
   - ☐ Still in full-time education

3. What is your highest educational achievement?
   - ☐ No qualifications / No formal qualifications
   - ☐ 1-4 GCSEs or equivalent qualifications
   - ☐ 5 GCSEs or equivalent qualifications.
   - ☐ Apprenticeships
   - ☐ 2 or more A-levels or equivalent qualifications.
   - ☐ Bachelors degree or equivalent, and higher qualifications.
   - ☐ Other qualifications including foreign qualifications.

5. Do you have any of the following long-standing conditions? (Tick ALL that apply)
   - ☐ Deafness or severe hearing impairment
   - ☐ Blindness or partially sighted
   - ☐ A long-standing physical condition
   - ☐ A learning disability
   - ☐ A mental health condition
   - ☐ A long-standing illness, such as cancer, HIV, diabetes, chronic heart disease, or epilepsy

6. Does this condition(s) cause you difficulty with any of the following? (Tick ALL that apply)
   - ☐ Everyday activities that people your age can usually do
   - ☐ At work, in education, or training
   - ☐ Access to buildings, streets or vehicles
   - ☐ Reading or writing
   - ☐ People's attitudes to you because of your condition
   - ☐ Communicating, mixing with others, or socialising
   - ☐ Any other activity
   - ☐ No difficulty with any of these

7. To which of these ethnic groups would you say you belong? (Tick ONE only)

a. WHITE
   - ☐ English/Welsh/Scottish/Northern Irish/British
   - ☐ Irish
   - ☐ Gypsy or Irish Traveller

- ☐ Any other White background, write in...

b. MIXED / MULTIPLE ETHNIC GROUPS

- ☐ White and Black Caribbean
- ☐ White and Black African
- ☐ White and Asian
- ☐ Any other Mixed/multiple ethnic background, write in...

c. ASIAN / ASIAN BRITISH

- ☐ Indian
- ☐ Pakistani
- ☐ Bangladeshi
- ☐ Chinese
- ☐ Any other Asian background, write in...

d. BLACK / AFRICAN / CARIBBEAN / BLACK BRITISH

- ☐ African
- ☐ Caribbean
- ☐ Any other Black / African / Caribbean background, write in...

e. OTHER ETHNIC GROUP

- ☐ Arab
- ☐ Any other ethnic group, write in...

The following are questions about recent infections

8. Have you ever been diagnosed with a sexually transmitted infection? If so which?
TICK ALL THAT APPLY

- ☐ Chlamydia
- ☐ Gonorrhoea
- ☐ Genital Herpes
- ☐ Non Specific Genital Infection (NSGI)
- ☐ Genital Warts
- ☐ Syphilis
- ☐ HIV
- ☐ Other (write in)
- ☐ Prefer not to answer

The following questions are about relationships and break-ups

9. At present are you...

- ☐ Married and living with wife
- ☐ Cohabiting but not married
- ☐ Separated, divorced, or, widowed
- ☐ Single (that is never married or never registered in a same-sex civil partnership)
- ☐ Prefer not to answer

9a. How long have you been in this relationship for?

- ☐ 0 – 6 months

- More than 6 months but less than a year
- 1 – 2 years
- 2 or more years
- Prefer not to answer

10. In the last 30 days, have you broken up with someone you considered yourself to be in a relationship with (for example, your girlfriend or wife)?
    - Yes
    - No
    - Prefer not to answer

On the following screens please tap the statement which best describes your own health state today.

11. MOBILITY
    - I have no problems in walking about
    - I have slight problems in walking about
    - I have moderate problems in walking about
    - I have severe problems in walking about
    - I am unable to walk about

12. SELF-CARE
    - I have no problems washing or dressing myself
    - I have slight problems washing or dressing myself
    - I have moderate problems washing or dressing myself
    - I have severe problems washing or dressing myself
    - I am unable to wash or dress myself

13. USUAL ACTIVITIES (e.g. work, Study, housework, family or leisure activities)
    - I have no problems doing my usual activities
    - I have slight problems doing my usual activities
    - I have moderate problems doing my usual activities
    - I have severe problems doing my usual activities
    - I am unable to do my usual activities

14. PAIN / DISCOMFORT
    - I have no pain or discomfort
    - I have slight pain or discomfort
    - I have moderate pain or discomfort
    - I have severe pain or discomfort
    - I have extreme pain or discomfort

15. ANXIETY / DEPRESSION
    - I am not anxious or depressed
    - I am slightly anxious or depressed

- ☐ I am moderately anxious or depressed
- ☐ I am severely anxious or depressed
- ☐ I am extremely anxious or depressed

We would like to know how good or bad your health is TODAY.

This scale is numbered from 0 to 100.
100 means the best health you can imagine. 0 means the worst health you can imagine.

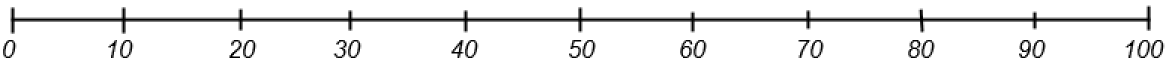

16. Please write the number you think best describes how you feel today. ______________

Below is a list of statements dealing with your general feelings about yourself. Please indicate how strongly you agree or disagree with each statement.

Strongly Agree   Agree   Disagree   Strongly Disagree

17. On the whole, I am satisfied with myself.
18. At times I think I am no good at all.
19. I feel that I have a number of good qualities.
20. I am able to do things as well as most other people.
21. I feel I do not have much to be proud of.
22. I certainly feel useless at times.
23. I feel that I'm a person of worth, at least on an equal plane with others.
24. I wish I could have more respect for myself.
25. All in all, I am inclined to feel that I am a failure.
26. I take a positive attitude toward myself.

Below is a list of statements dealing with your general feelings about your support from people around you. Please indicate how strongly you agree or disagree with each statement.
1 Very Strongly Disagree, 2 Strongly Disagree, 3 Mildly Disagree, 4 Neutral, 5 Mildly Agree, 6 Strongly Agree, 7 Very strongly agree

27. There is a special person who is around when I am in need
28. There is a special person with whom I can share my joys and sorrows
29. My family really tries to help me
30. I get all the emotional help and support I need from my family
31. I have a special person who is a real source of comfort to me
32. My friends really try to help me

33. I can count on my friends when things go wrong
34. I can talk about my problems with my family
35. I have friends with whom I can share my joys and sorrows
36. There is a special person in my life who cares about my feelings
37. My family is willing to help me make decisions.
38. I can talk about my problems with my friends.

We would now like to ask you if you have any feedback for us on how you found completing this questionnaire

39. How did you feel about completing this series of questions? Please write any comments in the space below.

____

____

____

Thank-you for completing this questionnaire
If you would like to speak to someone about the issues raised in this questionnaire please contact us.
