# Supplementary material for: Health-related quality of life and psychosocial impacts of a diagnosis of non-specific genital infection in symptomatic heterosexual men attending UK sexual health clinics: a feasibility study
Source: BMJ Open. 2018 Jun 30;8(6):e018213. doi: 10.1136/bmjopen-2017-018213 (PMC6042625; doi:10.1136/bmjopen-2017-018213)
Supplement: Supplementary file 2 [file bmjopen-2017-018213supp002.pdf]

Supplementary Appendix 2.

Follow-up 1 CASI Questionnaire

On the following screens please tap the statement which best describes your own health state today.

1. MOBILITY
    - ☐ I have no problems in walking about
    - ☐ I have slight problems in walking about
    - ☐ I have moderate problems in walking about
    - ☐ I have severe problems in walking about
    - ☐ I am unable to walk about

2. SELF-CARE
    - ☐ I have no problems washing or dressing myself
    - ☐ I have slight problems washing or dressing myself
    - ☐ I have moderate problems washing or dressing myself
    - ☐ I have severe problems washing or dressing myself
    - ☐ I am unable to wash or dress myself

3. USUAL ACTIVITIES (e.g. work, Study, housework, family or leisure activities)
    - ☐ I have no problems doing my usual activities
    - ☐ I have slight problems doing my usual activities
    - ☐ I have moderate problems doing my usual activities
    - ☐ I have severe problems doing my usual activities
    - ☐ I am unable to do my usual activities

4. PAIN / DISCOMFORT
    - ☐ I have no pain or discomfort
    - ☐ I have slight pain or discomfort
    - ☐ I have moderate pain or discomfort
    - ☐ I have severe pain or discomfort
    - ☐ I have extreme pain or discomfort

5. ANXIETY / DEPRESSION
    - ☐ I am not anxious or depressed
    - ☐ I am slightly anxious or depressed
    - ☐ I am moderately anxious or depressed
    - ☐ I am severely anxious or depressed
    - ☐ I am extremely anxious or depressed

We would like to know how good or bad your health is TODAY.

This scale is numbered from 0 to 100.

100 means the best health you can imagine. 0 means the worst health you can imagine.

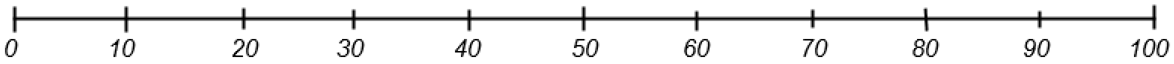

6. Please write the number you think best describes how you feel today. ______________

7. After you had received all your test results from your most recent visit to the GUM clinic were you diagnosed with any of the following infections: Chlamydia, Gonorrhoea or Non-specific urethritis (NSU)? ?
   - ☐ Yes
   - ☐ No
   - ☐ Not sure

The following questions are about your reaction after you were told your test was positive

1= Strongly Disagree 2 = Disagree 3 = Neutral 4 = Agree 5 = Strongly Agree

After I was told that my test was positive:

8. I talked to my friend(s) about it.	1	2	3	4	5
9. I do not think it was a big deal.	1	2	3	4	5
10. I was/am scared to tell my partner(s).	1	2	3	4	5
11. It does not change the way I feel about myself.	1	2	3	4	5
12. My partner thinks that I cheated on him or her.	1	2	3	4	5
13. I am not worried since it is curable.	1	2	3	4	5
14. I feel like I did not take care of myself.	1	2	3	4	5
15. I feel betrayed by my partner.	1	2	3	4	5
16. I worry that it will not go away.	1	2	3	4	5
17. I feel guilty that I could have given someone my infection	1	2	3	4	5
18. It has not changed my relationship with my partner.	1	2	3	4	5
19. I am not very proud of my actions.	1	2	3	4	5
20. I do not blame myself.	1	2	3	4	5
21. I think that my partner cheated on me.	1	2	3	4	5
22. I worry that I might not be able to have children in the future.	1	2	3	4	5
23. I do not really care that much.	1	2	3	4	5
24. I am angry.	1	2	3	4	5
25. I feel really bad about myself.	1	2	3	4	5
26. It does not change how my friends feel about me.	1	2	3	4	5
27. It will be hard for me to trust future partners.	1	2	3	4	5
28. I am not worried about getting it again.	1	2	3	4	5
29. I feel dirty.	1	2	3	4	5
30. I broke up with my partner.	1	2	3	4	5

31. I worry about what other sexually transmitted infections I could have been exposed to.	1	2	3	4	5
32. I feel really embarrassed.	1	2	3	4	5

The next questions are about how you feel generally about the infection you were diagnosed with

33. I think about the infection I was diagnosed with a lot.
1= Strongly Disagree 2 = Disagree 3 = Neutral 4 = Agree 5 = Strongly Agree

34. I am concerned about the infection I was diagnosed with.
1= Strongly Disagree 2 = Disagree 3 = Neutral 4 = Agree 5 = Strongly Agree

Below is a list of statements dealing with your general feelings about yourself. Please indicate how strongly you agree or disagree with each statement.

Strongly Agree Agree Disagree Strongly Disagree

35. On the whole, I am satisfied with myself.
36. At times I think I am no good at all.
37. I feel that I have a number of good qualities.
38. I am able to do things as well as most other people.
39. I feel I do not have much to be proud of.
40. I certainly feel useless at times.
41. I feel that I'm a person of worth, at least on an equal plane with others.
42. I wish I could have more respect for myself.
43. All in all, I am inclined to feel that I am a failure.
44. I take a positive attitude toward myself.

The following questions are about relationships and break-ups

45. At present are you...
- ☐ married and living with wife
- ☐ cohabiting but not married
- ☐ separated, divorced, or, widowed
- ☐ single (that is never married)
- ☐ prefer not to answer

45a. How long have you been in this relationship for?
- ☐ 0 – 6 months
- ☐ More than 6 months but less than a year
- ☐ 1 – 2 years
- ☐ 2 or more years
- ☐ Prefer not to answer

46. In the last 30 days, have you broken up with someone you considered yourself to be in a relationship with (for example, your girlfriend or wife)?

- ☐ Yes
- ☐ No
- ☐ Prefer not to answer

The next questions are about your most recent visit to the GUM Clinic

47. Have you ever visited this GUM Clinic before for the same condition?

- ☐ Yes
- ☐ No
- ☐ Prefer not to answer

48. Did a member of staff explain the results of the tests in a way you could understand?

- ☐ Yes definitely
- ☐ Yes, to some extent
- ☐ No
- ☐ Not sure, can't remember
- ☐ I was told I would get the results at a later date
- ☐ I was never told the results of the tests

49. While you were in the GUM Clinic how much information about your condition or treatment was given to you?

- ☐ Not enough
- ☐ Right amount
- ☐ Too much
- ☐ I was not given any information about my treatment or condition

50. Was the main reason you went to the GUM Clinic dealt with to your satisfaction?

- ☐ Yes, completely
- ☐ Yes, to some extent
- ☐ No

The next questions are about the symptoms you had when attending the clinic (the time you were first asked to join this study)

51. Please choose the option that best fits how you feel

- ☐ My symptoms have completely gone away
- ☐ My symptoms are better but not completely
- ☐ My symptoms are the same
- ☐ My symptoms are worse than they were before
- ☐ Don’t know/not sure
- ☐ Prefer not to answer

We would now like to ask you if you have any feedback for us on how you found completing this questionnaire

52. How did you feel about completing this series of questions? Please write any comments in the space below.

____
____
____

Thank-you for completing this questionnaire
If you would like to speak to someone about the issues raised in this questionnaire please contact us.
