# Supplementary material for: Health-related quality of life and psychosocial impacts of a diagnosis of non-specific genital infection in symptomatic heterosexual men attending UK sexual health clinics: a feasibility study
Source: BMJ Open. 2018 Jun 30;8(6):e018213. doi: 10.1136/bmjopen-2017-018213 (PMC6042625; doi:10.1136/bmjopen-2017-018213)
Supplement: Supplementary file 3 [file bmjopen-2017-018213supp003.pdf]

Supplementary Appendix 3.

Individual in-depth interview Topic Guide

Topics

Life history / framing (~5 minutes)

Please tell me a bit about yourself.

What is the most important thing you can tell me about yourself?

Probes:

Hobbies/interests

Goals

Tell me about your family.

Who do you include in your family? Partner/spouse? Father/mother? Grandparents? Children?

What sorts of things do you do with your day? / Describe a typical day in your life.

Probes:

Are you in school/university?

What do you do for money?

Clinical experience (~25 minutes)

Please describe how you were feeling while you were at the clinic on the day you were asked to take part in this study.

Probes:

Describe the reasons why you came to clinic

(If symptoms) What affect (if any) did your symptoms have on your day-to-day life?

How did it go/ how did you feel about the visit?

How long were you waiting to be seen? Was this expected /unexpected?

Which infections were you tested for?

What did the doctor tell you they thought you had? (i.e. diagnosis, if any)

Now let's discuss your diagnosis.

What thoughts were going through your mind when you were told you had NSU/Ng/possibly an STI (use patients' own description/language for their diagnosis)?

Probes:

How did you feel about your diagnosis?

Have you been diagnosed with NSU/Ng before? Any other infections? If yes, please describe any differences in your feelings from the first time you were diagnosed compared with this recent diagnosis.

Did you tell anyone (partner/family members/friends) your diagnosis? If yes, who and why? What was their reaction?

Research experience (~15 minutes)

We want to make sure that the research we conduct is done in the best way possible for patients. This next section of our interview is based on your experience with this research study so far. Please remember that your answers to these questions will not affect your clinical care, your legal rights, or future invitations to participate in research. It is important that you speak freely so that we can improve future research in this area.

How did you feel about your participation in the research?

Probes:

Would you consider involving yourself in this kind of research in the future? Why/why not?

Were you able to complete the surveys?

Did you have any problem accessing the CASI? (Logistical/technical problems) If so, what were the problems you faced?

Did you feel comfortable accessing the CASI (inside/outside of clinic)? Why/why not?

How did you feel about the questionnaires?

Discuss each questionnaire individually in turn: demographic and clinic experience questions; EQ5D-5L; social support scale; self-esteem scale:

Did you feel these questions were relevant?

Did you feel comfortable answering these questions? Why/why not?

Discuss any suggestions you may have for improvement of these questionnaires

Anything else?

Thanks

Reimbursement
