# Supplementary material for: Health-related quality of life and psychosocial impacts of a diagnosis of non-specific genital infection in symptomatic heterosexual men attending UK sexual health clinics: a feasibility study
Source: BMJ Open. 2018 Jun 30;8(6):e018213. doi: 10.1136/bmjopen-2017-018213 (PMC6042625; doi:10.1136/bmjopen-2017-018213)
Supplement: Author's manuscript [file bmjopen-2017-018213.draft_revisions.pdf]

BMJ Open

BMJ Open is committed to open peer review. As part of this commitment we make the peer review history of every article we publish publicly available.

When an article is published we post the peer reviewers' comments and the authors' responses online. We also post the versions of the paper that were used during peer review. These are the versions that the peer review comments apply to.

The versions of the paper that follow are the versions that were submitted during the peer review process. They are not the versions of record or the final published versions. They should not be cited or distributed as the published version of this manuscript.

BMJ Open is an open access journal and the full, final, typeset and author-corrected version of record of the manuscript is available on our site with no access controls, subscription charges or pay-per-view fees (http://bmjopen.bmj.com).

If you have any questions on BMJ Open's open peer review process please email info.bmjopen@bmj.com

Health related quality of life and psychosocial impacts of a diagnosis of non-specific genital infection in symptomatic men attending UK Sexual Health clinics: a feasibility study.

Journal:	*BMJ Open*
Manuscript ID	bmjopen-2017-018213
Article Type:	Research
Date Submitted by the Author:	17-Jul-2017

Complete List of Authors:	Hill-Tout, Rachel; St George's NHS Foundation Trust, Genitourinary Medicine & HIV<br>Harding- Esch, Emma; Public Health England, HIV & STI; St George's University of London, Applied Diagnostic Research and Evaluation Unit, Institute for Infection & Immunity<br>Pacho, Agata; St George's University of London, Applied Diagnostic Research and Evaluation Unit, Institute for Infection & Immunity<br>Furegato, Martina; Public Health England, HIV & STI<br>Fuller, Sebastian; St Georges University of London, Applied Diagnostic Research and Evaluation Unit, Institute for Infection & Immunity<br>Sadiq, S. Tariq; St George's University of London, Applied Diagnostic Research and Evaluation Unit, Institute for Infection and Immunity Research Institute

**Primary Subject Heading**:	Sexual health
Secondary Subject Heading:	Patient-centred medicine, Qualitative research, Sociology, Sexual health
Keywords:	GENITOURINARY MEDICINE, QUALITATIVE RESEARCH, Genitourinary medicine < INTERNAL MEDICINE

For peer review only - http://bmjopen.bmj.com/site/about/guidelines.xhtml

Health related quality of life and psychosocial impacts of a diagnosis of non-specific genital infection in symptomatic men attending UK Sexual Health clinics: a feasibility study.

Rachel Hill-Tout[1], Emma M. Harding-Esch[2, 3], Agata Pacho[2], Martina Furegato[3], Sebastian S. Fuller*[†2], S Tariq Sadiq[†1, 2]

* Corresponding author

† Joint senior authors

1. St Georges University Hospitals NHS Foundation Trust
   Blackshaw Rd, London SW17 0QT UK

2. Applied Diagnostic Research and Evaluation Unit
   Institute for Infection & Immunity
   St George's, University of London
   London, SW17 0RE, UK

3. Public Health England
   61 Colindale Avenue
   London, NW9 5EQ UK

Rachel Hill-Tout
Specialist Registrar HIV/GUM
orcid.org/0000-0003-1892-8598
r.hill-tout@nhs.net

Emma M. Harding-Esch
ADREU Epidemiology Lead & Programme Manager
orcid.org/0000-0002-1432-8109

Agata Pacho
ADREU Research Assistant
orcid.org/0000-0002-0653-5095

Martina Furegato
Statistician - HIV & STI department

Sebastian S. Fuller
ADREU Social Science Lead
orcid.org/0000-0002-9284-6630

S Tariq Sadiq
Reader in HIV and Sexual Health Medicine, Consultant Physician / ADREU Lead
orcid.org/0000-0002-0608-381

ABSTRACT

Introduction:

Non-specific genital infection (NSGI; non-*Chlamydia trachomatis* (CT), non-*Neisseria gonorrhoeae* (NG) associated urethritis) is a common diagnosis in symptomatic heterosexual men attending UK Sexual Health Clinics (SHCs) but little is known about the psychosocial impact of this diagnosis.

For peer review only - http://bmjopen.bmj.com/site/about/guidelines.xhtml

Methods:

We conducted a feasibility study to measure psychosocial impact of an NSGI diagnosis among symptomatic heterosexual men attending SHCs compared to the impact of a diagnosis of CT, NG or no abnormalities detected (NAD). Participants completed a Computer Assisted Self Interview (CASI) including validated health-related quality-of-life (HRQoL) and self-esteem measures before diagnostic testing and two-weeks after receiving test results, and a qualitative interview. We compared scores between diagnostic groups using paired t-tests, qualitative data were analysed thematically, and feasibility was assessed by process analysis.

Results:

60 men completed the baseline CASI (75% response rate). 46 (76.6%) were eligible for follow-up; of these, 11 (23.9%) completed the follow-up CASI, and 3 (27.3%) completed the qualitative interview. 81.7% of participants left CASI feedback at baseline: 73.5% reported the questionnaire as 'fine' or 'very good'. Qualitative interview participants reported the study was acceptable. All patients completing FU-1 showed measurable decreases from baseline to FU-1 in self-esteem (CT p=0.009; NSGI p=0.008; NAD p=0.057). Measurable decreases in HRQoL from baseline to FU-1 were found among participants with CT (p=0.002) or NSGI (p=0.001). We did not find differences in baseline sociodemographic factors between participants who completed FU-1 and those that did not.

Conclusions:

Although most participants indicated study acceptability at baseline, and we employed measures to increase retention (CASI questionnaires, reminder messages, a focus on men's health), attrition was very high. We found that heterosexual men attending SHCs with symptoms of urethritis may experience negative psychosocial impacts regardless of their diagnosis which warrants further investigation. We recommend exploring novel strategies to decrease attrition in future studies.

Article Summary

Strengths and Limitations of this study

- This is the first data to our knowledge investigating the psychosocial impact of non-specific genital infection, a very common diagnosis in men attending sexual health clinics.
- This study used both validated scales and qualitative interviews to assess psychosocial impact.
- We used computer assisted self-interviewing to collect data on psychosocial impact as this has been shown to improve data quality in sensitive topics.
- The study was small and was conducted in 2 London sexual health clinics which may limit the generalisability of our results.
- There was high loss to follow-up after baseline interviews which may have biased our results.

For peer review only - http://bmjopen.bmj.com/site/about/guidelines.xhtml

INTRODUCTION

Non-specific Genital Infection (NSGI) or, non-*Chlamydia trachomatis* (CT), non-*Neisseria gonorrhoeae* (NG) associated urethritis (NGU), is the third most frequently diagnosed condition in heterosexual men attending sexual health clinics (SHCs) in the United Kingdom (UK), with 30,381 cases/year compared to CT (42,483/year) and first presentation of genital warts (32,656/year) [1]. NSGI is a diagnosis of exclusion; men with urethral symptoms are screened with urethral microscopy in SHCs, if this demonstrates urethritis with no gonococci, a provisional diagnosis of NGU is made [2]. Urinary Nucleic Acid Amplification Tests (NAATs) are sent for NG and CT, if these are both negative, the diagnosis is recorded as NSGI.

Although a common condition, NSGI remains poorly understood. NSGI can be caused by a variety of sexually transmitted infections (STIs), including *Mycoplasma genitalium* (MG) and *Trichomonas vaginalis* (TV), which are not routinely tested for in SHCs, and many cases have an uncertain aetiology [2]. Microscopy, on which the diagnosis rests, has poor specificity for known causes of urethritis [3] and is subject to considerable observer variation [4].

National guidelines recommend that men diagnosed with NGU are informed they may have an STI, given same day empirical antibiotics, advised their partners should attend for STI testing and empirical treatment, and to abstain from sex until treatment is completed [2]. Men receive CT/NG NAAT results usually within 8 days [5]. Following first-line antibiotics for NSGI, 10%–20% of men return because of persistent symptoms [6,7].

Men who undergo STI testing and are diagnosed with CT report negative impacts on their psychosocial wellbeing [8,9], however, to our knowledge, there are no published data on the psychosocial impact of NSGI diagnosis among men.

Men are reportedly poor users of health services, largely due to culturally dominant ideals of masculinity that equate illness with weakness, which may impede care-seeking. [10–13] In sexual healthcare, there has traditionally been a focus on female reproductive health [14], and this may further discourage male attendance [14,15] However, men are willing to respond openly to questions about their sexual health, particularly when enquiries are focused specifically on men's needs. [14,16]

Because of a lack of models to measure the psychosocial impact of an NSGI diagnosis among men, and the potential difficulty in recruiting and retaining this group in sexual health studies, we designed a small-scale longitudinal mixed-method feasibility study to inform on our research methods and better understand the psychosocial and health related quality of life (HRQoL) status of symptomatic heterosexual men before and after receiving a diagnosis of NSGI, CT, NG or nothing abnormal detected (NAD).

For peer review only - http://bmjopen.bmj.com/site/about/guidelines.xhtml

METHODS

Study Design

We conducted a longitudinal study in symptomatic heterosexual men using patient completed questionnaires assessing HRQoL and psychosocial status before and after receiving STI test results, and individual qualitative interviews to evaluate experiences at the SHC and acceptability of study methods.

Eligibility criteria were: heterosexual men presenting with urethral symptoms (dysuria, discharge or urethral discomfort), aged ≥16 years at either of two participating London SHCs between September - October 2016. We excluded men who were asymptomatic, not available for follow-up, and who were unable to understand English.

Following informed consent, participants were invited to complete a baseline computer assisted self-interview (CASI) assessing psychosocial and HRQoL status in clinic prior to consultation, a follow-up CASI after receipt of all test results 1-2 weeks after baseline (FU-1) and a qualitative interview 2-4 weeks after baseline (FU-2).

The participant pathway is detailed in Figure 1. After completion of the baseline CASI, any participants diagnosed with an STI other than CT, NG or NSGI, or who did not have both urethral microscopy and urinary NAATs, were withdrawn from the study. Once participants were notified of their NAAT CT/NG results by the SHC (phone call/ text message), the research team texted participants an electronic link to the FU-1 CASI.

CASIs were optimised for web-enabled devices including smartphones. We offered participants the choice to complete CASIs using their own device or a study tablet-device and use of a private room in clinic. Study staff sent ≤2 text message reminders for each follow-up. Participants who did not complete FU-1 were considered ineligible for FU-2. Participants received gift voucher reimbursements: £5 (baseline), £10 (FU-1), £20 (FU-2).

We categorised participants into four diagnostic groups based on microscopy and NAAT results: NSGI: NGU diagnosis on urethral microscopy and CT/NG NAATs negative; NG: NG on urethral microscopy and/or positive NAAT; CT: positive NAAT; NAD: no urethritis on microscopy and CT/NG NAATs negative.

Questionnaire design

Baseline and follow-up CASIs included validated scales measuring psychosocial status and HRQoL and additional questions. The EQ-5D-5L scale [17] as a measure of HRQoL and the Rosenberg Self-Esteem scale [18] as a measure of potential stigmatising effects of screening for STIs, were used in both baseline and FU-1 CASIs.

In addition to the above, the baseline CASI included the Multidimensional Scale of Social Support [19] to assess coping resources and questions on sociodemographic and current relationship status, and the FU-1 CASI included a question on current relationship status and a panel of questions previously developed to assess impact of CT diagnosis in women, [20] which we modified for use in men by removing a pregnancy-related

For peer review only - http://bmjopen.bmj.com/site/about/guidelines.xhtml

question. Participants were asked to indicate acceptability of the CASIs (free-text response) at baseline and FU-1. (Baseline and FU-1 questionnaires and FU-2 topic guide: Appendix 1, Supplementary Information.)

Sample size

As this was a feasibility study with no precedent, no formal sample size could be calculated. We planned to approach 100 patients at baseline as a convenience target, and assuming approximately 50% refusal, we expected 50-60 patients to be enrolled and estimated ~50% attrition at each follow-up.

Outcome measures

Primary outcome: Qualitative and quantitative process data on recruitment and retention rates at baseline and follow-up to inform on feasibility. Evaluation of potential relevance of questions validated in other populations for men diagnosed with NSGI.

Secondary outcomes:

Percentage change from baseline to FU-1 in mean EQ-5D-5L and Rosenberg Self-Esteem Scale in men diagnosed with CT, NG, NSGI or NAD.

Data analysis

Questionnaire data:

Scores for psychosocial and HRQoL scales were calculated according to published methods [18][17][19]. For all eligible men, mean and 95% Confidence Intervals of baseline scores for EQ-5D-5L, Rosenberg Self-Esteem and Multidimensional Social Support Scales were compared in men diagnosed with CT, NG, NSGI and NAD, using paired t-tests. To test for non-response bias we compared sociodemographic data of those who completed both baseline and follow-up to those who were lost to follow-up (LTFU) using Pearson's Chi^2 tests and Kruskal-Wallis tests. Paired t-tests were used to assess differences in mean score for EQ-5D-5L and Rosenberg Self-Esteem Scales between baseline and follow-up in men diagnosed with CT, NG, NSGI and NAD. Statistical significance was set at $p<0.05$. Statistical analysis was carried out using STATA version 13.1 (StataCorp, 2015).

Qualitative data: Audio recordings were transcribed and transcripts checked for accuracy against the recording. Cleaned transcripts were then imported into NVivo (Version 10, 2012) for analysis. Two researchers assigned themes to text independently, with discrepancies resolved by consensus before final themes reported. All names of participants presented here are pseudonyms.

RESULTS

We approached 80 patients, of whom 60 (75%) enrolled and completed the baseline CASI. 46 (76.6%) were eligible for follow-up; of these, 11 (23.9%) completed the FU-1 CASI, and 3 of these (27.3%) completed FU-2 (Figure 2.)

For peer review only - http://bmjopen.bmj.com/site/about/guidelines.xhtml

Final diagnoses among all eligible participants (n=46) were: CT, 7 (15.2%); NG, 2 (4.3%); NSGI, 23 (50%); NAD, 14 (30.4%) and among participants who completed FU-1 (n=11) were CT, 4 (36.4%); NSGI, 4 (36.4%); NAD, 3 (27.3%). None of the participants diagnosed with NG completed FU-1. Two participants with NSGI and one with NAD completed FU-2. Clinical records indicated that all participants were notified of their CT/NG NAAT diagnosis within 7 days of completing baseline.

Participants' sociodemographic factors at baseline are presented in Table 1. Most were between 25-35 years old (60.9%) and over half reported their ethnicity as non-White (52.2%). 80.4% indicated they were not in a relationship and many had previously been diagnosed with STIs (30.4% NSGI, 28.3% CT, 13.0% genital herpes or warts and 8.7% NG).

Pearson's Chi^2 tests and Kruskal-Wallis test results for non-response bias were not statistically significant so we assumed data were missing at random. We compared participants who completed FU-1 to those LTFU by employment, age, education, ethnicity, and current relationship status; none of these comparisons showed significant differences.

Process evaluation

Participants completed the baseline CASI in about 5 minutes. All participants used the study tablet-device and completed the survey in a private room in the clinic. CASI feedback suggested good acceptability: of the total 60 who completed the Baseline CASI, 81.7% left feedback, of whom 73.5% reported that the questionnaire was "fine" or "very good" in a free-text box at the end of the questionnaire.

Participants suggested improvements to the study during qualitative interviews: to use the research as a tool for health promotion and to make CASI questions more clearly relevant to sexual health. Insight into LTFU was provided by one participant, who said he might not have continued with the study if he had received a positive STI diagnosis: "*I reckon if my tests had not come back the way I wanted them to, I probably wouldn't have done this last [follow-up]. … I might've still said, 'Yes, let's do it.' I don't know but I… Just sitting here, thinking about it now, I probably wouldn't complete the rest of it."* (Lucas, NAD diagnosis).

CASI analysis

Distributions of participants' psychosocial scores at baseline by final diagnosis are shown in Table 2. At baseline participants ultimately diagnosed with NG had significantly lower (p≤0.050) mean Multidimensional Scale of Social Support scores (indicating lower social support), compared to men ultimately diagnosed with NSGI or NAD as well as lower scores as compared to those diagnosed with CT (p=0.059). Participants with NG also had lower mean Rosenberg Self-esteem scores (indicating lower self-esteem) at baseline as compared to those with NSGI (p=0.038). We did not find any other baseline differences between diagnosis groups.

For peer review only - http://bmjopen.bmj.com/site/about/guidelines.xhtml

We found men with NSGI and CT showed a significant decrease in mean EQ-5D-5L score between baseline and FU-1(-39%, p=0.001 and -44%, p=0.002) indicating decreased HRQoL. There were also decreases in self-esteem from baseline to FU-1 seen in all participants, as measured by mean Rosenberg Self-Esteem scores (NSGI=-25%, p=0.008; CT=-29%, p=0.009; NAD=-21%, p=0.057).

Participants who reported that they had been diagnosed with an infection (n=5, 4 with a final diagnosis of CT and 1 with NSGI) completed a panel of questions in the FU-1 CASI to assess their feelings about their diagnosis. All participants reported that they were 'not worried as it is curable' and did not think the diagnosis would change how their friends thought about them, but only one participant (diagnosed with CT) reported he had talked to his friends about his diagnosis. All participants reported concern that they might get the infection again, and the majority of those with a CT diagnosis (75%) reported concern about exposure to other STIs, feeling “dirty” and embarrassed, being scared to tell their partners about their diagnosis and worried that their partners thought they had been unfaithful.

Qualitative analysis

In interviews, participants compared different STIs as a way to contextualise their level of concern about their own diagnosis. For one participant, his main concern was the possibility of being diagnosed with HIV: *“Look, to be honest with you, the key one everyone really wants to know about is HIV … all the other ones are treatable quite easily…”* (Lucas, NAD diagnosis). There were several points within the interviews where participants expressed concern at being diagnosed with an infection they did not know about, indicating that non-specific diagnoses may cause negative impact: “*…to be honest, I’m a little bit worried about this because there is no name of the new bacteria, so we don’t know if it’s dangerous or not...”* (Liam, NSGI diagnosis).

Among the two interview participants diagnosed with NSGI, one described anxiety following his presumptive CT diagnosis in clinic. He was in a long-term monogamous relationship and so was not expecting an STI diagnosis: *“My heart’s racing. I’m thinking, ‘but how, I can’t.’ … I just had a lot of thoughts in my head. … And I told [the doctor], ‘I can’t. I don’t know how I would have this.’ He’s saying, ‘Well, whatever you have, it’s sexually transmitted.’ I said, ‘But it can’t be.’ So of course I start getting things in my head.”* (Jacob, NSGI diagnosis). (Full list of themes: Appendix 2, Supplementary Information)

DISCUSSION

In this small feasibility study we detected decreases in mean HRQoL and self-esteem scores in participants with a final diagnosis of CT or NSGI from the time of clinical presentation to approximately one week after receiving NAAT results. Although participants diagnosed with CT and NSGI showed greater negative impact in this time period, interestingly, participants who ultimately received a NAD diagnosis also experienced decreased self-esteem scores. Studies using qualitative methods and non-validated questionnaires have shown men diagnosed with CT experience negative psychosocial impacts [7, 8]. However, this is the first time that a negative impact of diagnosis of NSGI has been demonstrated to our knowledge. In interviews, participants with NSGI reported concerns regarding the uncertainty of the diagnosis, and fear of the impact of a possible

For peer review only - http://bmjopen.bmj.com/site/about/guidelines.xhtml

STI diagnosis on their relationship. These findings suggest receipt of an STI or presumptive STI diagnosis in this population may have complex psychosocial impacts. These impacts may be multi-factorial and anxiety seems to be highly contextual and affected by relationship status and knowledge about STIs.

We embedded several mechanisms in our study to improve participant acceptability and retention. Privacy when completing sensitive questions has been shown to improve participant acceptability for revealing potentially stigmatising information [21]; we used a web-based CASI and the option for participants to self-complete the questionnaire in a private room in the clinic. Potential research participants, and men specifically, are more likely to participate in research that represents their concerns [14,16,22]; eligibility for this study was restricted to those patients who were likely to be diagnosed with an STI and focused on the impact of those diagnoses. In addition, reimbursements have been shown to play a part in increasing enrolment and retention in research [22,23] ; we reimbursed participants in increasing amounts to reflect the time and effort we estimated were necessary to complete the research.

Despite these mechanisms, and positive participant feedback on the baseline CASI and in our qualitative interviews, we experienced high LTFU. High LTFU resulted in low numbers of respondents within diagnostic categories, which meant we were unable to adjust our analysis for common confounders, such as education and socioeconomic status, known to be associated with gonorrhoea [24,25] . This may be a source of bias in our results. We explored potential correlates and causes for LTFU within our dataset. Men's socioeconomic status and age has been seen to affect both their knowledge of and interaction with health services[15]. However, we did not find significant differences in these factors between those LTFU and those who completed FU-1. One participant indicated in qualitative interview that if he had received a positive STI diagnosis it was unlikely that he would have agreed to continue with the research. This may give insight into why no patients receiving a positive CT or NG diagnosis agreed to FU-2.

We had planned to further explore study acceptability by inviting participants who failed to complete FU-1 to qualitative interviews; however, we followed Research Ethics Committee recommendations to consider that these participants had passively withdrawn from the study and refrained from inviting them to FU-2. However, qualitative and quantitative methods of data collection are viewed differently by respondents [26,27]. One participant described the difference between his experiences completing the survey and participating in an interview:

"…when I'm sitting here with you, obviously it's just me and you and you've got my hundred percent full attention, haven't you? But then when I'm sitting at my desk at work, trying to complete a survey and there's other things going on and people asking me questions and 'can you do this, can do you that'; it's different." (Lucas, NAD diagnosis)

For peer review only - http://bmjopen.bmj.com/site/about/guidelines.xhtml

Although we cannot know if participants who did not complete FU-1 would have participated in a qualitative interview if they had been invited, one participant left feedback on the baseline CASI indicating he would have preferred to elaborate on his answers, and several participants informally conveyed this to the researcher at baseline. Research has shown that one unique benefit of qualitative interviews is that it provides participants with an opportunity to expand or contextualise issues [26,27] . In addition, the insights we gained from the limited qualitative interviews we conducted indicate the potential usefulness of this method to understand nuances of acceptability that may lead to reduced LTFU. We therefore recommend that feasibility studies include qualitative components as a means to allow participants, including those who fail to complete survey questionnaires, to provide important feedback on study methods.

Although the Rosenberg Self Esteem scale and the EQ-5D-5L scales we used in this study have been validated in men and women, the panel of questions used to assess men's' feelings about a diagnosis of CT or NSGI in the FU-1 CASI has not been validated in men, or for non-CT STI diagnoses. In addition, we assessed psychosocial and HRQoL impact at a single time-point, which does not measure durability of the observed decrease in self-esteem in this population, and data suggest impact of STI diagnoses may decrease over time [28].

In conclusion, our study shows that heterosexual men with urethral symptoms may experience negative psychosocial effects following SHC attendance. Given that incidence of NSGI is high [16] and up to 20% of men affected have recurrent symptoms [5, 6], a significant number of men may be adversely affected. Future research in this area is needed, firstly to address the high level of LTFU, and then to test the generalisability of our findings as to whether there are significant psychosocial harms of giving presumptive and non-specific diagnoses.

TABLES AND FIGURES

Figure 1. Patient Pathway (see uploads)
Figure 2. Enrolment and Follow-up Chart (see uploads)
Table 1 (below) Sociodemographic characteristics of enrolled participants at Baseline by final diagnosis
Table 2. (a, b) Distribution of psychosocial scores by final diagnosis (below)

Supplementary Information (see uploads)
Appendix 1. Baseline CASI Questionnaire; FU-1 CASI Questionnaire; Individual in-depth interview Topic Guide
Appendix 2. Full list of themes (Qualitative Interview (FU-2))

For peer review only

For peer review only - http://bmjopen.bmj.com/site/about/guidelines.xhtml

Table 1. Sociodemographic characteristics of enrolled participants at Baseline by final diagnosis

	CT (n)	NG (n)	NSGI (n)	NAD (n)	Total	CT (%)	NG (%)	NSGI (%)	NAD (%)
**Age**									
<24	5	1	5	3	14	71.43	50.00	21.74	21.43
25-35	2	1	17	8	28	28.57	50.00	73.91	57.14
>35	0	0	1	3	4	0.00	0.00	4.35	21.43
**Ethnicity**									
White	5	0	10	6	21	71.43	0.00	43.48	42.86
Asian	0	1	2	2	5	0.00	50.00	8.70	14.29
Black	1	1	10	2	14	14.29	50.00	43.48	14.29
Other	1	0	1	3	5	14.29	0.00	4.35	21.43
Prefer not to say	0	0	0	1	1	0.00	0.00	0.00	7.14
**Age at leaving full-time education**									
16 years or less	0	0	1	3	4	0.00	0.00	4.35	21.43
17 or 18 years	0	0	6	0	6	0.00	0.00	26.09	0.00
19 years or over	4	0	14	10	28	57.14	0.00	60.87	71.43
Still in full-time education	3	2	2	1	8	42.86	100.00	8.70	7.14
**Highest educational achievement**									
No qualifications/No formal qualifications	1	0	1	0	2	14.29	0.00	4.35	0.00
1-5 GCSEs	0	0	3	2	5	0.00	0.00	13.04	14.29
2 or more A-levels or equivalent	1	1	4	2	8	14.29	50.00	17.39	14.29
Bachelor's degree or equivalent and higher	5	1	13	8	27	71.43	50.00	56.52	57.14
Other	0	0	2	2	4	0.00	0.00	8.70	14.29
**Do you have any long-standing condition?**									
Yes	1	0	1	2	4	14.29	0.00	4.35	14.29
No	6	2	22	12	42	85.71	100.00	95.65	85.71
**Previously diagnosed with CT?**									
Yes	4	1	7	1	13	57.14	50.00	30.43	7.14
No	3	1	16	13	33	42.86	50.00	69.57	92.86
**Previously diagnosed with NG?**									
Yes	0	2	2	0	4	0.00	100.00	8.70	0.00

For peer review only - http://bmjopen.bmj.com/site/about/guidelines.xhtml

No	7	0	21	14	42	100.00	0.00	91.30	100.00
**Previously diagnosed with NGU?**									
Yes	2	0	9	3	14	28.57	0.00	39.13	21.43
No	5	2	14	11	32	71.43	100.00	60.87	78.57
**Previously diagnosed with herpes/warts?**									
Yes	3	0	1	2	6	42.86	0.00	4.35	14.29
No	4	2	22	12	40	57.14	100.00	95.65	85.71
**Relationship status**									
Married and living with wife	0	0	1	2	3	0.00	0.00	4.35	14.29
Cohabiting but not married	1	0	2	2	5	14.29	0.00	8.70	14.29
Separated, divorced, or widowed	0	0	1	0	1	0.00	0.00	4.35	0.00
Single (that is never married)	6	2	19	9	36	85.71	100.00	82.61	64.29
Prefer not to say	0	0	0	1	1	0.00	0.00	0.00	7.14
**Break up in the last 30 days**									
Yes	1	0	3	5	9	14.29	0.00	13.04	35.71
No	6	2	20	9	37	85.71	100.00	86.96	64.29

For peer review only

Table 2 Distribution of psychosocial scores by final diagnosis

Tab 2.a Distribution of psychosocial scores by final diagnosis within the baseline group and results from t-test

		Mean	95% CI	p-value NSGI vs. CT	p-value NSGI vs. NAD	p-value CT vs. NAD	p-value NG vs. CT	p-value NG vs. NSGI	p-value NG vs. NAD
**EQ-ED-5L**	**NG**	2.2	1.4-3.0	0.255	0.227	0.999	0.932	0.430	0.940
	**NSGI**	2.0	1.9-2.1						
	**CT**	2.2	1.9-2.5						
	**NAD**	2.2	1.9-2.4						
**Rosenberg Self Esteem Scale**	**NG**	2.1	1.2-3.0	0.819	0.582	0.835	**0.068**	**0.038**	0.122
	**NSGI**	3.2	3.0-3.4						
	**CT**	3.3	2.9-3.7						
	**NAD**	3.3	3.0-3.6						
**Multidimensional scale of social support**	**NG**	2.5	2.0-3.0	0.583	0.610	0.918	**0.059**	**0.016**	**0.040**
	**NSGI**	3.1	2.9-3.2						
	**CT**	3.2	2.8-3.6						
	**NAD**	3.2	2.9-3.4						

Tab 2.b Distribution of psychosocial scores by final diagnosis within the FU group and within the Baseline group restricted to those with a FU record comparing Baseline and FU scores

		Baseline*		FU-1			
		Mean	95% CI	Mean	95% CI	% difference of mean Baseline* and FU-1	p-value Baseline* vs. FU- 1
**EQ-ED-5L**	**NSGI**	1.8	1.8-1.8	1.1	0.9-1.3	-39%	**0.001**
	**CT**	2.3	1.7-3.0	1.3	1.0-1.5	-44%	**0.002**
	**NAD**	1.9	0.9-3.0	1.6	0.4-2.8	-16%	0.633
**Rosenberg Self Esteem Scale**	**NSGI**	3.2	2.7-3.7	2.4	2.2-2.6	-25%	**0.008**
	**CT**	3.1	2.6-3.6	2.2	2.0-2.4	-29%	**0.009**
	**NAD**	3.3	2.6-4.0	2.6	2.4-2.8	-21%	**0.057**

*restricted to patients with a FU record

Abbreviations used in Tables 2 a and b

CT- *Chlamydia trachomatis*
NG- *Neisseria gonorrhoea*
NSGI- Non specific genital infection
NAD- No abnormality detected
FU- Follow-up

For peer review only - http://bmjopen.bmj.com/site/about/guidelines.xhtml

Ethical Approval

This study was approved by the London Bridge Research Ethics Committee (REC reference 16/LO/0955).

Acknowledgements

Thank you to the participants who took part in the study, our collaborators and the steering committee for their advice, including: Atlas Genetics, who were awarded the Innovate UK SBRI grant; Aquarius Population Health; Scientific Steering Committee (Chris Price, Kate Folkard, David Livermore), and the clinical leads at participating clinics (Richard Lau, Paul Lister).

Contributor Statement

STS conceived of the Study. RHT, SSF and EHE initiated the study design. AP, SSF, RHT and EHE implemented the study. MF and EHE provided statistical oversight. RHT wrote the initial draft of the manuscript. All authors contributed to and approved the final manuscript.

Competing Interests

The Applied Diagnostic Research and Evaluation Unit at St George's, University of London (STS, EHE, SF, AP), receives funding from the National Institute of Health Research (NIHR) i4i Programme (grant number II-LB-0214-20005), Atlas Genetics, Alere, Hologic Cepheid, SpeeDx, Sekisui and Becton Dickinson to develop Point of Care Tests for STIs. SF and EHE are members of Becton Dickenson's Advisory Board for the provision of sexual health in the UK. MF and RHT have no competing interests. The views expressed are those of the authors and not necessarily those of the NIHR, the NHS or the Department of Health.

Funding

This study was funded by an Innovate UK (SBRI grant no. 971452) awarded to Atlas Genetics Ltd. Neither Atlas Genetics nor Innovate UK had a role in the design, collection, management, analysis, interpretation of data or decision to submit a report.

Data Sharing Statement

Due to ethical concerns regarding the sensitive nature of the research, transcripts of qualitative interviews are not available for data sharing. All other data supporting this study are provided as supplementary information accompanying this paper.

REFERENCES

1 Table 4: All STI diagnoses & services by gender & sexual risk, 2010 - 2014. Public Health England. https://www.gov.uk/government/statistics/sexually-transmitted-infections-stis-annual-data-tables (accessed 29 Mar2016).

2 Horner P, Blee K, O'Mahony C, *et al.* 2015 UK National Guideline on the management of non-gonococcal urethritis. *Int J STD AIDS* 2016;**27**:85–96. doi:10.1177/0956462415586675

3 Pond MJ, Nori A V, Patel S, *et al.* Performance evaluation of automated urine microscopy as a rapid, non-invasive approach for the diagnosis of non-gonococcal urethritis. *Sex Transm Infect* 2015;**91**:165–70. doi:10.1136/sextrans-2014-051761

4 Smith R, Copas AJ, Prince M, *et al.* Poor sensitivity and consistency of microscopy in the diagnosis of low grade non-gonococcal urethritis. *Sex Transm Infect* 2003;**79**:487–90. doi:10.1136/sti.79.6.487

5 Menon-Johansson AS, McNaught F, Mandalia S, *et al.* Texting decreases the time to treatment for genital Chlamydia trachomatis infection. *Sex Transm Infect* 2006;**82**:49–51. doi:10.1136/sti.2004.014381

6 Bowie WR, Alexander ER, Stimson JB, *et al.* Therapy for nongonococcal urethritis: double-blind randomized comparison of two doses and two durations of minocycline. *Ann Intern Med* 1981;**95**:306–11.http://www.ncbi.nlm.nih.gov/pubmed/7271091 (accessed 24 Mar2016).

7 Horner P, Thomas B, Gilroy CB, *et al.* Role of Mycoplasma genitalium and Ureaplasma urealyticum in acute and chronic nongonococcal urethritis. *Clin Infect Dis* 2001;**32**:995–1003.

For peer review only - http://bmjopen.bmj.com/site/about/guidelines.xhtml

doi:10.1086/319594

8 Darroch J, Myers L, Cassell J. Sex differences in the experience of testing positive for genital chlamydia infection: a qualitative study with implications for public health and for a national screening programme. *Sex Transm Infect* 2003;**79**:372–3.http://www.ncbi.nlm.nih.gov/pubmed/14573831 (accessed 10 Mar2016).

9 Kangas I, Andersen B, Olesen F, *et al.* Psychosocial impact of Chlamydia trachomatis testing in general practice. *Br J Gen Pract* 2006;**56**:587–93.http://www.ncbi.nlm.nih.gov/pubmed/16882376 (accessed 10 Mar2016).

10 Robertson S, Williamson P. Men and health promotion in the UK: Ten years further on? *Health Educ J* 2005;**64**:293–301. doi:10.1177/001789690506400402

11 Cameron E, Bernardes J. Gender and disadvantage in health: men's health for a change. *Sociol Heal & Illn* 1998;**20**:673–93. doi:10.1111/1467-9566.00124

12 Connell R. *Masculinities*. University of California Press 2005. http://www.ucpress.edu/book.php?isbn=9780520246980 (accessed 28 Mar2017).

13 Beyond Men Behaving Badly: A Meta-Ethnography of Men's Perspectives on Psychological Distress and Help Seeking. doi:10.3149/jmh.1103.202

14 Collumbien M, Hawkes S. Missing men's messages: Does the reproductive health approach respond to men's sexual health needs? *Cult Health Sex* 2000;**2**:135–50. doi:10.1080/136910500300769

15 Banks I. No man's land: men, illness, and the NHS. *BMJ* 2001;**323**.http://www.bmj.com/content/323/7320/1058.long (accessed 31 Mar2017).

16 Rosaleen O, Hunt K, Hart G, *et al. Social science & medicine.* Elsevier Science 2005. http://econpapers.repec.org/article/eeesocmed/v_3a61_3ay_3a2005_3ai_3a3_3ap_3a503-516.htm (accessed 28 Mar2017).

17 Herdman M, Gudex C, Lloyd A, *et al.* Development and preliminary testing of the new five-level version of EQ-5D (EQ-5D-5L). *Qual Life Res* 2011;**20**:1727–36. doi:10.1007/s11136-011-9903-x

18 Petersen W. Society and the Adolescent Self-Image. Morris Rosenberg. Princeton University Press, Princeton, N.J., 1965. xii + 326 pp. $6.50. *Science (80-)* 1965;**148**:804–804. doi:10.1126/science.148.3671.804

19 Zimet GD, Dahlem NW, Zimet SG, *et al.* The Multidimensional Scale of Perceived Social Support. *J Pers Assess* 1988;**52**:30–41. doi:10.1207/s15327752jpa5201_2

20 Gottlieb SL, Stoner BP, Zaidi AA, *et al.* A prospective study of the psychosocial impact of a positive Chlamydia trachomatis laboratory test. *Sex Transm Dis* 2011;**38**:1004–11. doi:10.1097/OLQ.0b013e31822b0bed

21 Burkill S, Copas A, Couper MP, *et al.* Using the Web to Collect Data on Sensitive Behaviours: A Study Looking at Mode Effects on the British National Survey of Sexual Attitudes and Lifestyles. *PLoS One* 2016;**11**:e0147983. doi:10.1371/journal.pone.0147983

22 Council NR. *Nonresponse in Social Science Surveys*. Washington, D.C.: : National Academies Press 2013. doi:10.17226/18293

23 Marsden P V., Wright JD. *Handbook of survey research*. Emerald 2010. https://books.google.co.uk/books/about/Handbook_of_Survey_Research.html?id=mMPDPXpTP-0C (accessed 28 Mar2017).

24 Low N, Sterne JA, Barlow D. Inequalities in rates of gonorrhoea and chlamydia between black ethnic groups in south east London: cross sectional study. *Sex Transm Infect* 2001;**77**:15–20.http://www.ncbi.nlm.nih.gov/pubmed/11158686 (accessed 28 Mar2017).

25 Le Polain De Waroux O, Harris RJ, Hughes G, *et al.* The epidemiology of gonorrhoea in London: a Bayesian spatial modelling approach. *Epidemiol Infect* 2013;**142**:1–10. doi:10.1017/S0950268813000745

26 Murray BL. Qualitative research interviews: therapeutic benefits for the participants. *J Psychiatr Ment Health Nurs* 2003;**10**:233–6.http://www.ncbi.nlm.nih.gov/pubmed/12662341

For peer review only - http://bmjopen.bmj.com/site/about/guidelines.xhtml

(accessed 28 Mar2017).
27 Hutchinson SA, Wilson ME, Wilson HS. Benefits of Participating in Research Interviews. *Image J Nurs Scholarsh* 1994;**26**:161–6. doi:10.1111/j.1547-5069.1994.tb00937.x
28 Miyai T, Turner KR, Kent CK, *et al.* The psychosocial impact of testing individuals with no history of genital herpes for herpes simplex virus type 2. *Sex Transm Dis* 2004;**31**:517–21.http://www.ncbi.nlm.nih.gov/pubmed/15480111 (accessed 6 Dec2016).

For peer review only

For peer review only - http://bmjopen.bmj.com/site/about/guidelines.xhtml

Figure 1. Patient Pathway

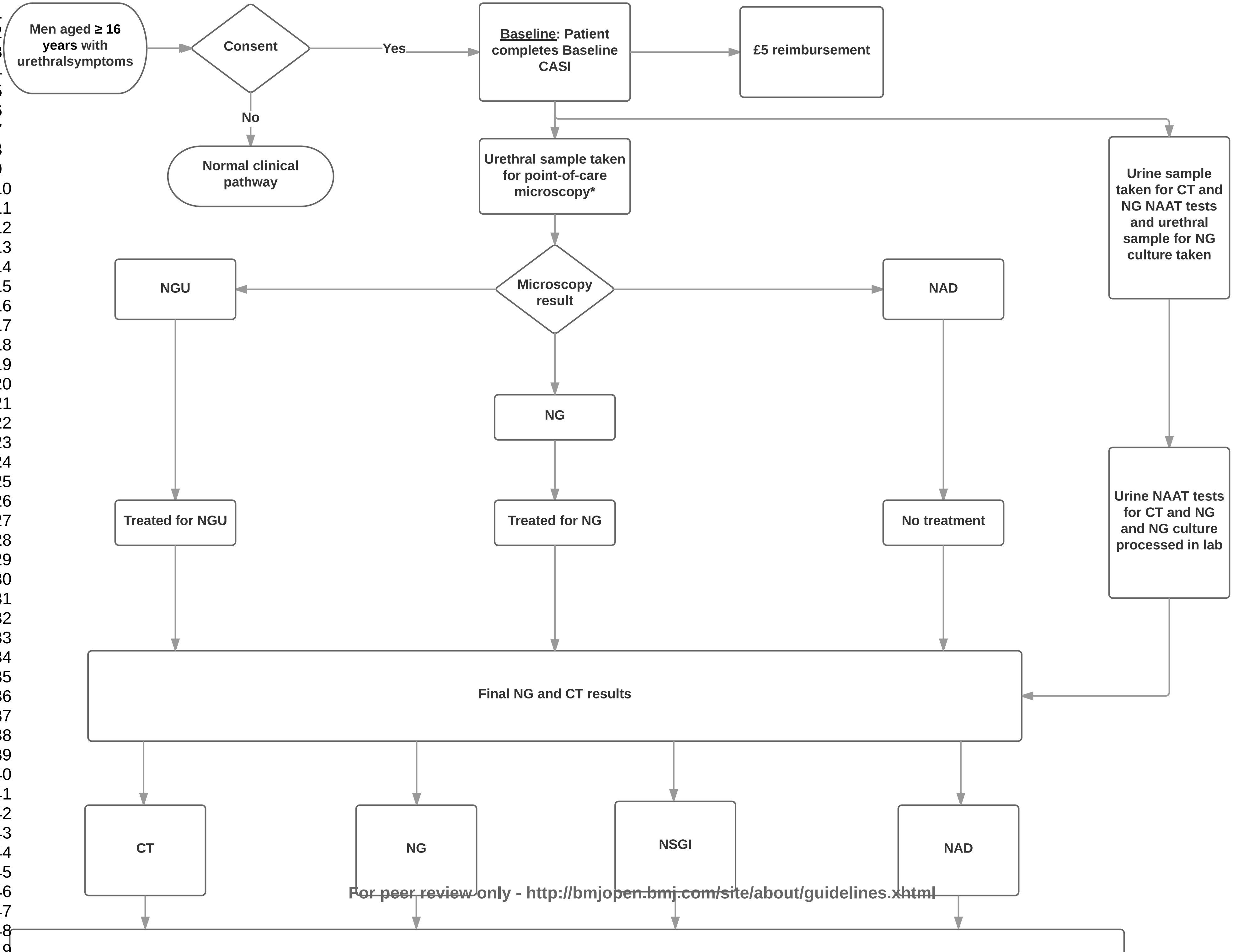

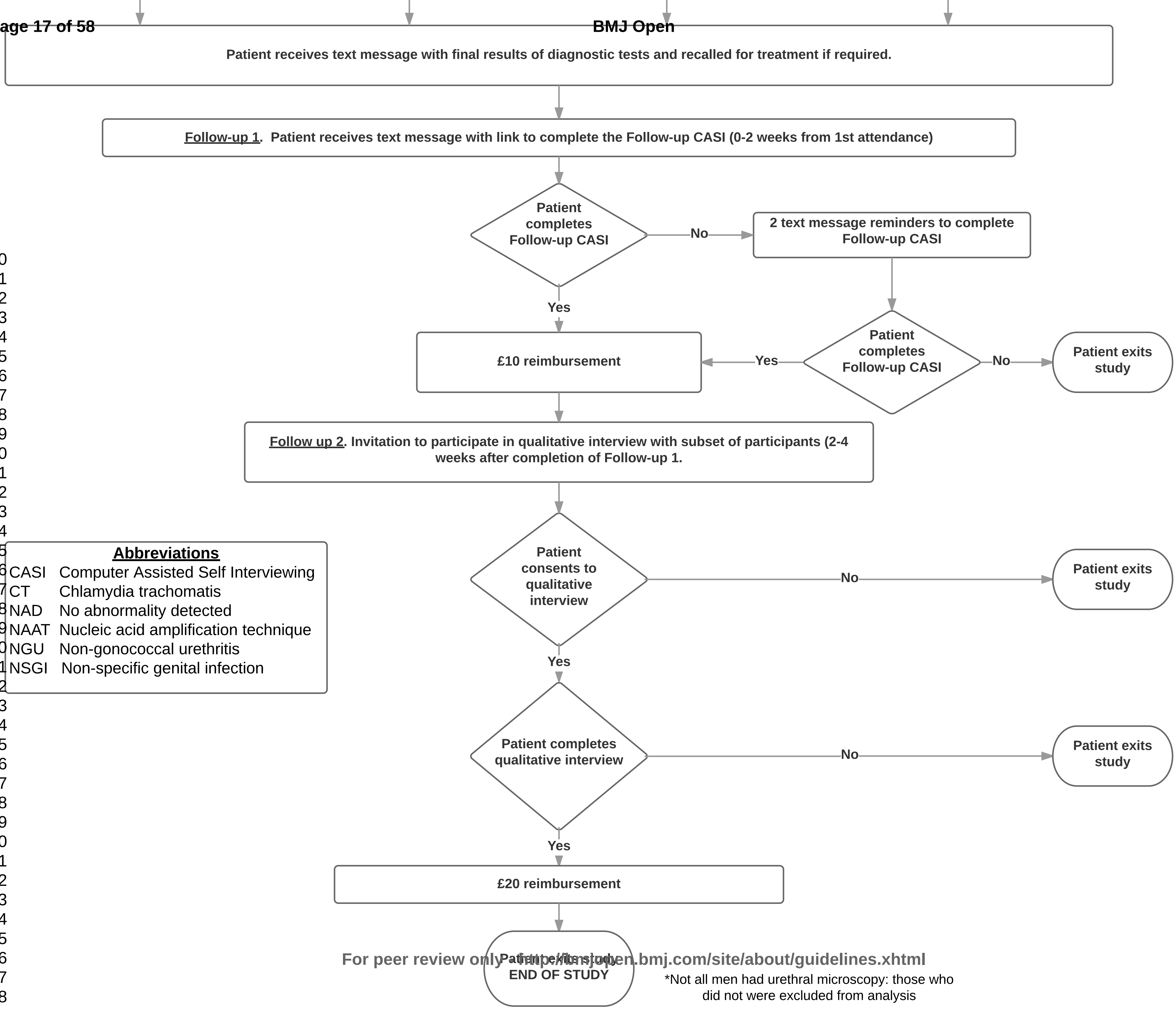

For peer review only - http://bmjopen.bmj.com/site/about/guidelines.xhtml

Figure 2. Enrolment and Follow-up Chart

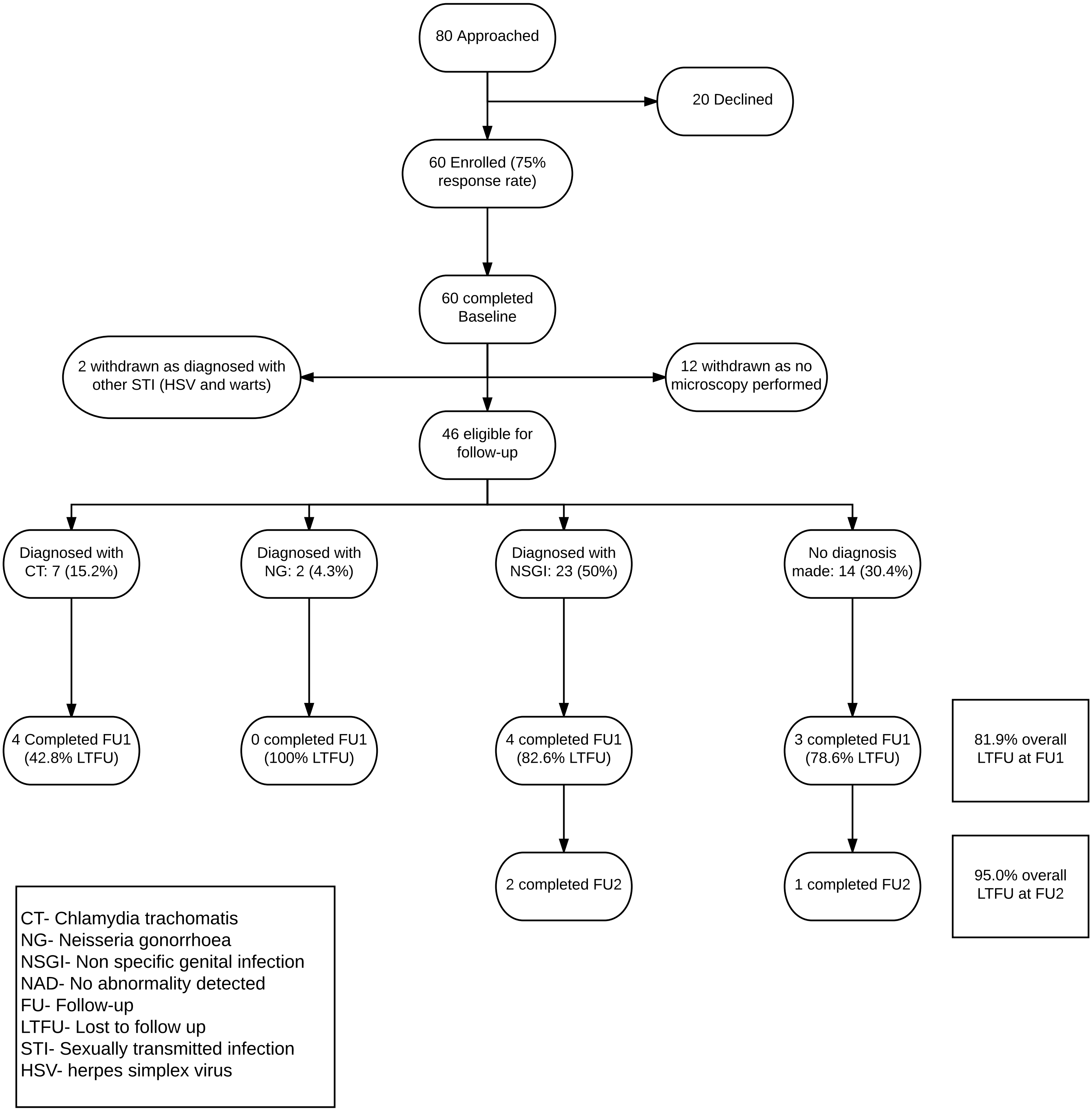

APPENDIX 1: Baseline CASI Questionnaire

The following questions are about your background

1. Are you in full time employment? Yes/No/ Prefer not to answer

2. How old were you when you left full-time education?
   - ☐ 16 years or less
   - ☐ 17 or 18 years
   - ☐ 19 years or over
   - ☐ Still in full-time education

3. What is your highest educational achievement?
   - ☐ No qualifications / No formal qualifications
   - ☐ 1-4 GCSEs or equivalent qualifications
   - ☐ 5 GCSEs or equivalent qualifications.
   - ☐ Apprenticeships
   - ☐ 2 or more A-levels or equivalent qualifications.
   - ☐ Bachelors degree or equivalent, and higher qualifications.
   - ☐ Other qualifications including foreign qualifications.

5. Do you have any of the following long-standing conditions? (Tick ALL that apply)
   - ☐ Deafness or severe hearing impairment
   - ☐ Blindness or partially sighted
   - ☐ A long-standing physical condition
   - ☐ A learning disability
   - ☐ A mental health condition
   - ☐ A long-standing illness, such as cancer, HIV, diabetes, chronic heart disease, or epilepsy

6. Does this condition(s) cause you difficulty with any of the following? (Tick ALL that apply)
   - ☐ Everyday activities that people your age can usually do
   - ☐ At work, in education, or training
   - ☐ Access to buildings, streets or vehicles
   - ☐ Reading or writing
   - ☐ People's attitudes to you because of your condition
   - ☐ Communicating, mixing with others, or socialising
   - ☐ Any other activity
   - ☐ No difficulty with any of these

7. To which of these ethnic groups would you say you belong? (Tick ONE only)

a. WHITE
   - ☐ English/Welsh/Scottish/Northern Irish/British
   - ☐ Irish
   - ☐ Gypsy or Irish Traveller

For peer review only

- ☐ Any other White background, write in...

b. MIXED / MULTIPLE ETHNIC GROUPS
- ☐ White and Black Caribbean
- ☐ White and Black African
- ☐ White and Asian
- ☐ Any other Mixed/multiple ethnic background, write in...

c. ASIAN / ASIAN BRITISH
- ☐ Indian
- ☐ Pakistani
- ☐ Bangladeshi
- ☐ Chinese
- ☐ Any other Asian background, write in...

d. BLACK / AFRICAN / CARIBBEAN / BLACK BRITISH
- ☐ African
- ☐ Caribbean
- ☐ Any other Black / African / Caribbean background, write in...

e. OTHER ETHNIC GROUP
- ☐ Arab
- ☐ Any other ethnic group, write in...

The following are questions about recent infections

8. Have you ever been diagnosed with a sexually transmitted infection? If so which?
TICK ALL THAT APPLY
- ☐ Chlamydia
- ☐ Gonorrhoea
- ☐ Genital Herpes
- ☐ Non Specific Genital Infection (NSGI)
- ☐ Genital Warts
- ☐ Syphilis
- ☐ HIV
- ☐ Other (write in)
- ☐ Prefer not to answer

The following questions are about relationships and break-ups

9. At present are you...
- ☐ Married and living with wife
- ☐ Cohabiting but not married
- ☐ Separated, divorced, or, widowed
- ☐ Single (that is never married or never registered in a same-sex civil partnership)
- ☐ Prefer not to answer

9a. How long have you been in this relationship for?
- ☐ 0 – 6 months

For peer review only

- More than 6 months but less than a year
- 1 – 2 years
- 2 or more years
- Prefer not to answer

10. In the last 30 days, have you broken up with someone you considered yourself to be in a relationship with (for example, your girlfriend or wife)?
- Yes
- No
- Prefer not to answer

On the following screens please tap the statement which best describes your own health state today.

11. MOBILITY
- I have no problems in walking about
- I have slight problems in walking about
- I have moderate problems in walking about
- I have severe problems in walking about
- I am unable to walk about

12. SELF-CARE
- I have no problems washing or dressing myself
- I have slight problems washing or dressing myself
- I have moderate problems washing or dressing myself
- I have severe problems washing or dressing myself
- I am unable to wash or dress myself

13. USUAL ACTIVITIES (e.g. work, Study, housework, family or leisure activities)
- I have no problems doing my usual activities
- I have slight problems doing my usual activities
- I have moderate problems doing my usual activities
- I have severe problems doing my usual activities
- I am unable to do my usual activities

14. PAIN / DISCOMFORT
- I have no pain or discomfort
- I have slight pain or discomfort
- I have moderate pain or discomfort
- I have severe pain or discomfort
- I have extreme pain or discomfort

15. ANXIETY / DEPRESSION
- I am not anxious or depressed
- I am slightly anxious or depressed

For peer review only

- ☐ I am moderately anxious or depressed
- ☐ I am severely anxious or depressed
- ☐ I am extremely anxious or depressed

We would like to know how good or bad your health is TODAY.

This scale is numbered from 0 to 100.
100 means the best health you can imagine. 0 means the worst health you can imagine.

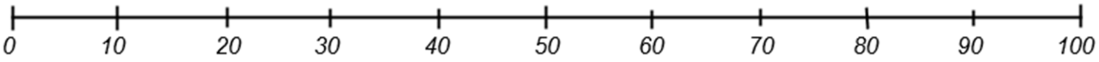

16. Please write the number you think best describes how you feel today. ______________

Below is a list of statements dealing with your general feelings about yourself. Please indicate how strongly you agree or disagree with each statement.

Strongly Agree   Agree   Disagree   Strongly Disagree

17. On the whole, I am satisfied with myself.
18. At times I think I am no good at all.
19. I feel that I have a number of good qualities.
20. I am able to do things as well as most other people.
21. I feel I do not have much to be proud of.
22. I certainly feel useless at times.
23. I feel that I'm a person of worth, at least on an equal plane with others.
24. I wish I could have more respect for myself.
25. All in all, I am inclined to feel that I am a failure.
26. I take a positive attitude toward myself.

Below is a list of statements dealing with your general feelings about your support from people around you. Please indicate how strongly you agree or disagree with each statement.
1 Very Strongly Disagree, 2 Strongly Disagree, 3 Mildly Disagree, 4 Neutral, 5 Mildly Agree, 6 Strongly Agree, 7 Very strongly agree

27. There is a special person who is around when I am in need
28. There is a special person with whom I can share my joys and sorrows
29. My family really tries to help me
30. I get all the emotional help and support I need from my family
31. I have a special person who is a real source of comfort to me
32. My friends really try to help me

For peer review only

33. I can count on my friends when things go wrong
34. I can talk about my problems with my family
35. I have friends with whom I can share my joys and sorrows
36. There is a special person in my life who cares about my feelings
37. My family is willing to help me make decisions.
38. I can talk about my problems with my friends.

We would now like to ask you if you have any feedback for us on how you found completing this questionnaire

39. How did you feel about completing this series of questions? Please write any comments in the space below.

____

____

____

Thank-you for completing this questionnaire
If you would like to speak to someone about the issues raised in this questionnaire please contact us.

For peer review only

APPENDIX 2: FU-1 CASI

On the following screens please tap the statement which best describes your own health state today.

1. MOBILITY
- ☐ I have no problems in walking about
- ☐ I have slight problems in walking about
- ☐ I have moderate problems in walking about
- ☐ I have severe problems in walking about
- ☐ I am unable to walk about

2. SELF-CARE
- ☐ I have no problems washing or dressing myself
- ☐ I have slight problems washing or dressing myself
- ☐ I have moderate problems washing or dressing myself
- ☐ I have severe problems washing or dressing myself
- ☐ I am unable to wash or dress myself

3. USUAL ACTIVITIES (e.g. work, Study, housework, family or leisure activities)
- ☐ I have no problems doing my usual activities
- ☐ I have slight problems doing my usual activities
- ☐ I have moderate problems doing my usual activities
- ☐ I have severe problems doing my usual activities
- ☐ I am unable to do my usual activities

4. PAIN / DISCOMFORT
- ☐ I have no pain or discomfort
- ☐ I have slight pain or discomfort
- ☐ I have moderate pain or discomfort
- ☐ I have severe pain or discomfort
- ☐ I have extreme pain or discomfort

5. ANXIETY / DEPRESSION
- ☐ I am not anxious or depressed
- ☐ I am slightly anxious or depressed
- ☐ I am moderately anxious or depressed
- ☐ I am severely anxious or depressed
- ☐ I am extremely anxious or depressed

We would like to know how good or bad your health is TODAY.

This scale is numbered from 0 to 100.

For peer review only

100 means the best health you can imagine. 0 means the worst health you can imagine.

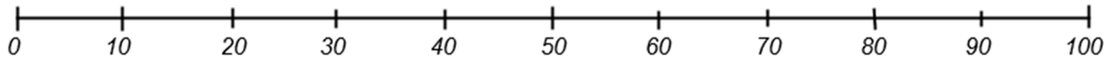

6. Please write the number you think best describes how you feel today. ______________

7. After you had received all your test results from your most recent visit to the GUM clinic were you diagnosed with any of the following infections: Chlamydia, Gonorrhoea or Non-specific urethritis (NSU)? ?
   - ☐ Yes
   - ☐ No
   - ☐ Not sure

The following questions are about your reaction after you were told your test was positive

1= Strongly Disagree 2 = Disagree 3 = Neutral 4 = Agree 5 = Strongly Agree

After I was told that my test was positive:

8. I talked to my friend(s) about it.	1	2	3	4	5
9. I do not think it was a big deal.	1	2	3	4	5
10. I was/am scared to tell my partner(s).	1	2	3	4	5
11. It does not change the way I feel about myself.	1	2	3	4	5
12. My partner thinks that I cheated on him or her.	1	2	3	4	5
13. I am not worried since it is curable.	1	2	3	4	5
14. I feel like I did not take care of myself.	1	2	3	4	5
15. I feel betrayed by my partner.	1	2	3	4	5
16. I worry that it will not go away.	1	2	3	4	5
17. I feel guilty that I could have given someone my infection	1	2	3	4	5
18. It has not changed my relationship with my partner.	1	2	3	4	5
19. I am not very proud of my actions.	1	2	3	4	5
20. I do not blame myself.	1	2	3	4	5
21. I think that my partner cheated on me.	1	2	3	4	5
22. I worry that I might not be able to have children in the future.	1	2	3	4	5
23. I do not really care that much.	1	2	3	4	5
24. I am angry.	1	2	3	4	5
25. I feel really bad about myself.	1	2	3	4	5
26. It does not change how my friends feel about me.	1	2	3	4	5
27. It will be hard for me to trust future partners.	1	2	3	4	5
28. I am not worried about getting it again.	1	2	3	4	5
29. I feel dirty.	1	2	3	4	5
30. I broke up with my partner.	1	2	3	4	5

For peer review only

31. I worry about what other sexually transmitted infections I could have been exposed to.	1	2	3	4	5
32. I feel really embarrassed.	1	2	3	4	5

The next questions are about how you feel generally about the infection you were diagnosed with

33. I think about the infection I was diagnosed with a lot.
1= Strongly Disagree 2 = Disagree 3 = Neutral 4 = Agree 5 = Strongly Agree

34. I am concerned about the infection I was diagnosed with.
1= Strongly Disagree 2 = Disagree 3 = Neutral 4 = Agree 5 = Strongly Agree

Below is a list of statements dealing with your general feelings about yourself. Please indicate how strongly you agree or disagree with each statement.

Strongly Agree Agree Disagree Strongly Disagree

35. On the whole, I am satisfied with myself.
36. At times I think I am no good at all.
37. I feel that I have a number of good qualities.
38. I am able to do things as well as most other people.
39. I feel I do not have much to be proud of.
40. I certainly feel useless at times.
41. I feel that I'm a person of worth, at least on an equal plane with others.
42. I wish I could have more respect for myself.
43. All in all, I am inclined to feel that I am a failure.
44. I take a positive attitude toward myself.

The following questions are about relationships and break-ups

45. At present are you...
- ☐ married and living with wife
- ☐ cohabiting but not married
- ☐ separated, divorced, or, widowed
- ☐ single (that is never married)
- ☐ prefer not to answer

45a. How long have you been in this relationship for?
- ☐ 0 – 6 months
- ☐ More than 6 months but less than a year
- ☐ 1 – 2 years
- ☐ 2 or more years
- ☐ Prefer not to answer

For peer review only

46. In the last 30 days, have you broken up with someone you considered yourself to be in a relationship with (for example, your girlfriend or wife)?

- ☐ Yes
- ☐ No
- ☐ Prefer not to answer

The next questions are about your most recent visit to the GUM Clinic

47. Have you ever visited this GUM Clinic before for the same condition?

- ☐ Yes
- ☐ No
- ☐ Prefer not to answer

48. Did a member of staff explain the results of the tests in a way you could understand?

- ☐ Yes definitely
- ☐ Yes, to some extent
- ☐ No
- ☐ Not sure, can't remember
- ☐ I was told I would get the results at a later date
- ☐ I was never told the results of the tests

49. While you were in the GUM Clinic how much information about your condition or treatment was given to you?

- ☐ Not enough
- ☐ Right amount
- ☐ Too much
- ☐ I was not given any information about my treatment or condition

50. Was the main reason you went to the GUM Clinic dealt with to your satisfaction?

- ☐ Yes, completely
- ☐ Yes, to some extent
- ☐ No

The next questions are about the symptoms you had when attending the clinic (the time you were first asked to join this study)

51. Please choose the option that best fits how you feel

- ☐ My symptoms have completely gone away
- ☐ My symptoms are better but not completely
- ☐ My symptoms are the same
- ☐ My symptoms are worse than they were before
- ☐ Don't know/not sure
- ☐ Prefer not to answer

For peer review only

We would now like to ask you if you have any feedback for us on how you found completing this questionnaire

52. How did you feel about completing this series of questions? Please write any comments in the space below.

____
____
____

Thank-you for completing this questionnaire
If you would like to speak to someone about the issues raised in this questionnaire please contact us.

For peer review only

APPENDIX 3: Individual in-depth interview Topic Guide

Topics

Life history / framing (~5 minutes)

Please tell me a bit about yourself.

What is the most important thing you can tell me about yourself?

Probes:

Hobbies/interests

Goals

Tell me about your family.

Who do you include in your family? Partner/spouse? Father/mother? Grandparents? Children?

What sorts of things do you do with your day? / Describe a typical day in your life.

Probes:

Are you in school/university?

What do you do for money?

Clinical experience (~25 minutes)

Please describe how you were feeling while you were at the clinic on the day you were asked to take part in this study.

Probes:

Describe the reasons why you came to clinic

(If symptoms) What affect (if any) did your symptoms have on your day-to-day life?

How did it go/ how did you feel about the visit?

How long were you waiting to be seen? Was this expected /unexpected?

Which infections were you tested for?

What did the doctor tell you they thought you had? (i.e. diagnosis, if any)

Now let's discuss your diagnosis.

What thoughts were going through your mind when you were told you had NSU/Ng/possibly an STI (use patients' own description/language for their diagnosis)?

Probes:

How did you feel about your diagnosis?

For peer review only

Have you been diagnosed with NSU/Ng before? Any other infections? If yes, please describe any differences in your feelings from the first time you were diagnosed compared with this recent diagnosis.

Did you tell anyone (partner/family members/friends) your diagnosis? If yes, who and why? What was their reaction?

Research experience (~15 minutes)

We want to make sure that the research we conduct is done in the best way possible for patients. This next section of our interview is based on your experience with this research study so far. Please remember that your answers to these questions will not affect your clinical care, your legal rights, or future invitations to participate in research. It is important that you speak freely so that we can improve future research in this area.

How did you feel about your participation in the research?

Probes:

Would you consider involving yourself in this kind of research in the future? Why/why not?

Were you able to complete the surveys?

Did you have any problem accessing the CASI? (Logistical/technical problems) If so, what were the problems you faced?

Did you feel comfortable accessing the CASI (inside/outside of clinic)? Why/why not?

How did you feel about the questionnaires?

Discuss each questionnaire individually in turn: demographic and clinic experience questions; EQ5D-5L; social support scale; self-esteem scale:

Did you feel these questions were relevant?

Did you feel comfortable answering these questions? Why/why not?

Discuss any suggestions you may have for improvement of these questionnaires

Anything else?

Thanks

Reimbursement

For peer review only

APPENDIX 2: Full list of themes for Qualitative Interview (FU-2)

**List of nodes**
antibiotics
attitudes to safe sex and sexual health
consultation
different SHCs
familiarity with SHC
feelings about attending SHC
feelings about being interviewed
feelings about participation in the research
feelings about positive diagnosis
future research
HIV vs. other STIs
interview
invasive tests
NSGI vs. CT
obstacles to participation in research
perception of CT
reaction to NSGI
reasons for participating in research
relationship
reasons for attending SHC
results
risk perception
social support
STI prevention
suggestions for research
survey - was it easy
survey - was it engaging
survey - was it relevant
survey comfort
treatment
waiting for results
waiting time in SHC

For peer review only

STROBE Statement—checklist of items that should be included in reports of observational studies

	Item No.	Recommendation	Page No.	Relevant text from manuscript
**Title and abstract**	1	(*a*) Indicate the study's design with a commonly used term in the title or the abstract	1	**Health related quality of life and psychosocial impacts of a diagnosis of non-specific genital infection in symptomatic men attending UK Sexual Health clinics: a feasibility study.**
		(*b*) Provide in the abstract an informative and balanced summary of what was done and what was found	1-2	**Introduction:** Non-specific genital infection (NSGI; non-*Chlamydia trachomatis* (CT), non-*Neisseria gonorrhoeae* (NG) associated urethritis) is a common diagnosis in symptomatic heterosexual men attending UK Sexual Health Clinics (SHCs) but little is known about the psychosocial impact of this diagnosis. **Methods:** We conducted a feasibility study to measure psychosocial

For peer review only - http://bmjopen.bmj.com/site/about/guidelines.xhtml

impact of an NSGI diagnosis among symptomatic heterosexual men attending SHCs compared to the impact of a diagnosis of CT, NG or no abnormalities detected (NAD). Participants completed a Computer Assisted Self Interview (CASI) including validated health-related quality-of-life (HRQoL) and self-esteem measures before diagnostic testing and two-weeks after receiving test results, and a qualitative interview. We compared scores between diagnostic groups using paired t-tests, qualitative data were analysed thematically, and feasibility was assessed by process analysis.

Results:

60 men completed the baseline CASI (75% response rate). 46 (76.6%) were eligible for follow-up; of these, 11 (23.9%)

For peer review only - http://bmjopen.bmj.com/site/about/guidelines.xhtml

completed the follow-up CASI, and 3 (27.3%) completed the qualitative interview. 81.7% of participants left CASI feedback at baseline: 73.5% reported the questionnaire as 'fine' or 'very good'. Qualitative interview participants reported the study was acceptable. All patients completing FU-1 showed measurable decreases from baseline to FU-1 in self-esteem (CT p=0.009; NSGI p=0.008; NAD p=0.057). Measurable decreases in HRQoL from baseline to FU-1 were found among participants with CT (p=0.002) or NSGI (p=0.001). We did not find differences in baseline sociodemographic factors between participants who completed FU-1 and those that did not.

Conclusions:
Although most participants indicated study acceptability at

For peer review only - http://bmjopen.bmj.com/site/about/guidelines.xhtml

baseline, and we employed measures to increase retention (CASI questionnaires, reminder messages, a focus on men's health), attrition was very high. We found that heterosexual men attending SHCs with symptoms of urethritis may experience negative psychosocial impacts regardless of their diagnosis which warrants further investigation. We recommend exploring novel strategies to decrease attrition in future studies.

Key messages:

1. Non-specific genital infection (NSGI) is frequently diagnosed in symptomatic heterosexual men attending sexual health clinics but little is known about this impact of this diagnosis.
2. Men's self-esteem and quality of life scores were significantly lower after a diagnosis of Chlamydia trachomatis or NSGI compared to before undergoing

For peer review only - http://bmjopen.bmj.com/site/about/guidelines.xhtml

				testing for infections.<br>3. Computer assisted self-interview appears to be an acceptable method to assess heterosexual men's' views in sexual health but participant retention in longitudinal follow-up was challenging.
**Introduction**				
Background/rationale	2	Explain the scientific background and rationale for the investigation being reported	3	Non-specific Genital Infection (NSGI) or, non-*Chlamydia trachomatis* (CT), non-*Neisseria gonorrhoeae* (NG) associated urethritis (NGU), is the third most frequently diagnosed condition in heterosexual men attending sexual health clinics (SHCs) in the United Kingdom (UK), with 30,381 cases/year compared to CT (42,483/year) and first presentation of genital warts (32,656/year) [1]. NSGI is a diagnosis of exclusion; men with urethral symptoms are screened with urethral microscopy in SHCs, if this

For peer review only - http://bmjopen.bmj.com/site/about/guidelines.xhtml

For peer review only

demonstrates urethritis with no gonococci, a provisional diagnosis of NGU is made [2]. Urinary Nucleic Acid Amplification Tests (NAATs) are sent for NG and CT, if these are both negative, the diagnosis is recorded as NSGI.

Although a common condition, NSGI remains poorly understood. NSGI can be caused by a variety of sexually transmitted infections (STIs), including *Mycoplasma genitalium* (MG) and *Trichomonas vaginalis* (TV), which are not routinely tested for in SHCs, and many cases have an uncertain aetiology [2]. Microscopy, on which the diagnosis rests, has poor specificity for known causes of urethritis [3] and is subject to considerable observer variation [4].

For peer review only - http://bmjopen.bmj.com/site/about/guidelines.xhtml
For peer review only

National guidelines recommend that men diagnosed with NGU are informed they may have an STI, given same day empirical antibiotics, advised their partners should attend for STI testing and empirical treatment, and to abstain from sex until treatment is completed [2]. Men receive CT/NG NAAT results usually within 8 days [5]. Following first-line antibiotics for NSGI, 10%–20% of men return because of persistent symptoms [6,7].

Men who undergo STI testing and are diagnosed with CT report negative impacts on their psychosocial wellbeing [8,9], however, to our knowledge, there are no published data on the psychosocial impact of NSGI diagnosis among men.

Men are reportedly poor users of health services, largely due to

For peer review only

For peer review only - http://bmjopen.bmj.com/site/about/guidelines.xhtml

culturally dominant ideals of masculinity that equate illness with weakness, which may impede care-seeking. [10–13] In sexual healthcare, there has traditionally been a focus on female reproductive health [14], and this may further discourage male attendance [14,15] However, men are willing to respond openly to questions about their sexual health, particularly when enquiries are focused specifically on men's needs. [14,16]

Because of a lack of models to measure the psychosocial impact of an NSGI diagnosis among men, and the potential difficulty in recruiting and retaining this group in sexual health studies, we designed a small-scale longitudinal mixed-method feasibility study to inform on our research methods and better understand the

For peer review only

For peer review only - http://bmjopen.bmj.com/site/about/guidelines.xhtml

				psychosocial and health related quality of life (HRQoL) status of symptomatic heterosexual men before and after receiving a diagnosis of NSGI, CT, NG or nothing abnormal detected (NAD).
Objectives	3	State specific objectives, including any prespecified hypotheses	3	Because of a lack of models to measure the psychosocial impact of an NSGI diagnosis among men, and the potential difficulty in recruiting and retaining this group in sexual health studies, we designed a small-scale longitudinal mixed-method feasibility study to inform on our research methods and better understand the psychosocial and health related quality of life (HRQoL) status of symptomatic heterosexual men before and after receiving a diagnosis of NSGI, CT, NG or nothing abnormal detected (NAD).
**Methods**				
Study design	4	Present key elements of study design early in the paper	4	We conducted a longitudinal study in symptomatic heterosexual men using patient

For peer review only - http://bmjopen.bmj.com/site/about/guidelines.xhtml

For peer review only

				completed questionnaires assessing HRQoL and psychosocial status before and after receiving STI test results, and individual qualitative interviews to evaluate experiences at the SHC and acceptability of study methods.
Setting	5	Describe the setting, locations, and relevant dates, including periods of recruitment, exposure, follow-up, and data collection	4	Eligibility criteria were: heterosexual men presenting with urethral symptoms (dysuria, discharge or urethral discomfort), aged ≥16 years at either of two participating London SHCs between September - October 2016. We excluded men who were asymptomatic, not available for follow-up, and who were unable to understand English.<br><br>Following informed consent, participants were invited to complete a baseline computer assisted self-interview (CASI) assessing psychosocial and HRQoL status in clinic prior to

For peer review only - http://bmjopen.bmj.com/site/about/guidelines.xhtml

				consultation, a follow-up CASI after receipt of all test results 1-2 weeks after baseline (FU-1) and a qualitative interview 2-4 weeks after baseline (FU-2).<br><br>CASIs were optimised for web-enabled devices including smartphones. We offered participants the choice to complete CASIs using their own device or a study tablet-device and use of a private room in clinic. Study staff sent ≤2 text message reminders for each follow-up. Participants who did not complete FU-1 were considered ineligible for FU-2. Participants received gift voucher reimbursements: £5 (baseline), £10 (FU-1), £20 (FU-2).
Participants	6	(*a*) *Cohort study*—Give the eligibility criteria, and the sources and methods of selection of participants. Describe methods of follow-up<br>*Case-control study*—Give the eligibility criteria, and the sources and methods of case ascertainment and control selection. Give the rationale for the choice of cases and controls<br>*Cross-sectional study*—Give the eligibility criteria, and the sources and methods of selection of	4	Eligibility criteria were: heterosexual men presenting with urethral symptoms (dysuria, discharge or urethral discomfort),

For peer review only

		participants		aged ≥16 years at either of two participating London SHCs between September - October 2016. We excluded men who were asymptomatic, not available for follow-up, and who were unable to understand English. Following informed consent, participants were invited to complete a baseline computer assisted self-interview (CASI) assessing psychosocial and HRQoL status in clinic prior to consultation, a follow-up CASI after receipt of all test results 1-2 weeks after baseline (FU-1) and a qualitative interview 2-4 weeks after baseline (FU-2).
		(*b*) *Cohort study*—For matched studies, give matching criteria and number of exposed and unexposed<br>*Case-control study*—For matched studies, give matching criteria and the number of controls per case		
Variables	7	Clearly define all outcomes, exposures, predictors, potential confounders, and effect modifiers. Give diagnostic criteria, if applicable	4-5	**Diagnostic Criteria**<br>We categorised participants into four diagnostic groups based on microscopy and NAAT results: NSGI: NGU diagnosis on

For peer review only - http://bmjopen.bmj.com/site/about/guidelines.xhtml

				urethral microscopy and CT/NG NAATs negative; NG: NG on urethral microscopy and/or positive NAAT; CT: positive NAAT; NAD: no urethritis on microscopy and CT/NG NAATs negative.<br><br>**Outcome measures**<br>*Primary outcome:* Qualitative and quantitative process data on recruitment and retention rates at baseline and follow-up to inform on feasibility. Evaluation of potential relevance of questions validated in other populations for men diagnosed with NSGI.<br><br>*Secondary outcomes:* Percentage change from baseline to FU-1 in mean EQ-5D-5L and Rosenberg Self-Esteem Scale in men diagnosed with CT, NG, NSGI or NAD.
Data sources/ measurement	8*	For each variable of interest, give sources of data and details of methods of assessment (measurement). Describe comparability of assessment methods if there is more than one group	4-5	The participant pathway is

For peer review only - http://bmjopen.bmj.com/site/about/guidelines.xhtml

For peer review only

detailed in Figure 1. After completion of the baseline CASI, any participants diagnosed with an STI other than CT, NG or NSGI, or who did not have both urethral microscopy and urinary NAATs, were withdrawn from the study. Once participants were notified of their NAAT CT/NG results by the SHC (phone call/ text message), the research team texted participants an electronic link to the FU-1 CASI.

Questionnaire design

Baseline and follow-up CASIs included validated scales measuring psychosocial status and HRQoL and additional questions. The EQ-5D-5L scale [17] as a measure of HRQoL and the Rosenberg Self-Esteem scale [18] as a measure of potential stigmatising effects of screening for STIs, were used in both baseline and FU-1 CASIs.

For peer review only

For peer review only - http://bmjopen.bmj.com/site/about/guidelines.xhtml

				In addition to the above, the baseline CASI included the Multidimensional Scale of Social Support [19] to assess coping resources and questions on sociodemographic and current relationship status, and the FU-1 CASI included a question on current relationship status and a panel of questions previously developed to assess impact of CT diagnosis in women, [20] which we modified for use in men by removing a pregnancy-related question. Participants were asked to indicate acceptability of the CASIs (free-text response) at baseline and FU-1. (Baseline and FU-1 questionnaires and FU-2 topic guide: Appendix 1, Supplementary Information.)
Bias	9	Describe any efforts to address potential sources of bias	5	**Data analysis**<br>*Questionnaire data:*<br>Scores for psychosocial and

For peer review only

For peer review only - http://bmjopen.bmj.com/site/about/guidelines.xhtml

HRQoL scales were calculated according to published methods [18][17][19]. For all eligible men, mean and 95% Confidence Intervals of baseline scores for EQ-5D-5L, Rosenberg Self-Esteem and Multidimensional Social Support Scales were compared in men diagnosed with CT, NG, NSGI and NAD, using paired t-tests. To test for non-response bias we compared sociodemographic data of those who completed both baseline and follow-up to those who were lost to follow-up (LTFU) using Pearson's Chi^2 tests and Kruskal-Wallis tests. Paired t-tests were used to assess differences in mean score for EQ-5D-5L and Rosenberg Self-Esteem Scales between baseline and follow-up in men diagnosed with CT, NG, NSGI and NAD. Statistical significance was set at $p<0.05$. Statistical analysis was carried out using STATA

For peer review only - http://bmjopen.bmj.com/site/about/guidelines.xhtml

For peer review only

				version 13.1 (StataCorp, 2015).<br><br>*Qualitative data:* Audio recordings were transcribed and transcripts checked for accuracy against the recording. Cleaned transcripts were then imported into NVivo (Version 10, 2012) for analysis. Two researchers assigned themes to text independently, with discrepancies resolved by consensus before final themes reported. All names of participants presented here are pseudonyms.
Study size	10	Explain how the study size was arrived at	5	As this was a feasibility study with no precedent, no formal sample size could be calculated. We planned to approach 100 patients at baseline as a convenience target, and assuming approximately 50% refusal, we expected 50-60 patients to be enrolled and estimated ~50% attrition at each follow-up.

For peer review only - http://bmjopen.bmj.com/site/about/guidelines.xhtml

Continued on next page

For peer review only

Quantitative variables	11	Explain how quantitative variables were handled in the analyses. If applicable, describe which groupings were chosen and why	5	Scores for psychosocial and HRQoL scales were calculated according to published methods [18][17][19]. For all eligible men, mean and 95% Confidence Intervals of baseline scores for EQ-5D-5L, Rosenberg Self-Esteem and Multidimensional Social Support Scales were compared in men diagnosed with CT, NG, NSGI and NAD, using paired t-tests. To test for non-response bias we compared sociodemographic data of those who completed both baseline and follow-up to those who were lost to follow-up (LTFU) using Pearson's Chi^2 tests and Kruskal-Wallis tests. Paired t-tests were used to assess differences in mean score for EQ-5D-5L and Rosenberg Self-Esteem Scales between baseline and follow-up in men diagnosed with CT, NG, NSGI and NAD. Statistical significance was set at $p<0.05$. Statistical analysis was carried out using STATA version 13.1 (StataCorp, 2015).

For peer review only - http://bmjopen.bmj.com/site/about/guidelines.xhtml

Statistical methods	12	(*a*) Describe all statistical methods, including those used to control for confounding	5	Scores for psychosocial and HRQoL scales were calculated according to published methods [18][17][19]. For all eligible men, mean and 95% Confidence Intervals of baseline scores for EQ-5D-5L, Rosenberg Self-Esteem and Multidimensional Social Support Scales were compared in men diagnosed with CT, NG, NSGI and NAD, using paired t-tests. To test for non-response bias we compared sociodemographic data of those who completed both baseline and follow-up to those who were lost to follow-up (LTFU) using Pearson's Chi^2 tests and Kruskal-Wallis tests. Paired t-tests were used to assess differences in mean score for EQ-5D-5L and Rosenberg Self-Esteem Scales between baseline and follow-up in men diagnosed with CT, NG, NSGI and NAD. Statistical significance was set at $p<0.05$. Statistical analysis was carried out using STATA version 13.1 (StataCorp, 2015).

For peer review only - http://bmjopen.bmj.com/site/about/guidelines.xhtml

		(*b*) Describe any methods used to examine subgroups and interactions		
		(*c*) Explain how missing data were addressed	6	Pearson's Chi^2 tests and Kruskal-Wallis test results for non-response bias were not statistically significant so we assumed data were missing at random.
		(*d*) *Cohort study*—If applicable, explain how loss to follow-up was addressed<br>*Case-control study*—If applicable, explain how matching of cases and controls was addressed<br>*Cross-sectional study*—If applicable, describe analytical methods taking account of sampling strategy		
		(*e*) Describe any sensitivity analyses		
**Results**				
Participants	13*	(a) Report numbers of individuals at each stage of study—eg numbers potentially eligible, examined for eligibility, confirmed eligible, included in the study, completing follow-up, and analysed	5	We approached 80 patients, of whom 60 (75%) enrolled and completed the baseline CASI. 46 (76.6%) were eligible for follow-up; of these, 11 (23.9%) completed the FU-1 CASI, and 3 of these (27.3%) completed FU-2 (Figure 2.)
		(b) Give reasons for non-participation at each stage		See Figure 2
		(c) Consider use of a flow diagram		See Figure 2
Descriptive data	14*	(a) Give characteristics of study participants (eg demographic, clinical, social) and information on exposures and potential confounders	6	Participants' sociodemographic factors at baseline are presented in Table 1. Most were between 25-35 years old (60.9%) and over half reported their ethnicity as non-White (52.2%). 80.4% indicated

			they were not in a relationship and many had previously been diagnosed with STIs (30.4% NSGI, 28.3% CT, 13.0% genital herpes or warts and 8.7% NG).
		(b) Indicate number of participants with missing data for each variable of interest	
		(c) *Cohort study*—Summarise follow-up time (eg, average and total amount)	
Outcome data	15*	*Cohort study*—Report numbers of outcome events or summary measures over time	
		Case-control study—Report numbers in each exposure category, or summary measures of exposure	
		Cross-sectional study—Report numbers of outcome events or summary measures	
Main results	16	(*a*) Give unadjusted estimates and, if applicable, confounder-adjusted estimates and their precision (eg, 95% confidence interval). Make clear which confounders were adjusted for and why they were included	See Table 2
		(*b*) Report category boundaries when continuous variables were categorized	
		(*c*) If relevant, consider translating estimates of relative risk into absolute risk for a meaningful time period	

Continued on next page

For peer review only

For peer review only - http://bmjopen.bmj.com/site/about/guidelines.xhtml

Other analyses	17	Report other analyses done—eg analyses of subgroups and interactions, and sensitivity analyses		
**Discussion**				
Key results	18	Summarise key results with reference to study objectives	7-9	In this small feasibility study we detected decreases in mean HRQoL and self-esteem scores in participants with a final diagnosis of CT or NSGI from the time of clinical presentation to approximately one week after receiving NAAT results. Although participants diagnosed with CT and NSGI showed greater negative impact in this time period, interestingly, participants who ultimately received a NAD diagnosis also experienced decreased self-esteem scores. Studies using qualitative methods and non-validated questionnaires have shown men diagnosed with CT experience negative psychosocial impacts [7, 8]. However, this is the first time that a negative impact of diagnosis of NSGI has been demonstrated to our knowledge.

For peer review only - http://bmjopen.bmj.com/site/about/guidelines.xhtml

				We experienced high LTFU that resulted in low numbers of respondents within diagnostic categories, which meant we were unable to adjust our analysis for common confounders, such as education and socioeconomic status, known to be associated with gonorrhoea [26,27] . This may be a source of bias in our results.<br>In interviews, participants with NSGI reported concerns regarding the uncertainty of the diagnosis, and fear of the impact of a possible STI diagnosis on their relationship. These findings suggest receipt of an STI or presumptive STI diagnosis in this population may have complex psychosocial impacts. These impacts may be multi-factorial and anxiety seems to be highly contextual and affected by relationship status and knowledge about STIs.
Limitations	19	Discuss limitations of the study, taking into account sources of potential bias or imprecision. Discuss both direction and magnitude of any potential bias	8-9	We experienced high LTFU that

For peer review only - http://bmjopen.bmj.com/site/about/guidelines.xhtml

resulted in low numbers of respondents within diagnostic categories, which meant we were unable to adjust our analysis for common confounders, such as education and socioeconomic status, known to be associated with gonorrhoea [26,27] . This may be a source of bias in our results.

Although the Rosenberg Self Esteem scale and the EQ-5D-5L scales we used in this study have been validated in men and women, the panel of questions used to assess men's' feelings about a diagnosis of CT or NSGI in the FU-1 CASI has not been validated in men, or for non-CT STI diagnoses. In addition, we assessed psychosocial and HRQoL impact at a single time-point, which does not measure durability of the observed decrease in self-esteem in this population, and data suggest impact of STI diagnoses may decrease over time [28].

For peer review only - http://bmjopen.bmj.com/site/about/guidelines.xhtml

Interpretation	20	Give a cautious overall interpretation of results considering objectives, limitations, multiplicity of analyses, results from similar studies, and other relevant evidence	9	In conclusion, our study shows that heterosexual men with urethral symptoms may experience negative psychosocial effects following SHC attendance. Given that incidence of NSGI is high [16] and up to 20% of men affected have recurrent symptoms [5, 6], a significant number of men may be adversely affected. Future research in this area is needed, firstly to address the high level of LTFU, and then to test the generalisability of our findings as to whether there are significant psychosocial harms of giving presumptive and non-specific diagnoses.
Generalisability	21	Discuss the generalisability (external validity) of the study results	9	Future research in this area is needed, firstly to address the high level of LTFU, and then to test the generalisability of our findings as to whether there are significant psychosocial harms of giving presumptive and non-specific diagnoses.
**Other information**				

For peer review only - http://bmjopen.bmj.com/site/about/guidelines.xhtml

Funding	22	Give the source of funding and the role of the funders for the present study and, if applicable, for the original study on which the present article is based	10	This study was funded by an Innovate UK (SBRI grant no. 971452) awarded to Atlas Genetics Ltd. Neither Atlas Genetics nor Innovate UK had a role in the design, collection, management, analysis, interpretation of data or decision to submit a report.

*Give information separately for cases and controls in case-control studies and, if applicable, for exposed and unexposed groups in cohort and cross-sectional studies.

Note: An Explanation and Elaboration article discusses each checklist item and gives methodological background and published examples of transparent reporting. The STROBE checklist is best used in conjunction with this article (freely available on the Web sites of PLoS Medicine at http://www.plosmedicine.org/, Annals of Internal Medicine at http://www.annals.org/, and Epidemiology at http://www.epidem.com/). Information on the STROBE Initiative is available at www.strobe-statement.org.

For peer review only

For peer review only - http://bmjopen.bmj.com/site/about/guidelines.xhtml

BMJ Open

Health related quality of life and psychosocial impacts of a diagnosis of non-specific genital infection in symptomatic heterosexual men attending UK Sexual Health clinics: a feasibility study.

Journal:	*BMJ Open*
Manuscript ID	bmjopen-2017-018213.R1
Article Type:	Research
Date Submitted by the Author:	17-Oct-2017
Complete List of Authors:	Hill-Tout, Rachel; St George's NHS Foundation Trust, Genitourinary Medicine & HIV<br>Harding- Esch, Emma; Public Health England, HIV & STI; St George's University of London, Applied Diagnostic Research and Evaluation Unit, Institute for Infection & Immunity<br>Pacho, Agata; St George's University of London, Applied Diagnostic Research and Evaluation Unit, Institute for Infection & Immunity<br>Furegato, Martina; Public Health England, HIV & STI<br>Fuller, Sebastian; St Georges University of London, Applied Diagnostic Research and Evaluation Unit, Institute for Infection & Immunity<br>Sadiq, S. Tariq; St George's University of London, Applied Diagnostic Research and Evaluation Unit, Institute for Infection and Immunity Research Institute
**Primary Subject Heading**:	Sexual health
Secondary Subject Heading:	Patient-centred medicine, Qualitative research, Sociology, Sexual health
Keywords:	GENITOURINARY MEDICINE, QUALITATIVE RESEARCH, Genitourinary medicine < INTERNAL MEDICINE

SCHOLARONE™
Manuscripts

Health related quality of life and psychosocial impacts of a diagnosis of non-specific genital infection in symptomatic heterosexual men attending UK Sexual Health clinics: a feasibility study.

Rachel Hill-Tout[1], Emma M. Harding-Esch[2, 3], Agata Pacho[2], Martina Furegato[3], Sebastian S. Fuller*[†2], S Tariq Sadiq[†1, 2]

* Corresponding author

[†] Joint senior authors

1. St Georges University Hospitals NHS Foundation Trust
   Blackshaw Rd, London SW17 0QT UK

2. Applied Diagnostic Research and Evaluation Unit
   Institute for Infection & Immunity
   St George's, University of London
   London, SW17 0RE, UK

3. Public Health England
   61 Colindale Avenue
   London, NW9 5EQ UK

Rachel Hill-Tout Specialist Registrar HIV/GUM
orcid.org/0000-0003-1892-8598
r.hill-tout@nhs.net

Emma M. Harding-Esch
ADREU Epidemiology Lead & Programme Manager
orcid.org/0000-0002-1432-8109

Agata Pacho
ADREU Research Assistant
orcid.org/0000-0002-0653-5095

Martina Furegato
Statistician - HIV & STI department

Sebastian S. Fuller
ADREU Social Science Lead
orcid.org/0000-0002-9284-6630
sfuller@sgul.ac.uk

S Tariq Sadiq
Reader in HIV and Sexual Health Medicine, Consultant Physician / ADREU Lead
orcid.org/0000-0002-0608-381

ABSTRACT

Introduction:

Non-specific genital infection (NSGI; non-*Chlamydia trachomatis* (CT), non-*Neisseria gonorrhoeae* (NG) associated urethritis) is a common diagnosis in symptomatic heterosexual men attending UK Sexual Health Clinics (SHCs) but little is known about the psychosocial impact of this diagnosis.

For peer review only

Methods:

We conducted an observational study among symptomatic heterosexual men attending SHCs to evaluate the psychosocial impact of an NSGI diagnosis compared to a diagnosis of CT, NG or no abnormalities detected (NAD) focussing on the feasibility of our study methodology. Participants completed a Computer Assisted Self Interview (CASI) including validated health-related quality-of-life (HRQoL) and self-esteem measures before diagnostic testing and two weeks after receiving test results, and a qualitative interview. We compared scores between diagnostic groups using paired t-tests, qualitative data were analysed thematically, and feasibility was assessed by process analysis.

Results:

60 men completed the baseline CASI (75% response rate). 46 (76.6%) were eligible for follow-up; of these, 11 (23.9%) completed the follow-up CASI, and 3 (27.3%) completed the qualitative interview. 81.7% of participants left CASI feedback at baseline: 73.5% reported the questionnaire as 'fine' or 'very good'. Qualitative interview participants reported the study was acceptable. All patients completing FU-1 showed measurable decreases from baseline to FU-1 in self-esteem (CT p=0.009; NSGI p=0.008; NAD p=0.057). Measurable decreases in HRQoL from baseline to FU-1 were found among participants with CT (p=0.002) or NSGI (p=0.001). We did not find differences in baseline sociodemographic factors between participants who completed FU-1 and those that did not.

Conclusions:

Although most participants indicated study acceptability at baseline, and we employed measures to increase retention (CASI questionnaires, reminder messages, a focus on men's health), attrition was high. We found that heterosexual men attending SHCs with symptoms of urethritis may experience negative psychosocial impacts regardless of their diagnosis, which warrants further investigation. We recommend exploring novel strategies to decrease attrition in future studies.

Article Summary

Strengths and Limitations of this study

- This is the first data to our knowledge investigating the psychosocial impact of non-specific genital infection, a very common diagnosis in heterosexual men attending sexual health clinics.
- This study used both validated scales and qualitative interviews to assess psychosocial impact.
- We used computer assisted self-interviewing to collect data on psychosocial impact as this has been shown to improve data quality in sensitive topics.
- The study was small and was conducted in two London sexual health clinics which may limit the generalisability of our results.
- There was high loss to follow-up after baseline interviews which may have biased our results.

For peer review only - http://bmjopen.bmj.com/site/about/guidelines.xhtml

INTRODUCTION

Non-specific Genital Infection (NSGI) or, non-*Chlamydia trachomatis* (CT), non-*Neisseria gonorrhoeae* (NG) associated urethritis (NGU), is the third most frequently diagnosed condition in heterosexual men attending sexual health clinics (SHCs) in the United Kingdom (UK), with 30,381 cases/year compared to CT (42,483/year) and first presentation of genital warts (32,656/year) [1]. NSGI is a diagnosis of exclusion; men with urethral symptoms are screened with urethral microscopy in SHCs, and if this demonstrates urethritis with no gonococci, a provisional diagnosis of NGU is made [2]. If urinary Nucleic Acid Amplification Tests (NAATs) sent for NG and CT are both negative, the diagnosis is recorded as NSGI.

Although a common condition, NSGI remains poorly understood. NSGI can be caused by a variety of sexually transmitted infections (STIs), including *Mycoplasma genitalium* (MG) and *Trichomonas vaginalis* (TV), which are not routinely tested for in SHCs, and many cases have an uncertain aetiology [2]. Microscopy, on which the diagnosis rests, has poor specificity for known causes of urethritis [3] and is subject to considerable observer variation [4].

National guidelines recommend that men diagnosed with NGU are informed they may have an STI, given same day empirical antibiotics, advised their partners should attend for STI testing and empirical treatment, and to abstain from sex until treatment is completed [2]. Men receive CT/NG NAAT results usually within 8 days [5]. Following first-line antibiotics for NSGI, 10%–20% of men return because of persistent symptoms [6,7].

Men who undergo STI testing, and are diagnosed with an STI report negative impacts on their psychosocial wellbeing, including feelings of stigma [8–10], however, to our knowledge, there are no published data on the psychosocial impact of NSGI diagnosis among men. Stigma has been shown in men to be associated with a delay in testing for STIs, and decreased willingness to notify their casual partners of their STI diagnosis [11]. Studies on psychosocial impact, partner notification and quality of life impact have predominantly been carried out in women [9,12–14], with few studies recruiting men. A greater understanding of the degree and nature of psychosocial impacts of a diagnosis of an STI, or a 'presumed STI' such as NSGI, in men, is important to optimise interventions, including partner notification and risk-behaviour modification, which aim to reduce the negative impact of STIs, such as complications of infection.

Men are reportedly poor users of health services, largely due to culturally dominant ideals of masculinity that equate illness with weakness, which may impede care-seeking [15–18]. In sexual healthcare, there has traditionally been a focus on female reproductive health [19], and this may further discourage male attendance [19,20]. However, men are willing to respond openly to questions about their sexual health, particularly when enquiries are focused specifically on men's needs [19,21].

We decided to focus on heterosexual men in this study as there are important differences between men who have sex with men (MSM) and men who have sex exclusively with women (MSEW) in their access of SHC

For peer review only - http://bmjopen.bmj.com/site/about/guidelines.xhtml

services, reported sexual health risks, their own perception of risk of STIs, and sexual health and well being outcomes, suggesting that their experience of diagnosis of a genital infection may also be different. The Natsal-3 study reported that in comparison with MSEW, MSM are more likely to report higher numbers of partners, higher rates of condomless sex, to perceive themselves at higher rates of STIs, a greater likelihood to attend SHCs, and higher rates of depression, substance use and non-volitional sex [22]. Due to these important differences between MSM and MSEW, we decided that psychosocial impacts of an STI diagnosis in MSM merited studying separately.

Because of a lack of models to measure the psychosocial impact of an NSGI diagnosis among men, and the potential difficulty in recruiting and retaining this group in sexual health studies, we designed a small-scale longitudinal mixed-method feasibility study to inform on our research methods and better understand the psychosocial and health related quality of life (HRQoL) status of symptomatic heterosexual men before and after receiving a diagnosis of NSGI, CT, NG or nothing abnormal detected (NAD).

METHODS

Study Design

We conducted an observational, longitudinal study in symptomatic heterosexual men using patient completed questionnaires assessing HRQoL and psychosocial status before and after receiving STI test results, and individual qualitative interviews to evaluate experiences at the SHC and acceptability of study methods.

Eligibility criteria were: heterosexual men presenting with urethral symptoms (dysuria, discharge or urethral discomfort), aged ≥16 years at either of two participating London SHCs between September - October 2016. We excluded men who were asymptomatic, not available for follow-up, and who were unable to understand English.

Following informed consent, participants were invited to complete a baseline computer assisted self-interview (CASI) assessing psychosocial and HRQoL status in clinic prior to consultation, a follow-up CASI after receipt of all test results 1-2 weeks after baseline (Follow-up 1 (FU-1)) and a qualitative interview 2-4 weeks after baseline (Follow-up 2 (FU-2)).

The participant pathway is detailed in Figure 1. After completion of the baseline CASI, any participants diagnosed with an STI other than CT, NG or NSGI, or who did not have both urethral microscopy and urinary NAATs, were withdrawn from the study. Once participants were notified of their NAAT CT/NG results by the SHC (phone call/ text message), the research team texted participants an electronic link to the FU-1 CASI.

CASIs were optimised for web-enabled devices including smartphones. We offered participants the choice to complete CASIs using their own device or a study tablet-device and use of a private room in clinic. Study staff sent ≤2 text message reminders for each follow-up. Participants who did not complete FU-1 were considered ineligible for FU-2. Participants received gift voucher reimbursements: £5 (baseline), £10 (FU-1), £20 (FU-2).

For peer review only - http://bmjopen.bmj.com/site/about/guidelines.xhtml

We categorised participants into four diagnostic groups based on microscopy and NAAT results: NSGI: NGU diagnosis on urethral microscopy and CT/NG NAATs negative; NG: NG on urethral microscopy and/or positive NAAT; CT: positive NAAT; NAD: no urethritis on microscopy and CT/NG NAATs negative.

Questionnaire design

Baseline and follow-up CASIs included validated scales measuring psychosocial status and HRQoL and additional questions. The EQ-5D-5L scale [23] as a measure of HRQoL and the Rosenberg Self-Esteem scale [24] as a measure of potential stigmatising effects of screening for STIs, were used in both baseline and FU-1 CASIs.

In addition to the above, the baseline CASI included the Multidimensional Scale of Social Support [25] to assess coping resources and questions on sociodemographic and current relationship status, and the FU-1 CASI included a question on current relationship status and a panel of questions previously developed to assess impact of CT diagnosis in women [13], which we modified for use in men by removing a pregnancy-related question. Participants were asked to indicate acceptability of the CASIs (free-text response) at baseline and FU-1. (Supplementary Appendicies 1., 2. and 3.)

Sample size

As this was a feasibility study with no precedent, no formal sample size could be calculated. We planned to approach 100 patients at baseline as a convenience target, and assuming approximately 50% refusal, we expected 50-60 patients to be enrolled and estimated ~50% attrition at each follow-up.

Outcome measures

Primary outcome: Qualitative and quantitative process data on recruitment and retention rates at baseline and follow-up to inform on feasibility of study design. Evaluation of potential relevance of questions validated in other populations for men diagnosed with NSGI.

Secondary outcomes:

Percentage change from baseline to FU-1 in mean EQ-5D-5L and Rosenberg Self-Esteem Scale in men diagnosed with CT, NG, NSGI or NAD. We did not have prior expectations about different impacts of these diagnoses, and as there are no studies comparing the impact of CT to NG or NSGI in men, this was exploratory. We were interested in investigating whether NSGI was perceived as a benign diagnosis or whether it elicited negative psychosocial impacts which have been reported in men diagnosed with STIs such as CT [10].

Data analysis

Questionnaire data:

Scores for psychosocial and HRQoL scales were calculated according to published methods [24][23][25]. For all eligible men, mean and 95% Confidence Intervals of baseline scores for EQ-5D-5L, Rosenberg Self-Esteem and

For peer review only - http://bmjopen.bmj.com/site/about/guidelines.xhtml

Multidimensional Social Support Scales were compared in men diagnosed with CT, NG, NSGI and NAD, using paired t-tests. To enable us to compare the EQ-5D-5L score across the different diagnoses (CT, NG, NSGI and NAD), a mean score was calculated: we calculated mean values for each of the five dimensions of the EQ-5D-5L, and a final score for the EQ-5D-5L was created using the sum of the mean value for each dimension. We calculated the mean value rather than an index score for the EQ-5D-5L to allow comparison between the outcomes of EQ-5D-5L with the Rosenberg Self-Esteem and Multidimensional Social Support Scales.

To test for non-response bias, we compared sociodemographic data of those who completed both baseline and follow-up to those who were lost to follow-up (LTFU) using Pearson's Chi^2 tests and Kruskal-Wallis tests. Paired t-tests were used to assess differences in mean score for EQ-5D-5L and Rosenberg Self-Esteem Scales between baseline and follow-up in men diagnosed with CT, NG, NSGI and NAD. We used the students t-test distribution to calculate p-values. Statistical significance was set at $p<0.05$. Statistical analysis was carried out using STATA version 13.1 (StataCorp, 2015).

Qualitative data: Audio recordings were transcribed and transcripts checked for accuracy against the recording. Cleaned transcripts were then imported into NVivo (Version 10, 2012) for analysis. Two researchers assigned themes to text independently, with discrepancies resolved by consensus before final themes reported. All names of participants presented here are pseudonyms.

RESULTS

We approached 80 patients, of whom 60 (75%) enrolled and completed the baseline CASI. 46 (76.6%) were eligible for follow-up; of these, 11 (23.9%) completed the FU-1 CASI, and 3 of these (27.3%) completed FU-2 (Figure 2.)

Final diagnoses among all eligible participants (n=46) were: CT, 7 (15.2%); NG, 2 (4.3%); NSGI, 23 (50%); NAD, 14 (30.4%), and among participants who completed FU-1 (n=11) were CT, 4 (36.4%); NSGI, 4 (36.4%); NAD, 3 (27.3%). None of the participants diagnosed with NG completed FU-1. Two participants with NSGI and one with NAD completed FU-2. Clinical records indicated that all participants were notified of their CT/NG NAAT diagnosis within 7 days of completing baseline.

Men who reported urethral symptoms but had no abnormalities on urethral sample testing and negative CT and NG results (categorised as NAD) had a variety of non-STI causes for their symptoms diagnosed, including irritant or *Candida* balanitis, lower urinary tract symptoms which were likely prostatic in origin, non-infectious genital pain syndromes, and symptoms secondary to urethral trauma.

Participants' sociodemographic factors at baseline are presented in Table 1. Most were between 25-35 years old (60.9%), and over half reported their ethnicity as non-White (52.2%). 80.4% indicated they were not in a

For peer review only - http://bmjopen.bmj.com/site/about/guidelines.xhtml

relationship and many had previously been diagnosed with STIs (30.4% NGU, 28.3% CT, 13.0% genital herpes or warts and 8.7% NG).

Pearson's Chi^2 tests and Kruskal-Wallis test results for non-response bias were not statistically significant so we assumed data were missing at random. We compared participants who completed FU-1 to those LTFU by employment, age, education, ethnicity, and current relationship status; none of these comparisons showed significant differences.

Process evaluation

All patients who completed the baseline CASI did so using the Study tablet device in a private room in the clinic. Participants completed the baseline CASI in about 5 minutes. All participants completed the follow-up CASI at home on a participant-owned device.

CASI feedback suggested good acceptability: of the total 60 who completed the Baseline CASI, 81.7% left feedback, of whom 73.5% reported that the questionnaire was "fine" or "very good" in a free-text box at the end of the questionnaire.

Participants suggested improvements to the study during qualitative interviews: to use the research as a tool for health promotion and to make CASI questions more clearly relevant to sexual health. Insight into LTFU was provided by one participant, who said he might not have continued with the study if he had received a positive STI diagnosis: "*I reckon if my tests had not come back the way I wanted them to, I probably wouldn't have done this last [follow-up]. … I might've still said, 'Yes, let's do it.' I don't know but I… Just sitting here, thinking about it now, I probably wouldn't complete the rest of it."* (Lucas, NAD diagnosis).

CASI analysis

Distributions of participants' psychosocial scores at baseline by final diagnosis are shown in Table 2. At baseline participants ultimately diagnosed with NG had significantly lower ($p \leq 0.050$) mean Multidimensional Scale of Social Support scores (indicating lower social support), compared to men ultimately diagnosed with NSGI or NAD, as well as lower scores as compared to those diagnosed with CT (p=0.059). Participants with NG also had lower mean Rosenberg Self-esteem scores (indicating lower self-esteem) at baseline as compared to those with NSGI (p=0.038). We did not find any other baseline differences between diagnosis groups.

We found men with NSGI and CT showed a significant decrease in mean EQ-5D-5L score between baseline and FU-1(-39%, p=0.001 and -44%, p=0.002) indicating decreased HRQoL. There were also decreases in self-esteem from baseline to FU-1 seen in all participants, as measured by mean Rosenberg Self-Esteem scores (NSGI=-25%, p=0.008; CT=-29%, p=0.009; NAD=-21%, p=0.057).

For peer review only - http://bmjopen.bmj.com/site/about/guidelines.xhtml

Participants who reported that they had been diagnosed with an infection (n=5, 4 with a final diagnosis of CT and 1 with NSGI) completed a panel of questions in the FU-1 CASI to assess their feelings about their diagnosis. All participants reported that they were 'not worried as it is curable' and did not think the diagnosis would change how their friends thought about them, but only one participant (diagnosed with CT) reported he had talked to his friends about his diagnosis. All participants reported concern that they might get the infection again, and the majority of those with a CT diagnosis (75%) reported concern about exposure to other STIs, feeling "dirty" and embarrassed, being scared to tell their partners about their diagnosis and worried that their partners thought they had been unfaithful.

Qualitative analysis

In interviews, participants compared different STIs as a way to contextualise their level of concern about their own diagnosis. For one participant, his main concern was the possibility of being diagnosed with HIV: *"Look, to be honest with you, the key one everyone really wants to know about is HIV … all the other ones are treatable quite easily…"* (Lucas, NAD diagnosis). There were several points within the interviews where participants expressed concern at being diagnosed with an infection they did not know about, indicating that non-specific diagnoses may cause negative impact: *"…to be honest, I'm a little bit worried about this because there is no name of the new bacteria, so we don't know if it's dangerous or not..."* (Liam, NSGI diagnosis).

Among the two interview participants diagnosed with NSGI, one described anxiety following his presumptive CT diagnosis in clinic. He was in a long-term monogamous relationship and so was not expecting an STI diagnosis: *"My heart's racing. I'm thinking, 'but how, I can't.' … I just had a lot of thoughts in my head. … And I told [the doctor], 'I can't. I don't know how I would have this.' He's saying, 'Well, whatever you have, it's sexually transmitted.' I said, 'But it can't be.' So of course I start getting things in my head."* (Jacob, NSGI diagnosis). (Supplementary Appendix 4.)

DISCUSSION

In this small observational feasibility study we detected decreases in mean HRQoL and self-esteem scores in participants with a final diagnosis of CT or NSGI from the time of clinical presentation to approximately one week after receiving NAAT results. Although participants diagnosed with CT and NSGI showed greater negative impact in this time period, interestingly, participants who ultimately received a NAD diagnosis also experienced decreased self-esteem scores. Studies using qualitative methods and non-validated questionnaires have shown men diagnosed with CT experience negative psychosocial impacts [7, 8]. However, this is the first time that a negative impact of diagnosis of NSGI has been demonstrated to our knowledge. In interviews, participants with NSGI reported concerns regarding the uncertainty of the diagnosis, and fear of the impact of a possible STI diagnosis on their relationship. These findings suggest receipt of an STI or presumptive STI diagnosis in this population may have complex psychosocial impacts. These impacts may be multi-factorial and anxiety seems to be highly contextual and affected by relationship status and knowledge about STIs.

For peer review only - http://bmjopen.bmj.com/site/about/guidelines.xhtml

Despite concerns that the target group of heterosexual men attending SHCs would be hard to recruit [26], we found 75% of men were happy to enrol in this study. This should encourage researchers to investigate views from this group regarding STI diagnosis and screening. A study investigating the psychosocial experiences of patients attending a clinic, which recruited men and women diagnosed with an STI from an SHC, reported a recruitment rate of 33% [27]. This study involved ten interviews lasting between 20-45 minutes each. Our higher baseline recruitment rate may be due to the use of CASI interviewing, fewer interviews, or because patients were recruited before they had any diagnosis made.

One of the difficulties associated with conducting longitudinal studies is attrition. In our study, of those eligible for follow-up, 24 % overall completed the follow-up CASI, 25% of those with a diagnosis of NSGI, CT or NG, and 21% of those with NAD. A longitudinal questionnaire study investigating community screening for CT reported follow-up rates of over 60% , however, eligibility for follow-up excluded those with positive STI test results, and 60% of the sample was female, reflecting the higher response rate amongst women [12]. Our study's qualitative component had a follow-up rate of 27% overall, 25% in those who had any positive diagnosis, and 33% in those with NAD. By comparison, a study including men, investigating patient views on CT screening recruited 6.6% of those who completed the baseline questionnaire to a qualitative interview, which was below their target of 10% [28].

We embedded several mechanisms in our study to improve participant acceptability and retention. Privacy when completing sensitive questions has been shown to improve participant acceptability for revealing potentially stigmatising information [29]; we used a web-based CASI and the option for participants to self-complete the questionnaire in a private room in the clinic. Potential research participants, and men specifically, are more likely to participate in research that represents their concerns [19,21,30]; eligibility for this study was restricted to those patients who were likely to be diagnosed with an STI, and focused on the impact of those diagnoses. In addition, reimbursements have been shown to play a part in increasing enrolment and retention in research [30,31]; we reimbursed participants in increasing amounts to reflect the time and effort we estimated were necessary to complete the research. Despite these mechanisms, and positive participant feedback on the baseline CASI and in our qualitative interviews, we experienced high LTFU. High LTFU resulted in low numbers of respondents within diagnostic categories, which meant we were unable to adjust our analysis for common confounders, such as education and socioeconomic status, known to be associated with gonorrhoea [32,33]. This may be a source of bias in our results. We did not find significant differences between groups in terms of non-response bias and other characteristics, however this is not surprising as the power to detect differences between groups is greatly affected by sample size and our study included a small number of participants. Recruiting larger numbers of participants in future studies will increase the statistical power to detect any important differences between groups.

For peer review only - http://bmjopen.bmj.com/site/about/guidelines.xhtml

We explored potential correlates and causes for LTFU within our dataset. Men's socioeconomic status and age has been seen to affect both their knowledge of and interaction with health services [20]. However, we did not find significant differences in these factors between those LTFU and those who completed FU-1. One participant indicated in qualitative interview that if he had received a positive STI diagnosis it was unlikely that he would have agreed to continue with the research. This may give insight into why no patients receiving a positive CT or NG diagnosis agreed to FU-2.

We had planned to further explore study acceptability by inviting participants who failed to complete FU-1 to qualitative interviews; however, we followed Research Ethics Committee recommendations to consider that these participants had passively withdrawn from the study and refrained from inviting them to FU-2. Yet, qualitative and quantitative methods of data collection are viewed differently by respondents [34,35]. One participant described the difference between his experiences completing the survey and participating in an interview:

"…when I'm sitting here with you, obviously it's just me and you and you've got my hundred percent full attention, haven't you? But then when I'm sitting at my desk at work, trying to complete a survey and there's other things going on and people asking me questions and 'can you do this, can do you that'; it's different." (Lucas, NAD diagnosis)

Although we cannot know if participants who did not complete FU-1 would have participated in a qualitative interview if they had been invited, one participant left feedback on the baseline CASI indicating he would have preferred to elaborate on his answers, and several participants informally conveyed this to the researcher at baseline. Research has shown that one unique benefit of qualitative interviews is that it provides participants with an opportunity to expand or contextualise issues [34,35] . In addition, the insights we gained from the limited qualitative interviews we conducted indicate the potential usefulness of this method to understand nuances of acceptability that may lead to reduced LTFU. We therefore recommend that future feasibility studies include qualitative components as a means to allow participants, including those who fail to complete survey questionnaires, to provide important feedback on study methods. Future studies could also consider offering a qualitative interview at the point of recruitment, not only at follow-up, to widen the possibility of a qualitative exploration among participants who do not find the CASI questionnaire acceptable.

A weakness of our study is that we did not include asymptomatic men, hence we cannot exclude the possibility that the negative psychosocial impacts observed with men diagnosed with CT or NSGI were not related to the confounding factors of being symptomatic or the attendance at an SHC (including undergoing genital examination, and a urethral swab). Few studies have examined the impact of an SHC clinic visit on psychosocial and/or self-esteem outcomes for men, but those that have been conducted did report negative impacts; studies among heterosexual men attending SHCs who are diagnosed with an STI reported stigma, anxiety, isolation, concerns regarding relationships, feeling 'contaminated' and fear of exposure, as well as reluctance

For peer review only - http://bmjopen.bmj.com/site/about/guidelines.xhtml

to attend an SHC clinic for help [27,36]. A focus group of young male adolescents reported that accessing SHCs was stressful due to fear of stigma and a loss of social status, shame, and embarrassment, a lack of privacy/confidentiality, and challenges in accessing and negotiating the healthcare system [36]. Shoveller et al reported that factors involved in STI testing, such as urethral swab testing, were seen by men as fearful, and as a process where men felt very vulnerable; most of these male participants reported feeling anxious waiting for potentially bad news in addition to feeling that their masculinity had been compromised through an admission of needing help [37]. Limitations of these studies are that they did not specify whether patients had symptoms, or if men had experienced (invasive or non-invasive) testing procedures, and tended to recruit patients who had already been diagnosed with an STI, which makes the impact of testing and receiving a diagnosis difficult to separate [10,27,36,37]. Men who used postal screening for CT and had negative test results were not found to experience a negative impact on self-esteem or anxiety, suggesting that STI testing at home may reduce negative impacts associated with visiting an SHC, however this study did not follow up men with positive CT diagnoses [12]. Future studies should consider including asymptomatic men alongside symptomatic men, to better evaluate the impact of attending clinic, and the experience of having genital examination and invasive testing or not, on psychosocial wellbeing and self-esteem.

The issue of gender concordance between participants and interviewers/researchers has been discussed in the literature [38] and the fact that our study recruiter was female may have affected recruitment and retention. A study investigating attitudes to Viagra in heterosexual Hispanic men, using telephone qualitative interviews, found that changing from female to male recruiters and data collectors increased their recruitment [26]. Participants in our study were offered the option of a male or female interviewer but may not have felt comfortable with asking for a particular interviewer, and so this may not have been sufficient to reduce the potential impact of gender discordance on acceptance of the invitation to an interview in our study. We suggest future studies involving qualitative interviews should consider the gender of recruiters and interviewers.

We received feedback from our participants that some of the wording of questions on our CASIs did not appear to be directly relevant to our study aims. Future studies should ensure that data collection measures use terms that are clear and familiar to participants, possibly through development of questionnaires with focus groups. Some men in our study also saw participation as an opportunity to learn more about their own health and requested that future studies offer research participants material on health promotion, i.e. material on the infection they were diagnosed with and how to reduce risk of infection.

Although the Rosenberg Self Esteem scale and the EQ-5D-5L scales we used in this study have been validated in men and women, the panel of questions used to assess men's feelings about a diagnosis of CT or NSGI in the FU-1 CASI has not been validated in men, or for non-CT STI diagnoses. In addition, we assessed psychosocial and HRQoL impact at a single time-point, which does not measure durability of the observed decrease in self-esteem in this population, and data suggest impact of STI diagnoses may decrease over time [39].

For peer review only - http://bmjopen.bmj.com/site/about/guidelines.xhtml

In conclusion, our study shows that heterosexual men with urethral symptoms may experience negative psychosocial effects following SHC attendance. Given that incidence of NSGI is high [16] and up to 20% of men affected have recurrent symptoms [5, 6], a significant number of men may be adversely affected. Future research in this area is needed, firstly to address the high level of LTFU, and then to test the generalisability of our findings as to whether there are significant psychosocial harms of giving presumptive and non-specific diagnoses.

TABLES AND FIGURES

Figure 1. Patient Pathway
Figure 2. Enrolment and Follow-up Chart
Table 1. Sociodemographic characteristics of enrolled participants at Baseline by final diagnosis.
Table 2. (a, b) Distribution of psychosocial scores by final diagnosis.

Supplementary Appendices

Appendix 1. Baseline CASI Questionnaire
Appendix 2. Follow-up 1 CASI Questionnaire
Appendix 3. Individual in-depth Interview Topic Guide
Appendix 4. Full list of themes for Qualitative Interview (Follow-up 2)

Table 1. <u>Sociodemographic characteristics of enrolled participants at Baseline by final diagnosis</u>

	CT n (%)	NG n (%)	NSGI n (%)	NAD n (%)	Total n
**Age**					
<24	5 (71.43%)	1 (50%)	5 (21.74%)	3 (21.43%)	14
25-35	2 (28.57%)	1 (50%)	17 (73.91%)	8 (57.14%)	28
>35	0 (0%)	0 (0%)	1 (4.35%)	3 (21.43%)	4
**Ethnicity**					
White	5 (71.43%)	0 (0%)	10 (43.48%)	6 (42.86%)	21
Asian	0 (0%)	1 (50%)	2 (8.7%)	2 (14.29%)	5
Black	1 (14.29%)	1 (50%)	10 (43.48%)	2 (14.29%)	14
Other	1 (14.29%)	0 (0%)	1 (4.35%)	3 (21.43%)	5
Prefer not to say	0 (0%)	0 (0%)	0 (0%)	1 (7.14%)	1
**Age at leaving full-time education**					
16 years or less	0 (0%)	0 (0%)	1 (4.35%)	3 (21.43%)	4
17 or 18 years	0 (0%)	0 (0%)	6 (26.09%)	0 (0%)	6
19 years or over	4 (57.14%)	0 (0%)	14 (60.87%)	10 (71.43%)	28
Still in full-time education	3 (42.86%)	2 (100%)	2 (8.7%)	1 (7.14%)	8
**Highest educational achievement**					
No qualifications/No formal qualifications	1 (14.29%)	0 (0%)	1 (4.35%)	0 (0%)	2
1-5 GCSEs	0 (0%)	0 (0%)	3 (13.04%)	2 (14.29%)	5

For peer review only - http://bmjopen.bmj.com/site/about/guidelines.xhtml

2 or more A-levels or equivalent	1 (14.29%)	1 (50%)	4 (17.39%)	2 (14.29%)	8
Bachelor's degree or equivalent and higher	5 (71.43%)	1 (50%)	13 (56.52%)	8 (57.14%)	27
Other	0 (0%)	0 (0%)	2 (8.7%)	2 (14.29%)	4
**Do you have any long-standing condition?**					
Yes	1 (14.29%)	0 (0%)	1 (4.35%)	2 (14.29%)	4
No	6 (85.71%)	2 (100%)	22 (95.65%)	12 (85.71%)	42
**Previously diagnosed with CT?**					
Yes	4 (57.14%)	1 (50%)	7 (30.43%)	1 (7.14%)	13
No	3 (42.86%)	1 (50%)	16 (69.57%)	13 (92.86%)	33
**Previously diagnosed with NG?**					
Yes	0 (0%)	2 (100%)	2 (8.7%)	0 (0%)	4
No	7 (100%)	0 (0%)	21 (91.3%)	14 (100%)	42
**Previously diagnosed with NGU?**					
Yes	2 (28.57%)	0 (0%)	9 (39.13%)	3 (21.43%)	14
No	5 (71.43%)	2 (100%)	14 (60.87%)	11 (78.57%)	32
**Previously diagnosed with herpes/warts?**					
Yes	3 (42.86%)	0 (0%)	1 (4.35%)	2 (14.29%)	6
No	4 (57.14%)	2 (100%)	22 (95.65%)	12 (85.71%)	40
**Relationship status**					
Married and living with wife	0 (0%)	0 (0%)	1 (4.35%)	2 (14.29%)	3
Cohabiting but not married	1 (14.29%)	0 (0%)	2 (8.7%)	2 (14.29%)	5
Separated, divorced, or widowed	0 (0%)	0 (0%)	1 (4.35%)	0 (0%)	1
Single (that is never married)	6 (85.71%)	2 (100%)	19 (82.61%)	9 (64.29%)	36
Prefer not to say	0 (0%)	0 (0%)	0 (0%)	1 (7.14%)	1
**Break up in the last 30 days**					
Yes	1 (14.29%)	0 (0%)	3 (13.04%)	5 (35.71%)	9
No	6 (85.71%)	2 (100%)	20 (86.96%)	9 (64.29%)	37

Table 2 Distribution of psychosocial scores by final diagnosis

Tab 2.a Distribution of psychosocial scores by final diagnosis within the baseline group and results from students t-test

		**Mean**	**95% CI**	**p-value NSGI vs. CT**	**p-value NSGI vs. NAD**	**p-value CT vs. NAD**	**p-value NG vs. CT**	**p-value NG vs. NSGI**	**p-value NG vs. NAD**
**EQ-ED-5L**	**NG**	2.2	1.4-3.0	0.255	0.227	0.999	0.932	0.430	0.940
	**NSGI**	2.0	1.9-2.1						
	**CT**	2.2	1.9-2.5						
	**NAD**	2.2	1.9-2.4						
**Rosenberg Self**	**NG**	2.1	1.2-3.0	0.819	0.582	0.835	**0.068**	**0.038**	0.122

For peer review only - http://bmjopen.bmj.com/site/about/guidelines.xhtml

**Esteem Scale**	**NSGI**	3.2	3.0-3.4						
	**CT**	3.3	2.9-3.7						
	**NAD**	3.3	3.0-3.6						
**Multidimensional scale of social support**	**NG**	2.5	2.0-3.0	0.583	0.610	0.918	**0.059**	**0.016**	**0.040**
	**NSGI**	3.1	2.9-3.2						
	**CT**	3.2	2.8-3.6						
	**NAD**	3.2	2.9-3.4						

Tab 2.b Distribution of psychosocial scores by final diagnosis within the FU group and within the Baseline group restricted to those with a FU record comparing Baseline and FU scores

		**Baseline***		**FU-1**			
		**Mean**	**95% CI**	**Mean**	**95% CI**	**% difference of mean Baseline* and FU-1**	**p-value Baseline* vs. FU- 1**
**EQ-ED-5L**	**NSGI**	1.8	1.8-1.8	1.1	0.9-1.3	-39%	**0.001**
	**CT**	2.3	1.7-3.0	1.3	1.0-1.5	-44%	**0.002**
	**NAD**	1.9	0.9-3.0	1.6	0.4-2.8	-16%	0.633
**Rosenberg Self Esteem Scale**	**NSGI**	3.2	2.7-3.7	2.4	2.2-2.6	-25%	**0.008**
	**CT**	3.1	2.6-3.6	2.2	2.0-2.4	-29%	**0.009**
	**NAD**	3.3	2.6-4.0	2.6	2.4-2.8	-21%	**0.057**

*restricted to patients with a FU record

<u>Abbreviations used in Tables 2 a and b</u>

CT- *Chlamydia trachomatis*
NG- *Neisseria gonorrhoea*
NSGI- Non specific genital infection
NAD- No abnormality detected
FU- Follow-up

Ethical Approval

This study was approved by the London Bridge Research Ethics Committee (REC reference 16/LO/0955).

Acknowledgements

Thank you to the participants who took part in the study, our collaborators and the steering committee for their advice, including: Atlas Genetics, who were awarded the Innovate UK SBRI grant; Aquarius Population Health; Scientific Steering Committee (Chris Price, Kate Folkard, David Livermore), and the clinical leads at participating clinics (Richard Lau, Paul Lister).

Contributor Statement

STS conceived of the Study. RHT, SSF and EHE initiated the study design. AP, SSF, RHT and EHE implemented the study. MF and EHE provided statistical oversight. RHT wrote the initial draft of the manuscript. All authors contributed to and approved the final manuscript.

Competing Interests

The Applied Diagnostic Research and Evaluation Unit at St George's, University of London (STS, EHE, SF, AP), receives funding from the National Institute of Health Research (NIHR) i4i Programme (grant number II-LB-

For peer review only - http://bmjopen.bmj.com/site/about/guidelines.xhtml

0214-20005), Atlas Genetics, Alere, Hologic Cepheid, SpeeDx, Sekisui and Becton Dickinson to develop Point of Care Tests for STIs. SF and EHE are members of Becton Dickenson's Advisory Board for the provision of sexual health in the UK. MF and RHT have no competing interests. The views expressed are those of the authors and not necessarily those of the NIHR, the NHS or the Department of Health.

Funding

This study was funded by an Innovate UK (SBRI grant no. 971452) awarded to Atlas Genetics Ltd. Neither Atlas Genetics nor Innovate UK had a role in the design, collection, management, analysis, interpretation of data or decision to submit a report.

Data Sharing Statement

Due to ethical concerns regarding the sensitive nature of the research, transcripts of qualitative interviews are not available for data sharing. All other data supporting this study are provided as supplementary information accompanying this paper.

REFERENCES

1 Table 4: All STI diagnoses & services by gender & sexual risk, 2010 - 2014. Public Health England. https://www.gov.uk/government/statistics/sexually-transmitted-infections-stis-annual-data-tables (accessed 29 Mar2016).

2 Horner P, Blee K, O'Mahony C, *et al.* 2015 UK National Guideline on the management of non-gonococcal urethritis. *Int J STD AIDS* 2016;**27**:85–96. doi:10.1177/0956462415586675

3 Pond MJ, Nori A V, Patel S, *et al.* Performance evaluation of automated urine microscopy as a rapid, non-invasive approach for the diagnosis of non-gonococcal urethritis. *Sex Transm Infect* 2015;**91**:165–70. doi:10.1136/sextrans-2014-051761

4 Smith R, Copas AJ, Prince M, *et al.* Poor sensitivity and consistency of microscopy in the diagnosis of low grade non-gonococcal urethritis. *Sex Transm Infect* 2003;**79**:487–90. doi:10.1136/sti.79.6.487

5 Menon-Johansson AS, McNaught F, Mandalia S, *et al.* Texting decreases the time to treatment for genital Chlamydia trachomatis infection. *Sex Transm Infect* 2006;**82**:49–51. doi:10.1136/sti.2004.014381

6 Bowie WR, Alexander ER, Stimson JB, *et al.* Therapy for nongonococcal urethritis: double-blind randomized comparison of two doses and two durations of minocycline. *Ann Intern Med* 1981;**95**:306–11.http://www.ncbi.nlm.nih.gov/pubmed/7271091 (accessed 24 Mar2016).

7 Horner P, Thomas B, Gilroy CB, *et al.* Role of Mycoplasma genitalium and Ureaplasma urealyticum in acute and chronic nongonococcal urethritis. *Clin Infect Dis* 2001;**32**:995–1003. doi:10.1086/319594

8 Darroch J, Myers L, Cassell J. Sex differences in the experience of testing positive for genital chlamydia infection: a qualitative study with implications for public health and for a national screening programme. *Sex Transm Infect* 2003;**79**:372–3.http://www.ncbi.nlm.nih.gov/pubmed/14573831 (accessed 10 Mar2016).

9 Kangas I, Andersen B, Olesen F, *et al.* Psychosocial impact of Chlamydia trachomatis testing in general practice. *Br J Gen Pract* 2006;**56**:587–93.http://www.ncbi.nlm.nih.gov/pubmed/16882376 (accessed 10 Mar2016).

10 Holgate HS, Longman C. Some peoples' psychological experiences of attending a sexual health clinic and having a sexually transmitted infection. *J R Soc Health* 1998;**118**:94–6.http://www.ncbi.nlm.nih.gov/pubmed/10076643 (accessed 10 Mar2016).

11 Morris JL, Lippman SA, Philip S, *et al.* Sexually transmitted infection related stigma and shame among African American male youth: implications for testing practices, partner notification, and treatment. *AIDS Patient Care STDS* 2014;**28**:499–506. doi:10.1089/apc.2013.0316

12 Mills N, Daker-White G, Graham A, *et al.* Population screening for Chlamydia trachomatis infection in the UK: a qualitative study of the experiences of those screened. *Fam Pract* 2006;**23**:550–7. doi:10.1093/fampra/cml031

13 Gottlieb SL, Stoner BP, Zaidi AA, *et al.* A prospective study of the psychosocial impact of a positive Chlamydia trachomatis laboratory test. *Sex Transm Dis* 2011;**38**:1004–11. doi:10.1097/OLQ.0b013e31822b0bed

14 Kangas I, Andersen B, Olesen F, *et al.* Psychosocial impact of Chlamydia trachomatis testing in general practice. *Br J Gen Pract* 2006;**56**:587–93.http://www.ncbi.nlm.nih.gov/pubmed/16882376 (accessed

For peer review only - http://bmjopen.bmj.com/site/about/guidelines.xhtml

27 Sep2016).

15 Robertson S, Williamson P. Men and health promotion in the UK: Ten years further on? *Health Educ J* 2005;**64**:293–301. doi:10.1177/001789690506400402

16 Cameron E, Bernardes J. Gender and disadvantage in health: men's health for a change. *Sociol Heal & Illn* 1998;**20**:673–93. doi:10.1111/1467-9566.00124

17 Connell R. *Masculinities*. University of California Press 2005. http://www.ucpress.edu/book.php?isbn=9780520246980 (accessed 28 Mar2017).

18 Beyond Men Behaving Badly: A Meta-Ethnography of Men's Perspectives on Psychological Distress and Help Seeking. doi:10.3149/jmh.1103.202

19 Collumbien M, Hawkes S. Missing men's messages: Does the reproductive health approach respond to men's sexual health needs? *Cult Health Sex* 2000;**2**:135–50. doi:10.1080/136910500300769

20 Banks I. No man's land: men, illness, and the NHS. *BMJ* 2001;**323**.http://www.bmj.com/content/323/7320/1058.long (accessed 31 Mar2017).

21 Rosaleen O, Hunt K, Hart G, *et al. Social science & medicine.* Elsevier Science 2005. http://econpapers.repec.org/article/eeesocmed/v_3a61_3ay_3a2005_3ai_3a3_3ap_3a503-516.htm (accessed 28 Mar2017).

22 Sonnenberg P, Clifton S, Beddows S, *et al.* Prevalence, risk factors, and uptake of interventions for sexually transmitted infections in Britain: findings from the National Surveys of Sexual Attitudes and Lifestyles (Natsal). *Lancet (London, England)* 2013;**382**:1795–806. doi:10.1016/S0140-6736(13)61947-9

23 Herdman M, Gudex C, Lloyd A, *et al.* Development and preliminary testing of the new five-level version of EQ-5D (EQ-5D-5L). *Qual Life Res* 2011;**20**:1727–36. doi:10.1007/s11136-011-9903-x

24 Petersen W. Society and the Adolescent Self-Image. Morris Rosenberg. Princeton University Press, Princeton, N.J., 1965. xii + 326 pp. $6.50. *Science (80-)* 1965;**148**:804–804. doi:10.1126/science.148.3671.804

25 Zimet GD, Dahlem NW, Zimet SG, *et al.* The Multidimensional Scale of Perceived Social Support. *J Pers Assess* 1988;**52**:30–41. doi:10.1207/s15327752jpa5201_2

26 Jones SG, Pat Patsdaughter CA, Martinez Cardenas VM. Lessons from the viagra study: methodological challenges in recruitment of older and minority heterosexual men for research on sexual practices and risk behaviors. *J Assoc Nurses AIDS Care* 2011;**22**:320–9. doi:10.1016/j.jana.2010.10.009

27 Mulholland E, Van Wersch A. Stigma, sexually transmitted infections and attendance at the GUM Clinic: an exploratory study with implications for the theory of planned behaviour. *J Health Psychol* 2007;**12**:17–31. doi:10.1177/1359105306069098

28 Lorimer K, Reid ME, Hart GJ. "It has to speak to people's everyday life..." qualitative study of men and women's willingness to participate in a non-medical approach to Chlamydia trachomatis screening. *Sex Transm Infect* 2009;**85**:201–5. doi:10.1136/sti.2008.031138

29 Burkill S, Copas A, Couper MP, *et al.* Using the Web to Collect Data on Sensitive Behaviours: A Study Looking at Mode Effects on the British National Survey of Sexual Attitudes and Lifestyles. *PLoS One* 2016;**11**:e0147983. doi:10.1371/journal.pone.0147983

30 Council NR. *Nonresponse in Social Science Surveys*. Washington, D.C.: : National Academies Press 2013. doi:10.17226/18293

31 Marsden P V., Wright JD. *Handbook of survey research*. Emerald 2010. https://books.google.co.uk/books/about/Handbook_of_Survey_Research.html?id=mMPDPXpTP-0C (accessed 28 Mar2017).

32 Low N, Sterne JA, Barlow D. Inequalities in rates of gonorrhoea and chlamydia between black ethnic groups in south east London: cross sectional study. *Sex Transm Infect* 2001;**77**:15–20.http://www.ncbi.nlm.nih.gov/pubmed/11158686 (accessed 28 Mar2017).

33 Le Polain De Waroux O, Harris RJ, Hughes G, *et al.* The epidemiology of gonorrhoea in London: a Bayesian spatial modelling approach. *Epidemiol Infect* 2013;**142**:1–10. doi:10.1017/S0950268813000745

34 Murray BL. Qualitative research interviews: therapeutic benefits for the participants. *J Psychiatr Ment Health Nurs* 2003;**10**:233–6.http://www.ncbi.nlm.nih.gov/pubmed/12662341 (accessed 28 Mar2017).

35 Hutchinson SA, Wilson ME, Wilson HS. Benefits of Participating in Research Interviews. *Image J Nurs Scholarsh* 1994;**26**:161–6. doi:10.1111/j.1547-5069.1994.tb00937.x

36 Lindberg C, Lewis-Spruill C, Crownover R. Barriers to sexual and reproductive health care: urban male adolescents speak out. *Issues Compr Pediatr Nurs* 2006;**29**:73–88. doi:10.1080/01460860600677577

37 Shoveller JA, Knight R, Johnson J, *et al.* 'Not the swab!' Young men's experiences with STI testing. *Sociol Health Illn* 2010;**32**:57–73. doi:10.1111/j.1467-9566.2009.01222.x

For peer review only - http://bmjopen.bmj.com/site/about/guidelines.xhtml

38 Fenton KA, Johnson AM, McManus S, *et al.* Measuring sexual behaviour: methodological challenges in survey research. *Sex Transm Infect* 2001;**77**:84–92.http://www.ncbi.nlm.nih.gov/pubmed/11287683 (accessed 24 Mar2016).

39 Miyai T, Turner KR, Kent CK, *et al.* The psychosocial impact of testing individuals with no history of genital herpes for herpes simplex virus type 2. *Sex Transm Dis* 2004;**31**:517–21.http://www.ncbi.nlm.nih.gov/pubmed/15480111 (accessed 6 Dec2016).

For peer review only

For peer review only - http://bmjopen.bmj.com/site/about/guidelines.xhtml

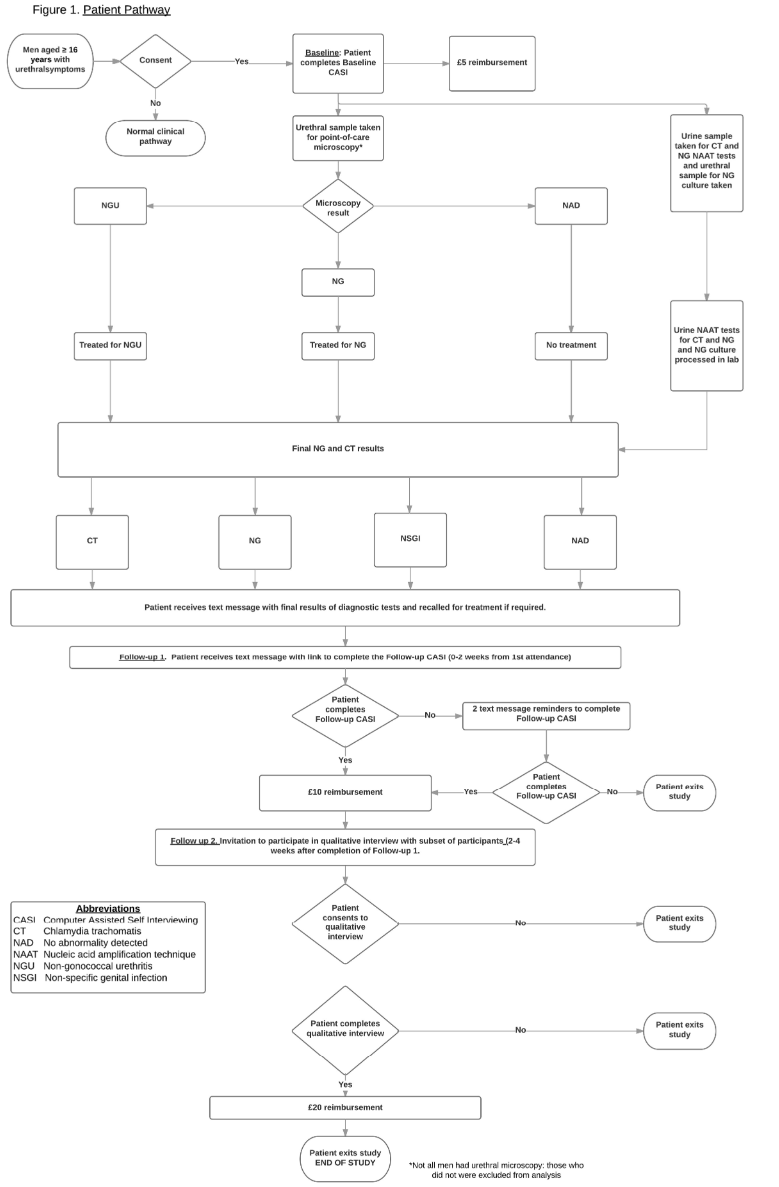

Figure 1. Patient Pathway

279x421mm (300 x 300 DPI)

Figure 2. Enrolment and Follow-up Chart

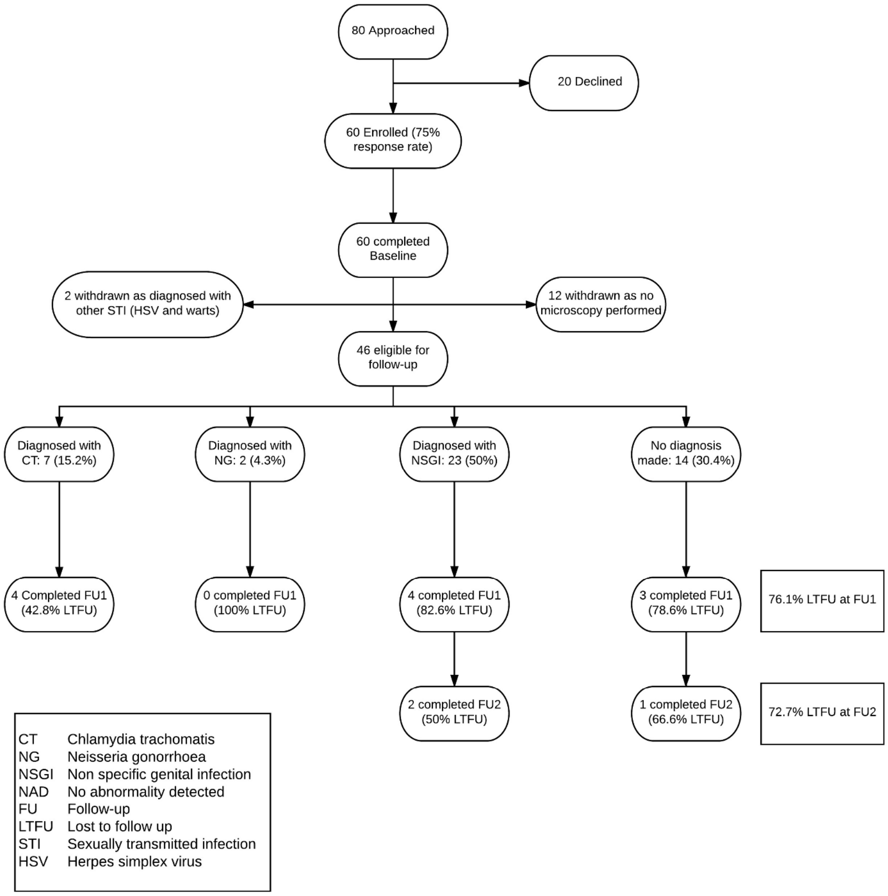

Figure 2. Enrolment and Follow-up Flow-chart

215x279mm (300 x 300 DPI)

Supplementary Appendix 1.

Baseline CASI Questionnaire

The following questions are about your background

1. Are you in full time employment? Yes/No/ Prefer not to answer

2. How old were you when you left full-time education?
    - ☐ 16 years or less
    - ☐ 17 or 18 years
    - ☐ 19 years or over
    - ☐ Still in full-time education

3. What is your highest educational achievement?
    - ☐ No qualifications / No formal qualifications
    - ☐ 1-4 GCSEs or equivalent qualifications
    - ☐ 5 GCSEs or equivalent qualifications.
    - ☐ Apprenticeships
    - ☐ 2 or more A-levels or equivalent qualifications.
    - ☐ Bachelors degree or equivalent, and higher qualifications.
    - ☐ Other qualifications including foreign qualifications.

5. Do you have any of the following long-standing conditions? (Tick ALL that apply)
    - ☐ Deafness or severe hearing impairment
    - ☐ Blindness or partially sighted
    - ☐ A long-standing physical condition
    - ☐ A learning disability
    - ☐ A mental health condition
    - ☐ A long-standing illness, such as cancer, HIV, diabetes, chronic heart disease, or epilepsy

6. Does this condition(s) cause you difficulty with any of the following? (Tick ALL that apply)
    - ☐ Everyday activities that people your age can usually do
    - ☐ At work, in education, or training
    - ☐ Access to buildings, streets or vehicles
    - ☐ Reading or writing
    - ☐ People's attitudes to you because of your condition
    - ☐ Communicating, mixing with others, or socialising
    - ☐ Any other activity
    - ☐ No difficulty with any of these

7. To which of these ethnic groups would you say you belong? (Tick ONE only)

a. WHITE
- ☐ English/Welsh/Scottish/Northern Irish/British
- ☐ Irish
- ☐ Gypsy or Irish Traveller

For peer review only

- ☐ Any other White background, write in...

b. MIXED / MULTIPLE ETHNIC GROUPS

- ☐ White and Black Caribbean
- ☐ White and Black African
- ☐ White and Asian
- ☐ Any other Mixed/multiple ethnic background, write in...

c. ASIAN / ASIAN BRITISH

- ☐ Indian
- ☐ Pakistani
- ☐ Bangladeshi
- ☐ Chinese
- ☐ Any other Asian background, write in...

d. BLACK / AFRICAN / CARIBBEAN / BLACK BRITISH

- ☐ African
- ☐ Caribbean
- ☐ Any other Black / African / Caribbean background, write in...

e. OTHER ETHNIC GROUP

- ☐ Arab
- ☐ Any other ethnic group, write in...

The following are questions about recent infections

8. Have you ever been diagnosed with a sexually transmitted infection? If so which?
TICK ALL THAT APPLY

- ☐ Chlamydia
- ☐ Gonorrhoea
- ☐ Genital Herpes
- ☐ Non Specific Genital Infection (NSGI)
- ☐ Genital Warts
- ☐ Syphilis
- ☐ HIV
- ☐ Other (write in)
- ☐ Prefer not to answer

The following questions are about relationships and break-ups

9. At present are you...

- ☐ Married and living with wife
- ☐ Cohabiting but not married
- ☐ Separated, divorced, or, widowed
- ☐ Single (that is never married or never registered in a same-sex civil partnership)
- ☐ Prefer not to answer

9a. How long have you been in this relationship for?

- ☐ 0 – 6 months

For peer review only

- ☐ More than 6 months but less than a year
- ☐ 1 – 2 years
- ☐ 2 or more years
- ☐ Prefer not to answer

10. In the last 30 days, have you broken up with someone you considered yourself to be in a relationship with (for example, your girlfriend or wife)?
    - ☐ Yes
    - ☐ No
    - ☐ Prefer not to answer

On the following screens please tap the statement which best describes your own health state today.

11. MOBILITY
    - ☐ I have no problems in walking about
    - ☐ I have slight problems in walking about
    - ☐ I have moderate problems in walking about
    - ☐ I have severe problems in walking about
    - ☐ I am unable to walk about

12. SELF-CARE
    - ☐ I have no problems washing or dressing myself
    - ☐ I have slight problems washing or dressing myself
    - ☐ I have moderate problems washing or dressing myself
    - ☐ I have severe problems washing or dressing myself
    - ☐ I am unable to wash or dress myself

13. USUAL ACTIVITIES (e.g. work, Study, housework, family or leisure activities)
    - ☐ I have no problems doing my usual activities
    - ☐ I have slight problems doing my usual activities
    - ☐ I have moderate problems doing my usual activities
    - ☐ I have severe problems doing my usual activities
    - ☐ I am unable to do my usual activities

14. PAIN / DISCOMFORT
    - ☐ I have no pain or discomfort
    - ☐ I have slight pain or discomfort
    - ☐ I have moderate pain or discomfort
    - ☐ I have severe pain or discomfort
    - ☐ I have extreme pain or discomfort

15. ANXIETY / DEPRESSION
    - ☐ I am not anxious or depressed
    - ☐ I am slightly anxious or depressed

For peer review only

- ☐ I am moderately anxious or depressed
- ☐ I am severely anxious or depressed
- ☐ I am extremely anxious or depressed

We would like to know how good or bad your health is TODAY.

This scale is numbered from 0 to 100.
100 means the best health you can imagine. 0 means the worst health you can imagine.

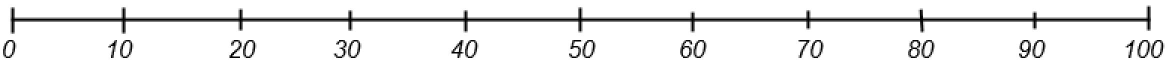

16. Please write the number you think best describes how you feel today. ______________

Below is a list of statements dealing with your general feelings about yourself. Please indicate how strongly you agree or disagree with each statement.

Strongly Agree Agree Disagree Strongly Disagree

17. On the whole, I am satisfied with myself.
18. At times I think I am no good at all.
19. I feel that I have a number of good qualities.
20. I am able to do things as well as most other people.
21. I feel I do not have much to be proud of.
22. I certainly feel useless at times.
23. I feel that I'm a person of worth, at least on an equal plane with others.
24. I wish I could have more respect for myself.
25. All in all, I am inclined to feel that I am a failure.
26. I take a positive attitude toward myself.

Below is a list of statements dealing with your general feelings about your support from people around you. Please indicate how strongly you agree or disagree with each statement.
1 Very Strongly Disagree, 2 Strongly Disagree, 3 Mildly Disagree, 4 Neutral, 5 Mildly Agree, 6 Strongly Agree, 7 Very strongly agree

27. There is a special person who is around when I am in need
28. There is a special person with whom I can share my joys and sorrows
29. My family really tries to help me
30. I get all the emotional help and support I need from my family
31. I have a special person who is a real source of comfort to me
32. My friends really try to help me

For peer review only

33. I can count on my friends when things go wrong
34. I can talk about my problems with my family
35. I have friends with whom I can share my joys and sorrows
36. There is a special person in my life who cares about my feelings
37. My family is willing to help me make decisions.
38. I can talk about my problems with my friends.

We would now like to ask you if you have any feedback for us on how you found completing this questionnaire

39. How did you feel about completing this series of questions? Please write any comments in the space below.

____

____

____

Thank-you for completing this questionnaire
If you would like to speak to someone about the issues raised in this questionnaire please contact us.

For peer review only

Supplementary Appendix 2.

Follow-up 1 CASI Questionnaire

On the following screens please tap the statement which best describes your own health state today.

1. MOBILITY
   - ☐ I have no problems in walking about
   - ☐ I have slight problems in walking about
   - ☐ I have moderate problems in walking about
   - ☐ I have severe problems in walking about
   - ☐ I am unable to walk about

2. SELF-CARE
   - ☐ I have no problems washing or dressing myself
   - ☐ I have slight problems washing or dressing myself
   - ☐ I have moderate problems washing or dressing myself
   - ☐ I have severe problems washing or dressing myself
   - ☐ I am unable to wash or dress myself

3. USUAL ACTIVITIES (e.g. work, Study, housework, family or leisure activities)
   - ☐ I have no problems doing my usual activities
   - ☐ I have slight problems doing my usual activities
   - ☐ I have moderate problems doing my usual activities
   - ☐ I have severe problems doing my usual activities
   - ☐ I am unable to do my usual activities

4. PAIN / DISCOMFORT
   - ☐ I have no pain or discomfort
   - ☐ I have slight pain or discomfort
   - ☐ I have moderate pain or discomfort
   - ☐ I have severe pain or discomfort
   - ☐ I have extreme pain or discomfort

5. ANXIETY / DEPRESSION
   - ☐ I am not anxious or depressed
   - ☐ I am slightly anxious or depressed
   - ☐ I am moderately anxious or depressed
   - ☐ I am severely anxious or depressed
   - ☐ I am extremely anxious or depressed

We would like to know how good or bad your health is TODAY.

This scale is numbered from 0 to 100.

For peer review only

100 means the best health you can imagine. 0 means the worst health you can imagine.

0 10 20 30 40 50 60 70 80 90 100

6. Please write the number you think best describes how you feel today. ______________

7. After you had received all your test results from your most recent visit to the GUM clinic were you diagnosed with any of the following infections: Chlamydia, Gonorrhoea or Non-specific urethritis (NSU)? ?
   - ☐ Yes
   - ☐ No
   - ☐ Not sure

The following questions are about your reaction after you were told your test was positive

1= Strongly Disagree 2 = Disagree 3 = Neutral 4 = Agree 5 = Strongly Agree

<u>After I was told that my test was positive:</u>

8. I talked to my friend(s) about it.	1	2	3	4	5
9. I do not think it was a big deal.	1	2	3	4	5
10. I was/am scared to tell my partner(s).	1	2	3	4	5
11. It does not change the way I feel about myself.	1	2	3	4	5
12. My partner thinks that I cheated on him or her.	1	2	3	4	5
13. I am not worried since it is curable.	1	2	3	4	5
14. I feel like I did not take care of myself.	1	2	3	4	5
15. I feel betrayed by my partner.	1	2	3	4	5
16. I worry that it will not go away.	1	2	3	4	5
17. I feel guilty that I could have given someone my infection	1	2	3	4	5
18. It has not changed my relationship with my partner.	1	2	3	4	5
19. I am not very proud of my actions.	1	2	3	4	5
20. I do not blame myself.	1	2	3	4	5
21. I think that my partner cheated on me.	1	2	3	4	5
22. I worry that I might not be able to have children in the future.	1	2	3	4	5
23. I do not really care that much.	1	2	3	4	5
24. I am angry.	1	2	3	4	5
25. I feel really bad about myself.	1	2	3	4	5
26. It does not change how my friends feel about me.	1	2	3	4	5
27. It will be hard for me to trust future partners.	1	2	3	4	5
28. I am not worried about getting it again.	1	2	3	4	5
29. I feel dirty.	1	2	3	4	5
30. I broke up with my partner.	1	2	3	4	5

31. I worry about what other sexually transmitted infections I could have been exposed to.	1	2	3	4	5
32. I feel really embarrassed.	1	2	3	4	5

The next questions are about how you feel generally about the infection you were diagnosed with

33. I think about the infection I was diagnosed with a lot.
1= Strongly Disagree 2 = Disagree 3 = Neutral 4 = Agree 5 = Strongly Agree

34. I am concerned about the infection I was diagnosed with.
1= Strongly Disagree 2 = Disagree 3 = Neutral 4 = Agree 5 = Strongly Agree

Below is a list of statements dealing with your general feelings about yourself. Please indicate how strongly you agree or disagree with each statement.

Strongly Agree Agree Disagree Strongly Disagree

35. On the whole, I am satisfied with myself.
36. At times I think I am no good at all.
37. I feel that I have a number of good qualities.
38. I am able to do things as well as most other people.
39. I feel I do not have much to be proud of.
40. I certainly feel useless at times.
41. I feel that I'm a person of worth, at least on an equal plane with others.
42. I wish I could have more respect for myself.
43. All in all, I am inclined to feel that I am a failure.
44. I take a positive attitude toward myself.

The following questions are about relationships and break-ups

45. At present are you...
- ☐ married and living with wife
- ☐ cohabiting but not married
- ☐ separated, divorced, or, widowed
- ☐ single (that is never married)
- ☐ prefer not to answer

45a. How long have you been in this relationship for?
- ☐ 0 – 6 months
- ☐ More than 6 months but less than a year
- ☐ 1 – 2 years
- ☐ 2 or more years
- ☐ Prefer not to answer

For peer review only

46. In the last 30 days, have you broken up with someone you considered yourself to be in a relationship with (for example, your girlfriend or wife)?

- ☐ Yes
- ☐ No
- ☐ Prefer not to answer

The next questions are about your most recent visit to the GUM Clinic

47. Have you ever visited this GUM Clinic before for the same condition?

- ☐ Yes
- ☐ No
- ☐ Prefer not to answer

48. Did a member of staff explain the results of the tests in a way you could understand?

- ☐ Yes definitely
- ☐ Yes, to some extent
- ☐ No
- ☐ Not sure, can't remember
- ☐ I was told I would get the results at a later date
- ☐ I was never told the results of the tests

49. While you were in the GUM Clinic how much information about your condition or treatment was given to you?

- ☐ Not enough
- ☐ Right amount
- ☐ Too much
- ☐ I was not given any information about my treatment or condition

50. Was the main reason you went to the GUM Clinic dealt with to your satisfaction?

- ☐ Yes, completely
- ☐ Yes, to some extent
- ☐ No

The next questions are about the symptoms you had when attending the clinic (the time you were first asked to join this study)

51. Please choose the option that best fits how you feel

- ☐ My symptoms have completely gone away
- ☐ My symptoms are better but not completely
- ☐ My symptoms are the same
- ☐ My symptoms are worse than they were before
- ☐ Don’t know/not sure
- ☐ Prefer not to answer

For peer review only

We would now like to ask you if you have any feedback for us on how you found completing this questionnaire

52. How did you feel about completing this series of questions? Please write any comments in the space below.

____

____

____

Thank-you for completing this questionnaire
If you would like to speak to someone about the issues raised in this questionnaire please contact us.

For peer review only

Supplementary Appendix 3.

Individual in-depth interview Topic Guide

Topics

Life history / framing (~5 minutes)

Please tell me a bit about yourself.

What is the most important thing you can tell me about yourself?

Probes:

Hobbies/interests

Goals

Tell me about your family.

Who do you include in your family? Partner/spouse? Father/mother? Grandparents? Children?

What sorts of things do you do with your day? / Describe a typical day in your life.

Probes:

Are you in school/university?

What do you do for money?

Clinical experience (~25 minutes)

Please describe how you were feeling while you were at the clinic on the day you were asked to take part in this study.

Probes:

Describe the reasons why you came to clinic

(If symptoms) What affect (if any) did your symptoms have on your day-to-day life?

How did it go/ how did you feel about the visit?

How long were you waiting to be seen? Was this expected /unexpected?

Which infections were you tested for?

What did the doctor tell you they thought you had? (i.e. diagnosis, if any)

Now let's discuss your diagnosis.

What thoughts were going through your mind when you were told you had NSU/Ng/possibly an STI (use patients' own description/language for their diagnosis)?

Probes:

How did you feel about your diagnosis?

For peer review only

Have you been diagnosed with NSU/Ng before? Any other infections? If yes, please describe any differences in your feelings from the first time you were diagnosed compared with this recent diagnosis.

Did you tell anyone (partner/family members/friends) your diagnosis? If yes, who and why? What was their reaction?

Research experience (~15 minutes)

We want to make sure that the research we conduct is done in the best way possible for patients. This next section of our interview is based on your experience with this research study so far. Please remember that your answers to these questions will not affect your clinical care, your legal rights, or future invitations to participate in research. It is important that you speak freely so that we can improve future research in this area.

How did you feel about your participation in the research?

Probes:

Would you consider involving yourself in this kind of research in the future? Why/why not?

Were you able to complete the surveys?

Did you have any problem accessing the CASI? (Logistical/technical problems) If so, what were the problems you faced?

Did you feel comfortable accessing the CASI (inside/outside of clinic)? Why/why not?

How did you feel about the questionnaires?

Discuss each questionnaire individually in turn: demographic and clinic experience questions; EQ5D-5L; social support scale; self-esteem scale:

Did you feel these questions were relevant?

Did you feel comfortable answering these questions? Why/why not?

Discuss any suggestions you may have for improvement of these questionnaires

Anything else?

Thanks

Reimbursement

For peer review only

Supplementary Appendix 4.

Full list of themes for Qualitative Interview (Follow-up 2)

List of nodes
antibiotics
attitudes to safe sex and sexual health
consultation
different SHCs
familiarity with SHC
feelings about attending SHC
feelings about being interviewed
feelings about participation in the research
feelings about positive diagnosis
future research
HIV vs. other STIs
interview
invasive tests
NSGI vs. CT
obstacles to participation in research
perception of CT
reaction to NSGI
reasons for participating in research
relationship
reasons for attending SHC
results
risk perception
social support
STI prevention
suggestions for research
survey - was it easy
survey - was it engaging
survey - was it relevant
survey comfort
treatment
waiting for results
waiting time in SHC

For peer review only

STROBE Statement—checklist of items that should be included in reports of observational studies

	Item No.	Recommendation	Page No.	Relevant text from manuscript
**Title and abstract**	1	(*a*) Indicate the study's design with a commonly used term in the title or the abstract	1-2	We conducted an observational study among symptomatic heterosexual men attending SHCs to evaluate the psychosocial impact of an NSGI diagnosis compared to a diagnosis of CT, NG or no abnormalities detected (NAD) focussing on the feasibility of our study methodology.
		(*b*) Provide in the abstract an informative and balanced summary of what was done and what was found	1-2	**ABSTRACT**<br>**Introduction:**<br>Non-specific genital infection (NSGI; non-*Chlamydia trachomatis* (CT), non-*Neisseria gonorrhoeae* (NG) associated urethritis) is a common diagnosis in symptomatic heterosexual men attending UK Sexual Health Clinics (SHCs) but little is known about the psychosocial impact of this diagnosis.<br>**Methods:**

For peer review only - http://bmjopen.bmj.com/site/about/guidelines.xhtml

We conducted an observational study among symptomatic heterosexual men attending SHCs to evaluate the psychosocial impact of an NSGI diagnosis compared to a diagnosis of CT, NG or no abnormalities detected (NAD) focussing on the feasibility of our study methodology. Participants completed a Computer Assisted Self Interview (CASI) including validated health-related quality-of-life (HRQoL) and self-esteem measures before diagnostic testing and two-weeks after receiving test results, and a qualitative interview. We compared scores between diagnostic groups using paired t-tests, qualitative data were analysed thematically, and feasibility was assessed by process analysis.

Results:

For peer review only - http://bmjopen.bmj.com/site/about/guidelines.xhtml

60 men completed the baseline CASI (75% response rate). 46 (76.6%) were eligible for follow-up; of these, 11 (23.9%) completed the follow-up CASI, and 3 (27.3%) completed the qualitative interview. 81.7% of participants left CASI feedback at baseline: 73.5% reported the questionnaire as ‘fine’ or ‘very good’. Qualitative interview participants reported the study was acceptable. All patients completing FU-1 showed measurable decreases from baseline to FU-1 in self-esteem (CT p=0.009; NSGI p=0.008; NAD p=0.057). Measurable decreases in HRQoL from baseline to FU-1 were found among participants with CT (p=0.002) or NSGI (p=0.001). We did not find differences in baseline sociodemographic factors between participants who completed FU-1 and those that did not.

For peer review only

				**Conclusions:** Although most participants indicated study acceptability at baseline, and we employed measures to increase retention (CASI questionnaires, reminder messages, a focus on men's health), attrition was high. We found that heterosexual men attending SHCs with symptoms of urethritis may experience negative psychosocial impacts regardless of their diagnosis, which warrants further investigation. We recommend exploring novel strategies to decrease attrition in future studies.
**Introduction**				
Background/rationale	2	Explain the scientific background and rationale for the investigation being reported	2-4	**INTRODUCTION** Non-specific Genital Infection (NSGI) or, non-*Chlamydia trachomatis* (CT), non-*Neisseria gonorrhoeae* (NG) associated urethritis (NGU), is the third

For peer review only - http://bmjopen.bmj.com/site/about/guidelines.xhtml

For peer review only

most frequently diagnosed condition in heterosexual men attending sexual health clinics (SHCs) in the United Kingdom (UK), with 30,381 cases/year compared to CT (42,483/year) and first presentation of genital warts (32,656/year) [1]. NSGI is a diagnosis of exclusion; men with urethral symptoms are screened with urethral microscopy in SHCs, and if this demonstrates urethritis with no gonococci, a provisional diagnosis of NGU is made [2]. If urinary Nucleic Acid Amplification Tests (NAATs) sent for NG and CT are both negative, the diagnosis is recorded as NSGI.

Although a common condition, NSGI remains poorly understood. NSGI can be caused by a variety of sexually transmitted infections (STIs), including *Mycoplasma*

For peer review only - http://bmjopen.bmj.com/site/about/guidelines.xhtml

genitalium (MG) and *Trichomonas vaginalis* (TV), which are not routinely tested for in SHCs, and many cases have an uncertain aetiology [2]. Microscopy, on which the diagnosis rests, has poor specificity for known causes of urethritis [3] and is subject to considerable observer variation [4].

National guidelines recommend that men diagnosed with NGU are informed they may have an STI, given same day empirical antibiotics, advised their partners should attend for STI testing and empirical treatment, and to abstain from sex until treatment is completed [2]. Men receive CT/NG NAAT results usually within 8 days [5]. Following first-line antibiotics for NSGI, 10%–20% of men return because of persistent symptoms [6,7].

For peer review only - http://bmjopen.bmj.com/site/about/guidelines.xhtml

Men who undergo STI testing, and are diagnosed with an STI report negative impacts on their psychosocial wellbeing, including feelings of stigma [8–10], however, to our knowledge, there are no published data on the psychosocial impact of NSGI diagnosis among men. Stigma has been shown in men to be associated with a delay in testing for STIs, and decreased willingness to notify their casual partners of their STI diagnosis [11]. Studies on psychosocial impact, partner notification and quality of life impact have predominantly been carried out in women [9,12–14], with few studies recruiting men. A greater understanding of the degree and nature of psychosocial impacts of a diagnosis of an STI, or a 'presumed STI' such as NSGI, in men, is important to optimise

For peer review only - http://bmjopen.bmj.com/site/about/guidelines.xhtml

interventions, including partner notification and risk-behaviour modification, which aim to reduce the negative impact of STIs, such as complications of infection.

Men are reportedly poor users of health services, largely due to culturally dominant ideals of masculinity that equate illness with weakness, which may impede care-seeking [15–18]. In sexual healthcare, there has traditionally been a focus on female reproductive health [19], and this may further discourage male attendance [19,20]. However, men are willing to respond openly to questions about their sexual health, particularly when enquiries are focused specifically on men's needs [19,21].

We decided to focus on heterosexual men in this study

For peer review only - http://bmjopen.bmj.com/site/about/guidelines.xhtml

as there are important differences between men who have sex with men (MSM) and men who have sex exclusively with women (MSEW) in their access of SHC services, reported sexual health risks, their own perception of risk of STIs, and sexual health and well being outcomes, suggesting that their experience of diagnosis of a genital infection may also be different. The Natsal-3 study reported that in comparison with MSEW, MSM are more likely to report higher numbers of partners, higher rates of condomless sex, to perceive themselves at higher rates of STIs, a greater likelihood to attend SHCs, and higher rates of depression, substance use and non-volitional sex [22]. Due to these important differences between MSM and MSEW, we decided that psychosocial impacts of an STI diagnosis in

For peer review only - http://bmjopen.bmj.com/site/about/guidelines.xhtml

				MSM merited studying separately.<br><br>Because of a lack of models to measure the psychosocial impact of an NSGI diagnosis among men, and the potential difficulty in recruiting and retaining this group in sexual health studies, we designed a small-scale longitudinal mixed-method feasibility study to inform on our research methods and better understand the psychosocial and health related quality of life (HRQoL) status of symptomatic heterosexual men before and after receiving a diagnosis of NSGI, CT, NG or nothing abnormal detected (NAD).
Objectives	3	State specific objectives, including any prespecified hypotheses	4	Because of a lack of models to measure the psychosocial impact of an NSGI diagnosis among men, and the potential difficulty in recruiting and retaining this group in sexual

For peer review only - http://bmjopen.bmj.com/site/about/guidelines.xhtml

For peer review only

				health studies, we designed a small-scale longitudinal mixed-method feasibility study to inform on our research methods and better understand the psychosocial and health related quality of life (HRQoL) status of symptomatic heterosexual men before and after receiving a diagnosis of NSGI, CT, NG or nothing abnormal detected (NAD).
**Methods**				
Study design	4	Present key elements of study design early in the paper	4	We conducted a longitudinal study in symptomatic heterosexual men using patient completed questionnaires assessing HRQoL and psychosocial status before and after receiving STI test results, and individual qualitative interviews to evaluate experiences at the SHC and acceptability of study methods.
Setting	5	Describe the setting, locations, and relevant dates, including periods of recruitment, exposure, follow-up, and data collection	4-5	Eligibility criteria were: heterosexual men presenting with

For peer review only - http://bmjopen.bmj.com/site/about/guidelines.xhtml

urethral symptoms (dysuria, discharge or urethral discomfort), aged ≥16 years at either of two participating London SHCs between September - October 2016. We excluded men who were asymptomatic, not available for follow-up, and who were unable to understand English.

Following informed consent, participants were invited to complete a baseline computer assisted self-interview (CASI) assessing psychosocial and HRQoL status in clinic prior to consultation, a follow-up CASI after receipt of all test results 1-2 weeks after baseline (FU-1) and a qualitative interview 2-4 weeks after baseline (FU-2).

CASIs were optimised for web-enabled devices including smartphones. We offered participants the choice to complete CASIs using their own

For peer review only - http://bmjopen.bmj.com/site/about/guidelines.xhtml

				device or a study tablet-device and use of a private room in clinic. Study staff sent ≤2 text message reminders for each follow-up. Participants who did not complete FU-1 were considered ineligible for FU-2. Participants received gift voucher reimbursements: £5 (baseline), £10 (FU-1), £20 (FU-2).
Participants	6	(*a*) *Cohort study*—Give the eligibility criteria, and the sources and methods of selection of participants. Describe methods of follow-up<br>*Case-control study*—Give the eligibility criteria, and the sources and methods of case ascertainment and control selection. Give the rationale for the choice of cases and controls<br>*Cross-sectional study*—Give the eligibility criteria, and the sources and methods of selection of participants	4-5	Eligibility criteria were: heterosexual men presenting with urethral symptoms (dysuria, discharge or urethral discomfort), aged ≥16 years at either of two participating London SHCs between September - October 2016. We excluded men who were asymptomatic, not available for follow-up, and who were unable to understand English. Following informed consent, participants were invited to complete a baseline computer assisted self-interview (CASI)

For peer review only - http://bmjopen.bmj.com/site/about/guidelines.xhtml

				assessing psychosocial and HRQoL status in clinic prior to consultation, a follow-up CASI after receipt of all test results 1-2 weeks after baseline (FU-1) and a qualitative interview 2-4 weeks after baseline (FU-2).
		(*b*) *Cohort study*—For matched studies, give matching criteria and number of exposed and unexposed<br>*Case-control study*—For matched studies, give matching criteria and the number of controls per case		
Variables	7	Clearly define all outcomes, exposures, predictors, potential confounders, and effect modifiers. Give diagnostic criteria, if applicable	5	**Diagnostic Criteria**<br>We categorised participants into four diagnostic groups based on microscopy and NAAT results: NSGI: NGU diagnosis on urethral microscopy and CT/NG NAATs negative; NG: NG on urethral microscopy and/or positive NAAT; CT: positive NAAT; NAD: no urethritis on microscopy and CT/NG NAATs negative.<br><br>**Outcome measures**<br>*Primary outcome:* Qualitative and quantitative process data on

For peer review only - http://bmjopen.bmj.com/site/about/guidelines.xhtml

For peer review only

				recruitment and retention rates at baseline and follow-up to inform on feasibility. Evaluation of potential relevance of questions validated in other populations for men diagnosed with NSGI.<br><br>*Secondary outcomes:*<br>Percentage change from baseline to FU-1 in mean EQ-5D-5L and Rosenberg Self-Esteem Scale in men diagnosed with CT, NG, NSGI or NAD.
Data sources/ measurement	8*	For each variable of interest, give sources of data and details of methods of assessment (measurement). Describe comparability of assessment methods if there is more than one group	4-5	The participant pathway is detailed in Figure 1. After completion of the baseline CASI, any participants diagnosed with an STI other than CT, NG or NSGI, or who did not have both urethral microscopy and urinary NAATs, were withdrawn from the study. Once participants were notified of their NAAT CT/NG results by the SHC (phone call/ text message), the research team

For peer review only - http://bmjopen.bmj.com/site/about/guidelines.xhtml

texted participants an electronic link to the FU-1 CASI.

Questionnaire design

Baseline and follow-up CASIs included validated scales measuring psychosocial status and HRQoL and additional questions. The EQ-5D-5L scale [17] as a measure of HRQoL and the Rosenberg Self-Esteem scale [18] as a measure of potential stigmatising effects of screening for STIs, were used in both baseline and FU-1 CASIs.

In addition to the above, the baseline CASI included the Multidimensional Scale of Social Support [19] to assess coping resources and questions on sociodemographic and current relationship status, and the FU-1 CASI included a question on current relationship status and a panel of questions

For peer review only - http://bmjopen.bmj.com/site/about/guidelines.xhtml

				previously developed to assess impact of CT diagnosis in women, [20] which we modified for use in men by removing a pregnancy-related question. Participants were asked to indicate acceptability of the CASIs (free-text response) at baseline and FU-1. (Baseline and FU-1 questionnaires and FU-2 topic guide: Appendix 1, Supplementary Information.)
Bias	9	Describe any efforts to address potential sources of bias	5-6	**Data analysis**<br>*Questionnaire data:*<br>Scores for psychosocial and HRQoL scales were calculated according to published methods [24][23][25]. For all eligible men, mean and 95% Confidence Intervals of baseline scores for EQ-5D-5L, Rosenberg Self-Esteem and Multidimensional Social Support Scales were compared in men diagnosed with CT, NG, NSGI and NAD, using paired t-tests. To enable

For peer review only - http://bmjopen.bmj.com/site/about/guidelines.xhtml

us to compare the EQ-5D-5L score across the different diagnoses (CT, NG, NSGI and NAD), a mean score was calculated: we calculated mean values for each of the five dimensions of the EQ-5D-5L, and a final score for the EQ-5D-5L was created using the sum of the mean value for each dimension. We calculated the mean value rather than an index score for the EQ-5D-5L to allow comparison between the outcomes of EQ-5D-5L with the Rosenberg Self-Esteem and Multidimensional Social Support Scales.

To test for non-response bias, we compared sociodemographic data of those who completed both baseline and follow-up to those who were lost to follow-up (LTFU) using Pearson's Chi^2 tests and Kruskal-Wallis tests. Paired t-tests were used to

For peer review only - http://bmjopen.bmj.com/site/about/guidelines.xhtml

assess differences in mean score for EQ-5D-5L and Rosenberg Self-Esteem Scales between baseline and follow-up in men diagnosed with CT, NG, NSGI and NAD. We used the students t-test distribution to calculate p-values. Statistical significance was set at $p<0.05$. Statistical analysis was carried out using STATA version 13.1 (StataCorp, 2015).

Qualitative data: Audio recordings were transcribed and transcripts checked for accuracy against the recording. Cleaned transcripts were then imported into NVivo (Version 10, 2012) for analysis. Two researchers assigned themes to text independently, with discrepancies resolved by consensus before final themes reported. All names of participants presented here are pseudonyms.

For peer review only - http://bmjopen.bmj.com/site/about/guidelines.xhtml

For peer review only

Study size	10	Explain how the study size was arrived at	5	As this was a feasibility study with no precedent, no formal sample size could be calculated. We planned to approach 100 patients at baseline as a convenience target, and assuming approximately 50% refusal, we expected 50-60 patients to be enrolled and estimated ~50% attrition at each follow-up.

Continued on next page

For peer review only

Quantitative variables	11	Explain how quantitative variables were handled in the analyses. If applicable, describe which groupings were chosen and why	5-6	**Data analysis**<br>*Questionnaire data:*<br>Scores for psychosocial and HRQoL scales were calculated according to published methods [24][23][25]. For all eligible men, mean and 95% Confidence Intervals of baseline scores for EQ-5D-5L, Rosenberg Self-Esteem and Multidimensional Social Support Scales were compared in men diagnosed with CT, NG, NSGI and NAD, using paired t-tests. To enable us to compare the EQ-5D-5L score across the different diagnoses (CT, NG, NSGI and NAD), a mean score was calculated: we calculated mean values for each of the five dimensions of the EQ-5D-5L, and a final score for the EQ-5D-5L was created using the sum of the mean value for each dimension. We calculated the mean value rather than an index score for the EQ-5D-5L to allow comparison between the outcomes of EQ-5D-5L with the

For peer review only - http://bmjopen.bmj.com/site/about/guidelines.xhtml

				Rosenberg Self-Esteem and Multidimensional Social Support Scales.<br><br>To test for non-response bias, we compared sociodemographic data of those who completed both baseline and follow-up to those who were lost to follow-up (LTFU) using Pearson's Chi^2 tests and Kruskal-Wallis tests. Paired t-tests were used to assess differences in mean score for EQ-5D-5L and Rosenberg Self-Esteem Scales between baseline and follow-up in men diagnosed with CT, NG, NSGI and NAD. We used the students t-test distribution to calculate p-values. Statistical significance was set at $p<0.05$. Statistical analysis was carried out using STATA version 13.1 (StataCorp, 2015).
Statistical methods	12	(*a*) Describe all statistical methods, including those used to control for confounding	5-6	**Data analysis**<br>*Questionnaire data:*<br>Scores for psychosocial and

For peer review only

For peer review only - http://bmjopen.bmj.com/site/about/guidelines.xhtml

HRQoL scales were calculated according to published methods [24][23][25]. For all eligible men, mean and 95% Confidence Intervals of baseline scores for EQ-5D-5L, Rosenberg Self-Esteem and Multidimensional Social Support Scales were compared in men diagnosed with CT, NG, NSGI and NAD, using paired t-tests. To enable us to compare the EQ-5D-5L score across the different diagnoses (CT, NG, NSGI and NAD), a mean score was calculated: we calculated mean values for each of the five dimensions of the EQ-5D-5L, and a final score for the EQ-5D-5L was created using the sum of the mean value for each dimension. We calculated the mean value rather than an index score for the EQ-5D-5L to allow comparison between the outcomes of EQ-5D-5L with the Rosenberg Self-Esteem and Multidimensional Social Support Scales.

For peer review only - http://bmjopen.bmj.com/site/about/guidelines.xhtml

			To test for non-response bias, we compared sociodemographic data of those who completed both baseline and follow-up to those who were lost to follow-up (LTFU) using Pearson's Chi^2 tests and Kruskal-Wallis tests. Paired t-tests were used to assess differences in mean score for EQ-5D-5L and Rosenberg Self-Esteem Scales between baseline and follow-up in men diagnosed with CT, NG, NSGI and NAD. We used the students t-test distribution to calculate p-values. Statistical significance was set at $p<0.05$. Statistical analysis was carried out using STATA version 13.1 (StataCorp, 2015).
	(*b*) Describe any methods used to examine subgroups and interactions		
	(*c*) Explain how missing data were addressed	6	Pearson's Chi^2 tests and Kruskal-Wallis test results for non-response bias were not statistically significant so we assumed data were missing at random.
	(*d*) *Cohort study*—If applicable, explain how loss to follow-up was addressed<br>*Case-control study*—If applicable, explain how matching of cases and controls was addressed		

For peer review only - http://bmjopen.bmj.com/site/about/guidelines.xhtml

		Cross-sectional study—If applicable, describe analytical methods taking account of sampling strategy		
		(*e*) Describe any sensitivity analyses		
**Results**				
Participants	13*	(a) Report numbers of individuals at each stage of study—eg numbers potentially eligible, examined for eligibility, confirmed eligible, included in the study, completing follow-up, and analysed	6	We approached 80 patients, of whom 60 (75%) enrolled and completed the baseline CASI. 46 (76.6%) were eligible for follow-up; of these, 11 (23.9%) completed the FU-1 CASI, and 3 of these (27.3%) completed FU-2 (Figure 2.)
		(b) Give reasons for non-participation at each stage		See Figure 2
		(c) Consider use of a flow diagram		See Figure 2
Descriptive data	14*	(a) Give characteristics of study participants (eg demographic, clinical, social) and information on exposures and potential confounders	6-7	Participants' sociodemographic factors at baseline are presented in Table 1. Most were between 25-35 years old (60.9%) and over half reported their ethnicity as non-White (52.2%). 80.4% indicated they were not in a relationship and many had previously been diagnosed with STIs (30.4% NSGI, 28.3% CT, 13.0% genital herpes or warts and 8.7% NG).
		(b) Indicate number of participants with missing data for each variable of interest		

For peer review only - http://bmjopen.bmj.com/site/about/guidelines.xhtml

		(c) *Cohort study*—Summarise follow-up time (eg, average and total amount)	
Outcome data	15*	*Cohort study*—Report numbers of outcome events or summary measures over time	
		Case-control study—Report numbers in each exposure category, or summary measures of exposure	
		Cross-sectional study—Report numbers of outcome events or summary measures	
Main results	16	(*a*) Give unadjusted estimates and, if applicable, confounder-adjusted estimates and their precision (eg, 95% confidence interval). Make clear which confounders were adjusted for and why they were included	See Table 2
		(*b*) Report category boundaries when continuous variables were categorized	
		(*c*) If relevant, consider translating estimates of relative risk into absolute risk for a meaningful time period	

Continued on next page

For peer review only

Other analyses	17	Report other analyses done—eg analyses of subgroups and interactions, and sensitivity analyses		
**Discussion**				
Key results	18	Summarise key results with reference to study objectives	8-11	In this small feasibility study we detected decreases in mean HRQoL and self-esteem scores in participants with a final diagnosis of CT or NSGI from the time of clinical presentation to approximately one week after receiving NAAT results. Although participants diagnosed with CT and NSGI showed greater negative impact in this time period, interestingly, participants who ultimately received a NAD diagnosis also experienced decreased self-esteem scores. Studies using qualitative methods and non-validated questionnaires have shown men diagnosed with CT experience negative psychosocial impacts [7, 8]. However, this is the first time that a negative impact of diagnosis of NSGI has been demonstrated to our knowledge.

For peer review only - http://bmjopen.bmj.com/site/about/guidelines.xhtml

				We experienced high LTFU that resulted in low numbers of respondents within diagnostic categories, which meant we were unable to adjust our analysis for common confounders, such as education and socioeconomic status, known to be associated with gonorrhoea [26,27] . This may be a source of bias in our results.<br>In interviews, participants with NSGI reported concerns regarding the uncertainty of the diagnosis, and fear of the impact of a possible STI diagnosis on their relationship. These findings suggest receipt of an STI or presumptive STI diagnosis in this population may have complex psychosocial impacts. These impacts may be multi-factorial and anxiety seems to be highly contextual and affected by relationship status and knowledge about STIs.
Limitations	19	Discuss limitations of the study, taking into account sources of potential bias or imprecision. Discuss both direction and magnitude of any potential bias	9-11	Despite these mechanisms, and

For peer review only - http://bmjopen.bmj.com/site/about/guidelines.xhtml

positive participant feedback on the baseline CASI and in our qualitative interviews, we experienced high LTFU. High LTFU resulted in low numbers of respondents within diagnostic categories, which meant we were unable to adjust our analysis for common confounders, such as education and socioeconomic status, known to be associated with gonorrhoea [32,33] . This may be a source of bias in our results. We did not find significant differences between groups in terms of non-response bias and other characteristics, however this is not surprising as the power to detect differences between groups is greatly affected by sample size and our study included a small number of participants. Recruiting larger numbers of participants in future studies will increase the statistical power to detect any important differences between groups.

A weakness of our study is that we

For peer review only - http://bmjopen.bmj.com/site/about/guidelines.xhtml

did not include asymptomatic men in our study, hence we cannot exclude the possibility that the negative psychosocial impacts observed with men diagnosed with CT or NSGI were not related to the confounding factors of being symptomatic or the attendance at an SHC (including undergoing genital examination, and a urethral swab). Few studies have examined the impact of an SHC clinic visit on psychosocial and/or self-esteem outcomes for men, but those that have been conducted did report negative impacts; studies among heterosexual men attending SHCs who are diagnosed with an STI reported stigma, anxiety, isolation, concerns regarding relationships, feeling 'contaminated' and fear of exposure, as well as reluctance to attend an SHC clinic for help [27,36]. A focus group of young male adolescents reported that accessing SHC services was stressful due to fear of stigma and a

For peer review only - http://bmjopen.bmj.com/site/about/guidelines.xhtml

loss of social status, shame, and embarrassment, a lack of privacy/confidentiality, and challenges in accessing and negotiating the healthcare system[36]. Shoveller et al reported that factors involved in STI testing, such as urethral swab testing, were seen by men as fearful, and as a process where men felt very vulnerable; most of these male participants reported feeling anxious waiting for potentially bad news in addition to feeling that their masculinity had been compromised through an admission of needing help [37]. Limitations of these studies are that they did not specify whether patients had symptoms, or if men had experienced (invasive or non-invasive) testing procedures, and tended to recruit patients who had already been diagnosed with an STI, which makes the impact of testing and receiving a diagnosis difficult to separate [10,27,36,37]. Men who used postal screening for

For peer review only - http://bmjopen.bmj.com/site/about/guidelines.xhtml

CT and had negative test results were not found to experience a negative impact on self-esteem or anxiety, suggesting that STI testing at home may reduce negative impacts associated with visiting an SHC, however this study did not follow-up men with positive CT diagnoses[12]. Future studies should consider including asymptomatic men alongside symptomatic men, to better evaluate the impact of attending clinic, and the experience of having genital examination and invasive testing or not, on psychosocial wellbeing and self-esteem.

The issue of gender concordance between participants and interviewers/researchers has been discussed in the literature [38] and the fact that our study recruiter was female may have affected recruitment and retention. A study investigating attitudes to Viagra in heterosexual Hispanic men, using telephone qualitative interviews,

For peer review only - http://bmjopen.bmj.com/site/about/guidelines.xhtml

found that changing from female to male recruiters and data collectors increased their recruitment [26]. Participants were offered the option of a male or female interviewer but may not have felt comfortable with asking for a particular interviewer, and so this may not have been sufficient to reduce the potential impact of gender discordance on acceptance of the invitation to an interview in our study. We suggest future studies involving qualitative interviews should consider the gender of recruiters and interviewers.

We received feedback from our participants that some of the wording of questions on our CASIs did not appear to be directly relevant to our study aims. Future studies should ensure that data collection measures use terms that are clear and familiar to participants, possibly through development of questionnaires with

For peer review only - http://bmjopen.bmj.com/site/about/guidelines.xhtml

focus groups. Some men in our study also saw participation as an opportunity to learn more about their own health and requested that future studies offer research participants material on health promotion, i.e. material on the infection they were diagnosed with and how to reduce risk of infection.

Although the Rosenberg Self Esteem scale and the EQ-5D-5L scales we used in this study have been validated in men and women, the panel of questions used to assess men's feelings about a diagnosis of CT or NSGI in the FU-1 CASI has not been validated in men, or for non-CT STI diagnoses. In addition, we assessed psychosocial and HRQoL impact at a single time-point, which does not measure durability of the observed decrease in self-esteem in this population, and data suggest impact of STI diagnoses may decrease over time [39].

For peer review only - http://bmjopen.bmj.com/site/about/guidelines.xhtml

Interpretation	20	Give a cautious overall interpretation of results considering objectives, limitations, multiplicity of analyses, results from similar studies, and other relevant evidence	12	In conclusion, our study shows that heterosexual men with urethral symptoms may experience negative psychosocial effects following SHC attendance. Given that incidence of NSGI is high [16] and up to 20% of men affected have recurrent symptoms [5, 6], a significant number of men may be adversely affected. Future research in this area is needed, firstly to address the high level of LTFU, and then to test the generalisability of our findings as to whether there are significant psychosocial harms of giving presumptive and non-specific diagnoses.
Generalisability	21	Discuss the generalisability (external validity) of the study results	12	Future research in this area is needed, firstly to address the high level of LTFU, and then to test the generalisability of our findings as to whether there are significant psychosocial harms of giving presumptive and non-specific diagnoses.

For peer review only - http://bmjopen.bmj.com/site/about/guidelines.xhtml

Other information				
Funding	22	Give the source of funding and the role of the funders for the present study and, if applicable, for the original study on which the present article is based	15	This study was funded by an Innovate UK (SBRI grant no. 971452) awarded to Atlas Genetics Ltd. Neither Atlas Genetics nor Innovate UK had a role in the design, collection, management, analysis, interpretation of data or decision to submit a report.

*Give information separately for cases and controls in case-control studies and, if applicable, for exposed and unexposed groups in cohort and cross-sectional studies.

Note: An Explanation and Elaboration article discusses each checklist item and gives methodological background and published examples of transparent reporting. The STROBE checklist is best used in conjunction with this article (freely available on the Web sites of PLoS Medicine at http://www.plosmedicine.org/, Annals of Internal Medicine at http://www.annals.org/, and Epidemiology at http://www.epidem.com/). Information on the STROBE Initiative is available at www.strobe-statement.org.

For peer review only

For peer review only - http://bmjopen.bmj.com/site/about/guidelines.xhtml

Health related quality of life and psychosocial impacts of a diagnosis of non-specific genital infection in symptomatic heterosexual men attending UK Sexual Health clinics: a feasibility study.

Journal:	*BMJ Open*
Manuscript ID	bmjopen-2017-018213.R2
Article Type:	Research
Date Submitted by the Author:	11-Apr-2018
Complete List of Authors:	Hill-Tout, Rachel; St George's NHS Foundation Trust, Genitourinary Medicine & HIV; Public Health England, HIV & STI<br>Harding- Esch, Emma; St George's University of London, Applied Diagnostic Research and Evaluation Unit, Institute for Infection & Immunity; Public Health England, HIV & STI<br>Pacho, Agata; St George's University of London, Applied Diagnostic Research and Evaluation Unit, Institute for Infection & Immunity<br>Furegato, Martina; St George's University of London, Applied Diagnostic Research and Evaluation Unit, Institute for Infection & Immunity; Public Health England, HIV & STI<br>Fuller, Sebastian; St George's University of London, Applied Diagnostic Research and Evaluation Unit, Institute for Infection & Immunity<br>Sadiq, S. Tariq; St George's University of London, Applied Diagnostic Research and Evaluation Unit, Institute for Infection and Immunity Research Institute; Public Health England, HIV & STI
**Primary Subject Heading**:	Sexual health
Secondary Subject Heading:	Patient-centred medicine, Qualitative research, Sociology, Sexual health
Keywords:	GENITOURINARY MEDICINE, QUALITATIVE RESEARCH, Genitourinary medicine < INTERNAL MEDICINE

SCHOLARONE™
Manuscripts

Health related quality of life and psychosocial impacts of a diagnosis of non-specific genital infection in symptomatic heterosexual men attending UK Sexual Health clinics: a feasibility study.

Rachel Hill-Tout[1,3], Emma M. Harding-Esch[2, 3], Agata Pacho[2], Martina Furegato[2, 3], Sebastian S. Fuller*[†2], S Tariq Sadiq[†1, 2, 3]
* Corresponding author
[†] Joint senior authors

1. St Georges University Hospitals NHS Foundation Trust
   Blackshaw Rd, London SW17 0QT UK

2. Applied Diagnostic Research and Evaluation Unit
   Institute for Infection & Immunity
   St George's, University of London
   London, SW17 0RE, UK

3. Public Health England
   61 Colindale Avenue
   London, NW9 5EQ UK

Rachel Hill-Tout
Specialist Registrar HIV/GUM
orcid.org/0000-0003-1892-8598
r.hill-tout@nhs.net

Emma M. Harding-Esch
ADREU Epidemiology Lead & Programme Manager
orcid.org/0000-0002-1432-8109

Agata Pacho
ADREU Research Assistant
orcid.org/0000-0002-0653-5095

Martina Furegato
Statistician - HIV & STI department
orcid.org/0000-0003-2168-8396

Sebastian S. Fuller
ADREU Social Science Lead
orcid.org/0000-0002-9284-6630
sfuller@sgul.ac.uk

S Tariq Sadiq
Reader in HIV and Sexual Health Medicine, Consultant Physician / ADREU Lead
orcid.org/0000-0002-0608-381

For peer review only - http://bmjopen.bmj.com/site/about/guidelines.xhtml

ABSTRACT

Introduction:

Non-specific genital infection (NSGI; non-*Chlamydia trachomatis* (CT), non-*Neisseria gonorrhoeae* (NG) associated urethritis) is a common diagnosis in symptomatic heterosexual men attending UK Sexual Health Clinics (SHCs) but little is known about the psychosocial impact of this diagnosis.

Methods:

We conducted an observational study among symptomatic heterosexual men attending SHCs to evaluate the psychosocial impact of an NSGI diagnosis compared to a diagnosis of CT, NG or no abnormalities detected (NAD) focussing on the feasibility of our study methodology. Participants completed a Computer Assisted Self Interview (CASI) including two validated measures of psychosocial impact: the EQ-5D-5L health-related quality-of-life (HRQoL) and Rosenberg Self-Esteem scales, before diagnostic testing and two weeks after receiving test results (FU-1), and a qualitative interview. We compared scores between diagnostic groups using paired t-tests, qualitative data were analysed thematically, and feasibility was assessed by process analysis.

Results:

60 men completed the baseline CASI (75% response rate). 46 (76.6%) were eligible for follow-up; 11/46 (23.9%) completed the follow-up CASI, and 3/11 (27.3%) completed the qualitative interview. 81.7% of all participants left CASI feedback at baseline: 73.5% reported the questionnaire as ‘fine’ or ‘very good’. Qualitative interview participants reported the study was acceptable. Compared to baseline, among patients completing FU-1, only patients with a diagnosis of NSGI ($p<0.05$) or CT ($p<0.05$) showed increased EQ-5D-5L Index, whereas patients with a diagnosis of NSGI ($P=0.05$) showed decreased mean Rosenberg Self-Esteem Scale score.

Conclusions:

Although most participants indicated study acceptability at baseline, and we employed measures to increase retention (CASI questionnaires, reminder messages, a focus on men’s health), we experienced high loss to follow-up. We found that heterosexual men attending SHCs with symptoms of urethritis experience both positive and negative psychosocial impacts following their clinic attendance, which warrants further investigation.

Article Summary

Strengths and Limitations of this study

- This study presents the first data investigating the psychosocial impacts of the common diagnosis of non-specific genital infection in heterosexual men attending sexual health clinics.
- This study used both validated scales and qualitative interviews to assess psychosocial impact.
- We used computer assisted self-interviewing to collect data on psychosocial impact as this has been shown to improve data quality in sensitive topics.

For peer review only - http://bmjopen.bmj.com/site/about/guidelines.xhtml

- The study was small and was conducted in two London sexual health clinics, which may limit the generalizability of our results.
- There was high loss to follow-up after baseline, which may have biased our results.

INTRODUCTION

Non-specific Genital Infection (NSGI) or, non-*Chlamydia trachomatis* (CT), non-*Neisseria gonorrhoeae* (NG) associated urethritis (NGU), is the third most frequently diagnosed condition in heterosexual men attending sexual health clinics (SHCs) in the United Kingdom (UK), with 30,381 cases/year compared to CT (42,483/year) and first presentation of genital warts (32,656/year) [1]. NSGI is a diagnosis of exclusion; men with urethral symptoms are screened with urethral microscopy in SHCs, and if this demonstrates urethritis with no gonococci, a provisional diagnosis of NGU is made [2]. If urinary Nucleic Acid Amplification Tests (NAATs) sent for NG and CT are both negative, the diagnosis is recorded as NSGI.

Although a common condition, NSGI remains poorly understood. NSGI can be caused by a variety of sexually transmitted infections (STIs), including *Mycoplasma genitalium* (MG) and *Trichomonas vaginalis* (TV), which are not routinely tested for in SHCs, and many cases have an uncertain aetiology [2]. Microscopy, on which the diagnosis rests, has poor specificity for known causes of urethritis [3] and is subject to considerable observer variation [4]. National guidelines recommend that men diagnosed with NGU are informed they may have an STI, given same day empirical antibiotics, advised their partners should attend for STI testing and empirical treatment, and to abstain from sex until treatment is completed [2]. Men usually receive CT/NG NAAT results within 8 days [5]. Following first-line antibiotics for NSGI, 10%–20% of men return because of persistent symptoms [6,7].

Men who undergo STI testing, and are diagnosed with an STI report negative impacts on their psychosocial wellbeing, including feelings of stigma [8–10], however, to our knowledge, there are no published data on the psychosocial impact of NSGI diagnosis among men. Stigma has been shown in men to be associated with a delay in testing for STIs, and decreased willingness to notify their casual partners of their STI diagnosis [11]. Studies on psychosocial impact, partner notification and quality of life impact have predominantly been carried out in women [9,12–14], with few studies recruiting men. A greater understanding of the degree and nature of psychosocial impacts of a diagnosis of an STI, or a 'presumed STI' such as NSGI, in men, is important to optimise interventions, including partner notification and risk-behaviour modification, which aim to reduce the negative impact of STIs, such as complications of infection.

Men are reportedly poor users of health services, largely due to culturally dominant ideals of masculinity that equate illness with weakness, which may impede care-seeking [15–18]. In sexual healthcare, there has traditionally been a focus on female reproductive health [19], and this may further discourage male attendance [19,20]. However, men are willing to respond openly to questions about their sexual health, particularly when enquiries are focused specifically on men's needs [19,21].

For peer review only - http://bmjopen.bmj.com/site/about/guidelines.xhtml

We decided to focus on heterosexual men in this study as there are important differences between men who have sex with men (MSM) and men who have sex exclusively with women (MSEW) in their access of SHC services, reported sexual health risks, their own perception of risk of STIs, and sexual health and wellbeing outcomes [22], suggesting that their experience of diagnosis of a genital infection may also be different. Due to these important differences between MSM and MSEW, we decided that psychosocial impacts of an STI diagnosis in MSM merited studying separately.

Because of a lack of models to measure the psychosocial impact of an NSGI diagnosis among men, and the potential difficulty in recruiting and retaining this group in sexual health studies, we designed a small-scale longitudinal mixed-method feasibility study to inform on our research methods and better understand the psychosocial and health related quality of life (HRQoL) status of symptomatic heterosexual men before and after receiving a diagnosis of NSGI, CT, NG or “nothing abnormal detected” (NAD).

METHODS

Study Design

We conducted an observational, longitudinal study in symptomatic heterosexual men using patient completed questionnaires assessing HRQoL and psychosocial status before and after receiving STI test results, and individual qualitative interviews to evaluate experiences at the SHC and acceptability of study methods.

Eligibility criteria were: heterosexual men presenting with urethral symptoms (dysuria, discharge or urethral discomfort), aged ≥16 years at either of two participating London SHCs between September - October 2016. We excluded men who were asymptomatic, not available for follow-up, and who were unable to understand English.

Following informed consent, participants were invited to complete a baseline computer assisted self-interview (CASI) assessing psychosocial and HRQoL status in clinic prior to consultation, a follow-up CASI after receipt of all test results 1-2 weeks after baseline (Follow-up 1 (FU-1)) and a qualitative interview 2-4 weeks after baseline (Follow-up 2 (FU-2)).

The participant pathway is detailed in Figure 1. After completion of the baseline CASI, any participants diagnosed with an STI other than CT, NG or NSGI, or who did not have both urethral microscopy and urinary NAATs, were withdrawn from the study. Once participants were notified of their NAAT CT/NG results by the SHC (phone call/ text message), the research team texted participants an electronic link to the FU-1 CASI.

CASIs were optimised for web-enabled devices including smartphones. We offered participants the choice to complete CASIs using their own device or a study tablet-device and use of a private room in clinic. Study staff sent ≤2 text message reminders for each follow-up. Participants who did not complete FU-1 were considered ineligible for FU-2. Participants received gift voucher reimbursements: £5 (baseline), £10 (FU-1), £20 (FU-2).

For peer review only - http://bmjopen.bmj.com/site/about/guidelines.xhtml

We categorised participants into four diagnostic groups based on microscopy and NAAT results: NSGI (NGU diagnosis on urethral microscopy and CT/NG NAATs negative); NG (NG on urethral microscopy and/or positive NAAT); CT (positive NAAT); NAD (no urethritis on microscopy and CT/NG NAATs negative).

Questionnaire design

At both baseline and FU-1 CASIs, the EQ-5D-5L, a generic measure of HRQoL [23], and the Rosenberg Self-Esteem Scale (RSES) [24], a measure of potential stigmatising effects of screening for STIs, were used. The EQ-5D-5L is the preferred utility measure recommended by the UK National Institute for Health and Clinical Excellence [22] and has five dimensions: mobility, self-care, usual activities, pain/discomfort and anxiety/depression. Weighted UK preference values are linked to the self-reported health state scores for a 0–1 Index value, where 0 is death and 1 is perfect health. The RSES is widely used in social science research to measure self esteem; it uses a scale of 0-30 where a score less than 15 may indicate low self esteem, 15-25 normal self-esteem and 25-30 high self-esteem.

In addition, the baseline CASI included the Multidimensional Scale of Perceived Social Support (MSPSS) [25] to assess coping resources. The MSPSS is widely used to assess perceived social support and consists of three domains: family, friends, and significant others. Mean MSPSS scores from 1- 2.9 may be considered low support; 3- 5 moderate support and 5.1- 7 high support [26].

We collected data on sociodemographics in the baseline CASI and current relationship status in both the baseline and FU-1 CASI. In the FU-1 CASI we included a panel of questions previously developed to assess impact of CT diagnosis in women [13], which we modified for use in men by removing a pregnancy-related question. Participants were asked to indicate acceptability of the CASIs (free-text response) at baseline and FU-1. (Supplementary Appendices 1., 2. and 3.)

Patient and Public Involvement

There was no formal patient and public involvement informing the research question or study procedures. Clinician experiences with patients diagnosed with non-specific genital infection informed the need for investigation of the potential harms resulting from this diagnosis. The results have been disseminated via the study groups' community advisory group (formed after the research was planned and implemented).

Sample size

As this was a feasibility study with no precedent, no formal sample size could be calculated. We planned to approach 100 patients at baseline as a convenience target, and assuming approximately 50% refusal, we expected 50-60 patients to be enrolled and estimated ~50% attrition at each follow-up.

Outcome measures

For peer review only - http://bmjopen.bmj.com/site/about/guidelines.xhtml

Primary outcome: Qualitative and quantitative process data on recruitment and retention rates at baseline and follow-up to inform on feasibility of study design. Evaluation of potential relevance of questions validated in other populations for men diagnosed with NSGI.

Secondary outcomes:

Percentage change from baseline to FU-1 in mean EQ-5D-5L Index value, and mean RSES value in men diagnosed with CT, NG, NSGI or NAD.

We did not have prior expectations about different impacts of these diagnoses, and as there are no studies comparing the impact of CT to NG or NSGI in men, this was exploratory. We were interested in investigating whether NSGI was perceived as a benign diagnosis or whether it elicited negative psychosocial impacts which have been reported in men diagnosed with STIs such as CT [10].

Data analysis

Questionnaire data:

For all eligible men, RSES score, MSPSS score, and EQ-5D-5L Index value were calculated according to published methods [24][23][25][27].

For men in each diagnostic category, paired t-tests were used to compare mean values for the EQ-5D-5L Index, RSES and MSPSS at baseline, and to compare differences in mean values for EQ-5D-5L Index and RSES score, from baseline to follow-up.

To test for non-response bias, we compared sociodemographic data of those who completed both baseline and follow-up to those who were lost to follow-up (LTFU), using Pearson's Chi^2 tests and Kruskal-Wallis tests. Statistical significance was set at $p<0.05$. Statistical analysis was carried out using STATA version 13.1 (StataCorp, 2015).

Qualitative data: Audio recordings were transcribed and transcripts checked for accuracy against the recording. Cleaned transcripts were then imported into NVivo (Version 10, 2012) for analysis. Two researchers assigned themes to text independently, with discrepancies resolved by consensus before reporting final themes. All names of participants presented here are pseudonyms.

RESULTS

We approached 80 patients, of whom 60 (75%) enrolled and completed the baseline CASI. 46 (76.6%) were eligible for follow-up; of these, 11 (23.9%) completed the FU-1 CASI, and 3 of these (27.3%) completed FU-2 (Figure 2.) Final diagnoses among all eligible participants (n=46) were: CT, 7 (15.2%); NG, 2 (4.3%); NSGI, 23 (50%); NAD, 14 (30.4%), and among participants who completed FU-1 (n=11) were CT, 4 (36.4%); NSGI, 4 (36.4%); NAD, 3 (27.3%). None of the participants diagnosed with NG completed FU-1. Two participants with

For peer review only - http://bmjopen.bmj.com/site/about/guidelines.xhtml

NSGI and one with NAD completed FU-2. Clinical records indicated that all participants were notified of their CT/NG NAAT diagnosis within 7 days of completing baseline.

Men who reported urethral symptoms but had no abnormalities on urethral sample testing and negative CT and NG results (categorised as NAD) had a variety of non-STI causes for their symptoms diagnosed, including irritant or *Candida* balanitis, lower urinary tract symptoms which were likely prostatic in origin, non-infectious genital pain syndromes, and symptoms secondary to urethral trauma.

Participants' sociodemographic factors at baseline are presented in Table 1. Most were between 25-35 years old (60.9%), and over half reported their ethnicity as non-White (52.2%). 80.4% indicated they were not in a relationship and many had previously been diagnosed with STIs (30.4% NGU, 28.3% CT, 13.0% genital herpes or warts and 8.7% NG).

Pearson's Chi^2 tests and Kruskal-Wallis test results for non-response bias were not statistically significant so we assumed data were missing at random. We compared participants who completed FU-1 to those LTFU by employment, age, education, ethnicity, and current relationship status; none of these comparisons showed significant differences.

Process evaluation

All patients who completed the baseline CASI did so using the Study tablet device in a private room in the clinic. Participants completed the baseline CASI in about 5 minutes. All participants completed the follow-up CASI at home on a participant-owned device.

CASI feedback suggested good acceptability: of the total 60 who completed the Baseline CASI, 81.7% left feedback, of whom 73.5% reported that the questionnaire was "fine" or "very good" in a free-text box at the end of the questionnaire.

Participants suggested improvements to the study during qualitative interviews: to use the research as a tool for health promotion and to make CASI questions more clearly relevant to sexual health. Insight into LTFU was provided by one participant, who said he might not have continued with the study if he had received a positive STI diagnosis: "*I reckon if my tests had not come back the way I wanted them to, I probably wouldn't have done this last [follow-up]. … I might've still said, 'Yes, let's do it.' I don't know but I… Just sitting here, thinking about it now, I probably wouldn't complete the rest of it."* (Lucas, NAD diagnosis).

CASI analysis

For peer review only - http://bmjopen.bmj.com/site/about/guidelines.xhtml

Distributions of participants' psychosocial scores at baseline by final diagnosis are shown in Table 2. At baseline (Table 2a) participants ultimately diagnosed with NG had significantly lower (p≤0.050) mean MSPSS scores (indicating lower social support), compared to men ultimately diagnosed with NSGI or NAD, as well as lower scores as compared to those diagnosed with CT (p=0.059). Participants with NG also had lower mean RSES scores (indicating lower self-esteem) at baseline as compared to those with NSGI (p=0.035). EQ-5D-5L Index value at baseline did not vary significantly between diagnosis groups.

We found men with NSGI and CT showed a significant increase in mean EQ-5D-5L Index value (indicating better HRQoL) between baseline and FU-1 (+17% (p=0.006) and +219% (p=0.005), respectively) (Table 2b). There was no change in mean EQ-5D-5L Index value for men diagnosed with NAD. In contrast, we found decreases in mean RSES scores from baseline to FU-1 in men with NSGI (-30%, p=0.05).

Participants who reported that they had been diagnosed with an infection (n=5, 4 with a final diagnosis of CT and 1 with NSGI) completed a panel of questions in the FU-1 CASI to assess their feelings about their diagnosis. All participants reported that they were 'not worried as it is curable' and did not think the diagnosis would change how their friends thought about them, but only one participant (diagnosed with CT) reported he had talked to his friends about his diagnosis. All participants reported concern that they might get the infection again, and the majority of those with a CT diagnosis (75%) reported concern about exposure to other STIs, feeling "dirty" and embarrassed, being scared to tell their partners about their diagnosis and worried that their partners thought they had been unfaithful.

Qualitative analysis

In interviews, participants compared different STIs as a way to contextualise their level of concern about their own diagnosis. For one participant, his main concern was the possibility of being diagnosed with HIV: *"Look, to be honest with you, the key one everyone really wants to know about is HIV … all the other ones are treatable quite easily…"* (Lucas, NAD diagnosis). There were several points within the interviews where participants expressed concern at being diagnosed with an infection they did not know about, indicating that non-specific diagnoses may cause negative impact: *"…to be honest, I'm a little bit worried about this because there is no name of the new bacteria, so we don't know if it's dangerous or not..."* (Liam, NSGI diagnosis).

Among the two interview participants diagnosed with NSGI, one described anxiety following his presumptive CT diagnosis in clinic. He was in a long-term monogamous relationship and so was not expecting an STI diagnosis: *"My heart's racing. I'm thinking, 'but how, I can't.' … I just had a lot of thoughts in my head. … And I told [the doctor], 'I can't. I don't know how I would have this.' He's saying, 'Well, whatever you have, it's sexually transmitted.' I said, 'But it can't be.' So of course I start getting things in my head."* (Jacob, NSGI diagnosis). (Supplementary Appendix 4.)

DISCUSSION

For peer review only - http://bmjopen.bmj.com/site/about/guidelines.xhtml

In this small observational feasibility study we assessed patient reported self-esteem and HRQoL using validated scales in heterosexual men presenting to SHCs with urethral symptoms, before undergoing STI testing, and after receiving all test results. This is the first study to investigate the HRQoL and psychosocial impact of a curable STI diagnosis in symptomatic men attending SHCs, using validated scales, and is one of the few available with data on STIs and EQ-5D-5L [28].

We found increased EQ-5D-5L Index value (indicating increased HRQoL) in men diagnosed with NSGI or CT from baseline to FU-1. Published studies have reported that some men diagnosed with CT reported a lack of concern regarding the diagnosis as they perceive CT as a relatively minor infection [8,10,14,29] This was supported by one of our qualitative interview participants. The increases we found in the EQ-5D-5L Index value may reflect decreases in the anxiety and pain/discomfort domains of the scale from just prior to consultation at baseline to receipt of all test results in FU-1. Shoveller *et al.* reported that the majority of men diagnosed with an STI in SHCs had reported feeling anxious waiting for potentially bad news [30], and so this finding may reflect that men found relief following treatment and explanation of their symptoms, and receiving negative results for more serious infections such as HIV. Due to our small sample size we were unable to find significant associations between participant satisfaction with their clinic visit and relief from symptoms and infection status in our CASI FU-1 data.

Furthermore, in men diagnosed with NSGI, we found decreased mean RSES score (indicating decreased self-esteem) from baseline to approximately one week after receiving all STI test results. These findings support data from previous qualitative studies where men diagnosed with an STI in an SHC reported negative psychosocial impacts including stigma; anxiety; shame; isolation; concerns regarding relationships; a loss of social status; vulnerability; a lack of privacy and fear of STI testing, particularly urethral swab testing [8,10,30–32]. We did not find a decrease in self-esteem in men diagnosed with CT which might suggest that NSGI is perceived differently to CT. In our qualitative interviews participants with NSGI reported concerns regarding the uncertainty of the diagnosis, and fear of the impact of a possible STI diagnosis on their relationships; these factors may be important in the observed decreased self-esteem in these men.

Limitations

Our choice of HRQoL tool, the EQ-5D-5L, may not be sensitive enough to detect impacts caused by the diagnosis of CT, NG or NSGI, which are unlikely to cause problems in at least two of the five domains: mobility, and self-care. Studies investigating HRQoL and sexual health have found few significant differences with comparator groups using generic QoL instruments alone [33]. STI-specific HRQoL and psychosocial impact tools have been developed for genital herpes and genital warts [34–36] and several studies have now also combined both EQ-5D-5L and STI-specific tools [37–40].

Our study did not include asymptomatic men, hence we cannot exclude the possibility that the negative impact on self-esteem observed with men was not related to being symptomatic or undergoing genital

For peer review only - http://bmjopen.bmj.com/site/about/guidelines.xhtml

examination and a urethral swab [10,30,32]. We suggest that future studies consider including both symptomatic and asymptomatic men to better evaluate the impact of the experience of having symptoms, genital examination and invasive testing on psychosocial wellbeing and self-esteem.

Although the RSES and the EQ-5D-5L scales we used in this study have been validated in men and women, the panel of questions used to assess men's feelings about a diagnosis of CT or NSGI in the FU-1 CASI has not been validated in men, or for non-CT STI diagnoses. In addition, we assessed psychosocial and HRQoL impact at a single time-point, which does not measure durability of the observed decrease in self-esteem in this population, and data suggest psychosocial impact of STI diagnoses may decrease over time [41].

We received feedback from our participants that some of the wording of questions on our CASIs did not appear to be directly relevant to our study aims. Future studies should ensure that data collection measures use terms that are clear and familiar to participants, possibly through development of questionnaires with focus groups. Some participants requested that future studies offer research participants material on health promotion, i.e. material on the infection they were diagnosed with and how to reduce risk of infection.

Despite concerns that the target group of heterosexual men attending SHCs would be difficult to recruit [42], we found 75% of men were happy to enrol in this study. This should encourage researchers to investigate views from this group regarding STI diagnosis and screening. A study investigating the psychosocial experiences of patients attending a clinic, which recruited men and women diagnosed with an STI from an SHC, reported a recruitment rate of 33% [31] and involved ten interviews lasting between 20-45 minutes each. Our higher baseline recruitment rate may be due to the use of CASI interviewing, fewer interviews, or because patients were recruited before they had any diagnosis made.

One of the difficulties associated with conducting longitudinal studies is attrition. In our study, of those eligible for follow-up, 24 % overall completed the follow-up CASI, 25% of those with a diagnosis of NSGI, CT or NG, and 21% of those with NAD. A longitudinal questionnaire study investigating community screening for CT reported follow-up rates of over 60% , however, eligibility for follow-up excluded those with positive STI test results, and 60% of the sample was female, reflecting the higher response rate amongst women [12]. Our study's qualitative component had a follow-up rate of 27% overall, 25% in those who had any positive diagnosis, and 33% in those with NAD. By comparison, a study including men, investigating patient views on CT screening recruited 6.6% of those who completed the baseline questionnaire to a qualitative interview, which was below their target of 10% [43].

We embedded several mechanisms in our study to improve participant acceptability and retention. Privacy when completing sensitive questions has been shown to improve participant acceptability for revealing potentially stigmatising information [44]; we used a web-based CASI and the option for participants to self-complete the questionnaire in a private room in the clinic. Potential research participants, and men

For peer review only - http://bmjopen.bmj.com/site/about/guidelines.xhtml

specifically, are more likely to participate in research that represents their concerns [19,21,45]; eligibility for this study was restricted to those patients who were likely to be diagnosed with an STI, and focused on the impact of those diagnoses. In addition, reimbursements have been shown to play a part in increasing enrolment and retention in research [45,46]; we reimbursed participants in increasing amounts to reflect the time and effort we estimated were necessary to complete the research. Despite these mechanisms, and positive participant feedback on the baseline CASI and in our qualitative interviews, we experienced high LTFU. High LTFU resulted in low numbers of respondents within diagnostic categories, which meant we were unable to adjust our analysis for common confounders, such as education and socioeconomic status, known to be associated with gonorrhoea [47,48]. This may be a source of bias in our results. We did not find significant differences between groups in terms of non-response bias and other characteristics, however this is not surprising as the power to detect differences between groups is greatly affected by sample size and our study included a small number of participants. Recruiting larger numbers of participants in future studies will increase the statistical power to detect any important differences between groups.

We explored potential correlates and causes for LTFU within our dataset. Men's socioeconomic status and age has been seen to affect both their knowledge of and interaction with health services [20]. However, we did not find significant differences in these factors between those LTFU and those who completed FU-1. One participant indicated in qualitative interview that if he had received a positive STI diagnosis it was unlikely that he would have agreed to continue with the research. This may give insight into why no patients receiving a positive CT or NG diagnosis agreed to FU-2.

We had planned to further explore study acceptability by inviting participants who failed to complete FU-1 to qualitative interviews; however, we followed Research Ethics Committee recommendations to consider that these participants had passively withdrawn from the study and refrained from inviting them to FU-2. Yet, qualitative and quantitative methods of data collection are viewed differently by respondents [49,50]. One participant described the difference between his experiences completing the survey and participating in an interview:

"…when I'm sitting here with you, obviously it's just me and you and you've got my hundred percent full attention, haven't you? But then when I'm sitting at my desk at work, trying to complete a survey and there's other things going on and people asking me questions and 'can you do this, can do you that'; it's different." (Lucas, NAD diagnosis)

Although we cannot know if participants who did not complete FU-1 would have participated in a qualitative interview if they had been invited, one participant left feedback on the baseline CASI indicating he would have preferred to elaborate on his answers, and several participants informally conveyed this to the researcher at baseline. Research has shown that one unique benefit of qualitative interviews is that it provides participants with an opportunity to expand or contextualise issues [49,50]. In addition, the insights we gained from the

For peer review only - http://bmjopen.bmj.com/site/about/guidelines.xhtml

limited qualitative interviews we conducted indicate the potential usefulness of this method to understand nuances of acceptability that may lead to reduced LTFU. We therefore recommend that future feasibility studies include qualitative components as a means to allow participants, including those who fail to complete survey questionnaires, to provide important feedback on study methods. Future studies could also consider offering a qualitative interview at the point of recruitment, not only at follow-up, to widen the possibility of a qualitative exploration among participants who do not find the CASI questionnaire acceptable.

The issue of gender concordance between participants and interviewers/researchers has been discussed in the literature [51] and the fact that our study recruiter was female may have affected recruitment and retention. A study investigating attitudes to Viagra in heterosexual Hispanic men, using telephone qualitative interviews, found that changing from female to male recruiters and data collectors increased their recruitment [42]. Participants in our study were offered the option of a male or female interviewer but may not have felt comfortable with asking for a particular interviewer, and so this may not have been sufficient to reduce the potential impact of gender discordance on acceptance of the invitation to an interview in our study. We suggest future studies involving qualitative interviews should consider the gender of recruiters and interviewers.

Conclusions

Our study found that heterosexual men with urethral symptoms may experience a range of psychosocial and HRQoL impacts following SHC attendance and STI testing. More work is needed to investigate the most appropriate methodology for investigating sexual health and QoL, particularly around choosing between condition specific and generic measures, and reducing loss to follow-up in longitudinal studies. Future research in this area is needed to test the generalizability of our findings as to whether there are significant psychosocial harms of giving presumptive and non-specific diagnoses.

TABLES AND FIGURES

Figure 1. Patient Pathway
Figure 2. Enrolment and Follow-up Chart
Table 1. Sociodemographic characteristics of enrolled participants at Baseline by final diagnosis.
Table 2. (a, b) Distribution of psychosocial scores by final diagnosis.

Supplementary Appendices

Appendix 1. Baseline CASI Questionnaire
Appendix 2. Follow-up 1 CASI Questionnaire
Appendix 3. Individual in-depth Interview Topic Guide
Appendix 4. Full list of themes for Qualitative Interview (Follow-up 2)

For peer review only - http://bmjopen.bmj.com/site/about/guidelines.xhtml

Table 1. **Sociodemographic characteristics of enrolled participants at Baseline by final diagnosis**

	CT n (%)	NG n (%)	NSGI n (%)	NAD n (%)	Total n
**Age**					
<24	5 (71.43%)	1 (50%)	5 (21.74%)	3 (21.43%)	14
25-35	2 (28.57%)	1 (50%)	17 (73.91%)	8 (57.14%)	28
>35	0 (0%)	0 (0%)	1 (4.35%)	3 (21.43%)	4
**Ethnicity**					
White	5 (71.43%)	0 (0%)	10 (43.48%)	6 (42.86%)	21
Asian	0 (0%)	1 (50%)	2 (8.7%)	2 (14.29%)	5
Black	1 (14.29%)	1 (50%)	10 (43.48%)	2 (14.29%)	14
Other	1 (14.29%)	0 (0%)	1 (4.35%)	3 (21.43%)	5
Prefer not to say	0 (0%)	0 (0%)	0 (0%)	1 (7.14%)	1
**Age at leaving full-time education**					
16 years or less	0 (0%)	0 (0%)	1 (4.35%)	3 (21.43%)	4
17 or 18 years	0 (0%)	0 (0%)	6 (26.09%)	0 (0%)	6
19 years or over	4 (57.14%)	0 (0%)	14 (60.87%)	10 (71.43%)	28
Still in full-time education	3 (42.86%)	2 (100%)	2 (8.7%)	1 (7.14%)	8
**Highest educational achievement**					
No qualifications/No formal qualifications	1 (14.29%)	0 (0%)	1 (4.35%)	0 (0%)	2
1-5 GCSEs	0 (0%)	0 (0%)	3 (13.04%)	2 (14.29%)	5
2 or more A-levels or equivalent	1 (14.29%)	1 (50%)	4 (17.39%)	2 (14.29%)	8
Bachelor’s degree or equivalent and higher	5 (71.43%)	1 (50%)	13 (56.52%)	8 (57.14%)	27
Other	0 (0%)	0 (0%)	2 (8.7%)	2 (14.29%)	4
**Do you have any long-standing condition?**					
Yes	1 (14.29%)	0 (0%)	1 (4.35%)	2 (14.29%)	4
No	6 (85.71%)	2 (100%)	22 (95.65%)	12 (85.71%)	42
**Previously diagnosed with CT?**					
Yes	4 (57.14%)	1 (50%)	7 (30.43%)	1 (7.14%)	13
No	3 (42.86%)	1 (50%)	16 (69.57%)	13 (92.86%)	33
**Previously diagnosed with NG?**					
Yes	0 (0%)	2 (100%)	2 (8.7%)	0 (0%)	4
No	7 (100%)	0 (0%)	21 (91.3%)	14 (100%)	42
**Previously diagnosed with NGU?**					
Yes	2 (28.57%)	0 (0%)	9 (39.13%)	3 (21.43%)	14
No	5 (71.43%)	2 (100%)	14 (60.87%)	11 (78.57%)	32

For peer review only - http://bmjopen.bmj.com/site/about/guidelines.xhtml

Previously diagnosed with herpes/warts?					
Yes	3 (42.86%)	0 (0%)	1 (4.35%)	2 (14.29%)	6
No	4 (57.14%)	2 (100%)	22 (95.65%)	12 (85.71%)	40
**Relationship status**					
Married and living with wife	0 (0%)	0 (0%)	1 (4.35%)	2 (14.29%)	3
Cohabiting but not married	1 (14.29%)	0 (0%)	2 (8.7%)	2 (14.29%)	5
Separated, divorced, or widowed	0 (0%)	0 (0%)	1 (4.35%)	0 (0%)	1
Single (that is never married)	6 (85.71%)	2 (100%)	19 (82.61%)	9 (64.29%)	36
Prefer not to say	0 (0%)	0 (0%)	0 (0%)	1 (7.14%)	1
**Break up in the last 30 days**					
Yes	1 (14.29%)	0 (0%)	3 (13.04%)	5 (35.71%)	9
No	6 (85.71%)	2 (100%)	20 (86.96%)	9 (64.29%)	37

For peer review only

For peer review only - http://bmjopen.bmj.com/site/about/guidelines.xhtml

Table 2 Distribution of psychosocial scores by final diagnosis

Tab 2.a Distribution of psychosocial scores by final diagnosis within the baseline group and results from student's t-test

		Score	95% CI	p-value NSGI vs. CT	p-value NSGI vs. NAD	p-value CT vs. NAD	p-value NG vs. CT	p-value NG vs. NSGI	p-value NG vs. NAD
**EQ-ED-5L (index value)**	**NG**	0.46	0.28-0.64	0.238	0.367	0.708	0.700	0.794	0.882
	**NSGI**	0.59	0.46-0.72						
	**CT**	0.44	0.19-0.69						
	**NAD**	0.49	0.32-0.67						
**Rosenberg Self Esteem Scale**	**NG**	15.5	0.0-72.7	0.35 8	0.816	0.354	0.09	**0.035**	0.071
	**NSGI**	22.0	20.0-24.0						
	**CT**	20.4	17.5-23.4						
	**NAD**	22.4	19.6-25.6						
**Multidimensional scale of social support**	**NG**	2.5	2.0-3.0	0.583	0.610	0.918	**0.059**	**0.016**	**0.040**
	**NSGI**	3.1	2.9-3.2						
	**CT**	3.2	2.8-3.6						
	**NAD**	3.2	2.9-3.4						

Tab 2.b Distribution of psychosocial scores by final diagnosis within the FU group and within the Baseline group restricted to those with a FU record comparing Baseline and FU scores

		Baseline*		FU-1			
		Score	95% CI	Score	95% CI	% difference of score Baseline* and FU-1	p-value Baseline* vs. FU- 1
**EQ-ED-5L (index value)**	**NSGI**	0.82	0.79-0.86	0.96	0.85-1.07	17%	**0.006**
	**CT**	0.27	0.14-0.39	0.85	0.56-1.14	219%	**0.005**
	**NAD**	0.59	0.16-1.00	0.75	0.25-1.24	27%	0.658
**Rosenberg Self Esteem Scale**	**NSGI**	22.5	14.6-30.3	15.7	7.81-23.69	-30%	**0.050**
	**CT**	20.7	12.81-28.69	20.0	5.71-34.29	-3%	**0.444**
	**NAD**	22.7	8.11-	19.3	15.5-	-15%	**0.197**

For peer review only

For peer review only - http://bmjopen.bmj.com/site/about/guidelines.xhtml

			37.22		23.1		

*restricted to patients who completed FU-1: no patients diagnosed with NG were present at FU-1.

<u>Abbreviations used in Tables 2 a and b</u>

CT- *Chlamydia trachomatis*
NG- *Neisseria gonorrhoeae*
NSGI- Non specific genital infection
NAD- No abnormality detected
FU- Follow-up

Ethical Approval

This study was approved by the London Bridge Research Ethics Committee (REC reference 16/LO/0955).

Acknowledgements

Thank you to the participants who took part in the study, our collaborators and the steering committee for their advice, including: Atlas Genetics, who were awarded the Innovate UK SBRI grant; Aquarius Population Health; Scientific Steering Committee (Chris Price, Kate Folkard, David Livermore), and the clinical leads at participating clinics (Richard Lau, Paul Lister).

Contributor Statement

STS conceived of the Study. RHT, SSF and EHE initiated the study design. AP, SSF, RHT and EHE implemented the study. MF and EHE provided statistical oversight. RHT wrote the initial draft of the manuscript. All authors contributed to and approved the final manuscript.

Competing Interests

The Applied Diagnostic Research and Evaluation Unit at St George's, University of London (STS, EHE, SF, AP), receives funding from the National Institute of Health Research (NIHR) i4i Programme (grant number II-LB-0214-20005), Atlas Genetics, Alere, Hologic Cepheid, SpeeDx, Sekisui and Becton Dickinson to develop Point of Care Tests for STIs. SF and EHE are members of Becton Dickenson's Advisory Board for the provision of sexual health in the UK. MF and RHT have no competing interests. The views expressed are those of the authors and not necessarily those of the NIHR, the NHS or the Department of Health.

Funding

This study was funded by an Innovate UK (SBRI grant no. 971452) awarded to Atlas Genetics Ltd. Neither Atlas Genetics nor Innovate UK had a role in the design, collection, management, analysis, interpretation of data or decision to submit a report.

Data Sharing Statement

Due to ethical concerns regarding the sensitive nature of the research, transcripts of qualitative interviews are not available for data sharing. All other data supporting this study are provided as supplementary information accompanying this paper.

REFERENCES

1 Table 4: All STI diagnoses & services by gender & sexual risk, 2010 - 2014. Public Health England. https://www.gov.uk/government/statistics/sexually-transmitted-infections-stis-annual-data-tables (accessed 29 Mar2016).

2 Horner P, Blee K, O'Mahony C, *et al.* 2015 UK National Guideline on the management of non-gonococcal urethritis. *Int J STD AIDS* 2016;**27**:85–96. doi:10.1177/0956462415586675

3 Pond MJ, Nori A V, Patel S, *et al.* Performance evaluation of automated urine microscopy as a rapid, non-invasive approach for the diagnosis of non-gonococcal urethritis. *Sex Transm Infect* 2015;**91**:165–70. doi:10.1136/sextrans-2014-051761

For peer review only - http://bmjopen.bmj.com/site/about/guidelines.xhtml

4 Smith R, Copas AJ, Prince M, *et al.* Poor sensitivity and consistency of microscopy in the diagnosis of low grade non-gonococcal urethritis. *Sex Transm Infect* 2003;**79**:487–90. doi:10.1136/sti.79.6.487

5 Menon-Johansson AS, McNaught F, Mandalia S, *et al.* Texting decreases the time to treatment for genital Chlamydia trachomatis infection. *Sex Transm Infect* 2006;**82**:49–51. doi:10.1136/sti.2004.014381

6 Bowie WR, Alexander ER, Stimson JB, *et al.* Therapy for nongonococcal urethritis: double-blind randomized comparison of two doses and two durations of minocycline. *Ann Intern Med* 1981;**95**:306–11.http://www.ncbi.nlm.nih.gov/pubmed/7271091 (accessed 24 Mar2016).

7 Horner P, Thomas B, Gilroy CB, *et al.* Role of Mycoplasma genitalium and Ureaplasma urealyticum in acute and chronic nongonococcal urethritis. *Clin Infect Dis* 2001;**32**:995–1003. doi:10.1086/319594

8 Darroch J, Myers L, Cassell J. Sex differences in the experience of testing positive for genital chlamydia infection: a qualitative study with implications for public health and for a national screening programme. *Sex Transm Infect* 2003;**79**:372–3.http://www.ncbi.nlm.nih.gov/pubmed/14573831 (accessed 10 Mar2016).

9 Kangas I, Andersen B, Olesen F, *et al.* Psychosocial impact of Chlamydia trachomatis testing in general practice. *Br J Gen Pract* 2006;**56**:587–93.http://www.ncbi.nlm.nih.gov/pubmed/16882376 (accessed 10 Mar2016).

10 Holgate HS, Longman C. Some peoples' psychological experiences of attending a sexual health clinic and having a sexually transmitted infection. *J R Soc Health* 1998;**118**:94–6.http://www.ncbi.nlm.nih.gov/pubmed/10076643 (accessed 10 Mar2016).

11 Morris JL, Lippman SA, Philip S, *et al.* Sexually transmitted infection related stigma and shame among African American male youth: implications for testing practices, partner notification, and treatment. *AIDS Patient Care STDS* 2014;**28**:499–506. doi:10.1089/apc.2013.0316

12 Mills N, Daker-White G, Graham A, *et al.* Population screening for Chlamydia trachomatis infection in the UK: a qualitative study of the experiences of those screened. *Fam Pract* 2006;**23**:550–7. doi:10.1093/fampra/cml031

13 Gottlieb SL, Stoner BP, Zaidi AA, *et al.* A prospective study of the psychosocial impact of a positive Chlamydia trachomatis laboratory test. *Sex Transm Dis* 2011;**38**:1004–11. doi:10.1097/OLQ.0b013e31822b0bed

14 Kangas I, Andersen B, Olesen F, *et al.* Psychosocial impact of Chlamydia trachomatis testing in general practice. *Br J Gen Pract* 2006;**56**:587–93.http://www.ncbi.nlm.nih.gov/pubmed/16882376 (accessed 27 Sep2016).

15 Robertson S, Williamson P. Men and health promotion in the UK: Ten years further on? *Health Educ J* 2005;**64**:293–301. doi:10.1177/001789690506400402

16 Cameron E, Bernardes J. Gender and disadvantage in health: men's health for a change. *Sociol Heal & Illn* 1998;**20**:673–93. doi:10.1111/1467-9566.00124

17 Connell R. *Masculinities*. University of California Press 2005. http://www.ucpress.edu/book.php?isbn=9780520246980 (accessed 28 Mar2017).

18 Hoy S. Beyond Men Behaving Badly: A Meta-Ethnography of Men's Perspectives on Psychological Distress and Help Seeking. *Int J Mens Health* 2012;**11**:202–6. doi:10.3149/jmh.1103.202

19 Collumbien M, Hawkes S. Missing men's messages: Does the reproductive health approach respond to men's sexual health needs? *Cult Health Sex* 2000;**2**:135–50. doi:10.1080/136910500300769

20 Banks I. No man's land: men, illness, and the NHS. *BMJ* 2001;**323**.http://www.bmj.com/content/323/7320/1058.long (accessed 31 Mar2017).

21 Rosaleen O, Hunt K, Hart G, *et al. Social science & medicine.* Elsevier Science 2005. http://econpapers.repec.org/article/eeesocmed/v_3a61_3ay_3a2005_3ai_3a3_3ap_3a503-516.htm (accessed 28 Mar2017).

22 Sonnenberg P, Clifton S, Beddows S, *et al.* Prevalence, risk factors, and uptake of interventions for sexually transmitted infections in Britain: findings from the National Surveys of Sexual Attitudes and Lifestyles (Natsal). *Lancet (London, England)* 2013;**382**:1795–806. doi:10.1016/S0140-6736(13)61947-9

23 Herdman M, Gudex C, Lloyd A, *et al.* Development and preliminary testing of the new five-level version of EQ-5D (EQ-5D-5L). *Qual Life Res* 2011;**20**:1727–36. doi:10.1007/s11136-011-9903-x

24 Petersen W. Society and the Adolescent Self-Image. Morris Rosenberg. Princeton University Press, Princeton, N.J., 1965. xii + 326 pp. $6.50. *Science (80-)* 1965;**148**:804–804. doi:10.1126/science.148.3671.804

25 Zimet GD, Dahlem NW, Zimet SG, *et al.* The Multidimensional Scale of Perceived Social Support. *J Pers Assess* 1988;**52**:30–41. doi:10.1207/s15327752jpa5201_2

For peer review only - http://bmjopen.bmj.com/site/about/guidelines.xhtml

26 Zimet G. *Multidimensional Scale of Perceived Social Support (MSPSS) - Scale Items and Scoring Information*. 2016.
27 Dolan P. Modeling valuations for EuroQol health states. *Med Care* 1997;**35**:1095–108.http://www.ncbi.nlm.nih.gov/pubmed/9366889 (accessed 19 Dec2017).
28 NICE. National Institute of Clinical Excellence Guide to the methods of technology appraisal 2013. Published Online First: 2013.https://www.nice.org.uk/guidance/pmg9/resources/guide-to-the-methods-of-technology-appraisal-2013-pdf-2007975843781 (accessed 2 Jan2018).
29 Campbell R, Mills N, Sanford E, *et al.* Does population screening for Chlamydia trachomatis raise anxiety among those tested? Findings from a population based chlamydia screening study. *BMC Public Health* 2006;**6**:106. doi:10.1186/1471-2458-6-106
30 Shoveller JA, Knight R, Johnson J, *et al.* 'Not the swab!' Young men's experiences with STI testing. *Sociol Health Illn* 2010;**32**:57–73. doi:10.1111/j.1467-9566.2009.01222.x
31 Mulholland E, Van Wersch A. Stigma, sexually transmitted infections and attendance at the GUM Clinic: an exploratory study with implications for the theory of planned behaviour. *J Health Psychol* 2007;**12**:17–31. doi:10.1177/1359105306069098
32 Lindberg C, Lewis-Spruill C, Crownover R. Barriers to sexual and reproductive health care: urban male adolescents speak out. *Issues Compr Pediatr Nurs* 2006;**29**:73–88. doi:10.1080/01460860600677577
33 Jackson LJ, Roberts TE. Measuring Health and Quality of Life for Women Undergoing Testing and Screening for Chlamydia: A Systematic Review. *Sex Transm Dis* 2016;**43**:152–64. doi:10.1097/OLQ.0000000000000407
34 Doward LC, McKenna SP, Meads DM, *et al.* The development of the Herpes Symptom Checklist and the Herpes Outbreak Impact Questionnaire. *Value Health*;**12**:139–45. doi:10.1111/j.1524-4733.2008.00424.x
35 Badia X, Colombo JA, Lara N, *et al.* Combination of qualitative and quantitative methods for developing a new Health Related Quality of Life measure for patients with anogenital warts. *Health Qual Life Outcomes* 2005;**3**:24. doi:10.1186/1477-7525-3-24
36 Mast TC, Zhu X, Demuro-Mercon C, *et al.* Development and psychometric properties of the HPV Impact Profile (HIP) to assess the psychosocial burden of HPV. *Curr Med Res Opin* 2009;**25**:2609–19. doi:10.1185/03007990903238786
37 Dominiak-Felden G, Cohet C, Atrux-Tallau S, *et al.* Impact of human papillomavirus-related genital diseases on quality of life and psychosocial wellbeing: results of an observational, health-related quality of life study in the UK. *BMC Public Health* 2013;**13**:1065. doi:10.1186/1471-2458-13-1065
38 Drolet M, Brisson M, Maunsell E, *et al.* The impact of anogenital warts on health-related quality of life: a 6-month prospective study. *Sex Transm Dis* 2011;**38**:949–56. doi:10.1097/OLQ.0b013e3182215512
39 Vriend HJ, Nieuwkerk PT, van der Sande MAB. Impact of genital warts on emotional and sexual well-being differs by gender. *Int J STD AIDS* 2014;**25**:949–55. doi:10.1177/0956462414526706
40 Woodhall S, Ramsey T, Cai C, *et al.* Estimation of the impact of genital warts on health-related quality of life. *Sex Transm Infect* 2008;**84**:161–6. doi:10.1136/sti.2007.029512
41 Miyai T, Turner KR, Kent CK, *et al.* The psychosocial impact of testing individuals with no history of genital herpes for herpes simplex virus type 2. *Sex Transm Dis* 2004;**31**:517–21.http://www.ncbi.nlm.nih.gov/pubmed/15480111 (accessed 6 Dec2016).
42 Jones SG, Pat Patsdaughter CA, Martinez Cardenas VM. Lessons from the viagra study: methodological challenges in recruitment of older and minority heterosexual men for research on sexual practices and risk behaviors. *J Assoc Nurses AIDS Care* 2011;**22**:320–9. doi:10.1016/j.jana.2010.10.009
43 Lorimer K, Reid ME, Hart GJ. 'It has to speak to people's everyday life...'; qualitative study of men and women's willingness to participate in a non-medical approach to Chlamydia trachomatis screening. *Sex Transm Infect* 2009;**85**:201–5. doi:10.1136/sti.2008.031138
44 Burkill S, Copas A, Couper MP, *et al.* Using the Web to Collect Data on Sensitive Behaviours: A Study Looking at Mode Effects on the British National Survey of Sexual Attitudes and Lifestyles. *PLoS One* 2016;**11**:e0147983. doi:10.1371/journal.pone.0147983
45 National Research Council. *Nonresponse in Social Science Surveys*. Washington, D.C.: : National Academies Press 2013. doi:10.17226/18293
46 Marsden P V., Wright JD. *Handbook of survey research*. Emerald 2010. https://books.google.co.uk/books/about/Handbook_of_Survey_Research.html?id=mMPDPXpTP-0C (accessed 28 Mar2017).
47 Low N, Sterne JA, Barlow D. Inequalities in rates of gonorrhoea and chlamydia between black ethnic groups in south east London: cross sectional study. *Sex Transm Infect* 2001;**77**:15–

For peer review only - http://bmjopen.bmj.com/site/about/guidelines.xhtml

20.http://www.ncbi.nlm.nih.gov/pubmed/11158686 (accessed 28 Mar2017).
48 Le Polain De Waroux O, Harris RJ, Hughes G, *et al.* The epidemiology of gonorrhoea in London: a Bayesian spatial modelling approach. *Epidemiol Infect* 2013;**142**:1–10. doi:10.1017/S0950268813000745
49 Murray BL. Qualitative research interviews: therapeutic benefits for the participants. *J Psychiatr Ment Health Nurs* 2003;**10**:233–6.http://www.ncbi.nlm.nih.gov/pubmed/12662341 (accessed 28 Mar2017).
50 Hutchinson SA, Wilson ME, Wilson HS. Benefits of Participating in Research Interviews. *Image J Nurs Scholarsh* 1994;**26**:161–6. doi:10.1111/j.1547-5069.1994.tb00937.x
51 Fenton KA, Johnson AM, McManus S, *et al.* Measuring sexual behaviour: methodological challenges in survey research. *Sex Transm Infect* 2001;**77**:84–92.http://www.ncbi.nlm.nih.gov/pubmed/11287683 (accessed 24 Mar2016).

For peer review only

For peer review only - http://bmjopen.bmj.com/site/about/guidelines.xhtml

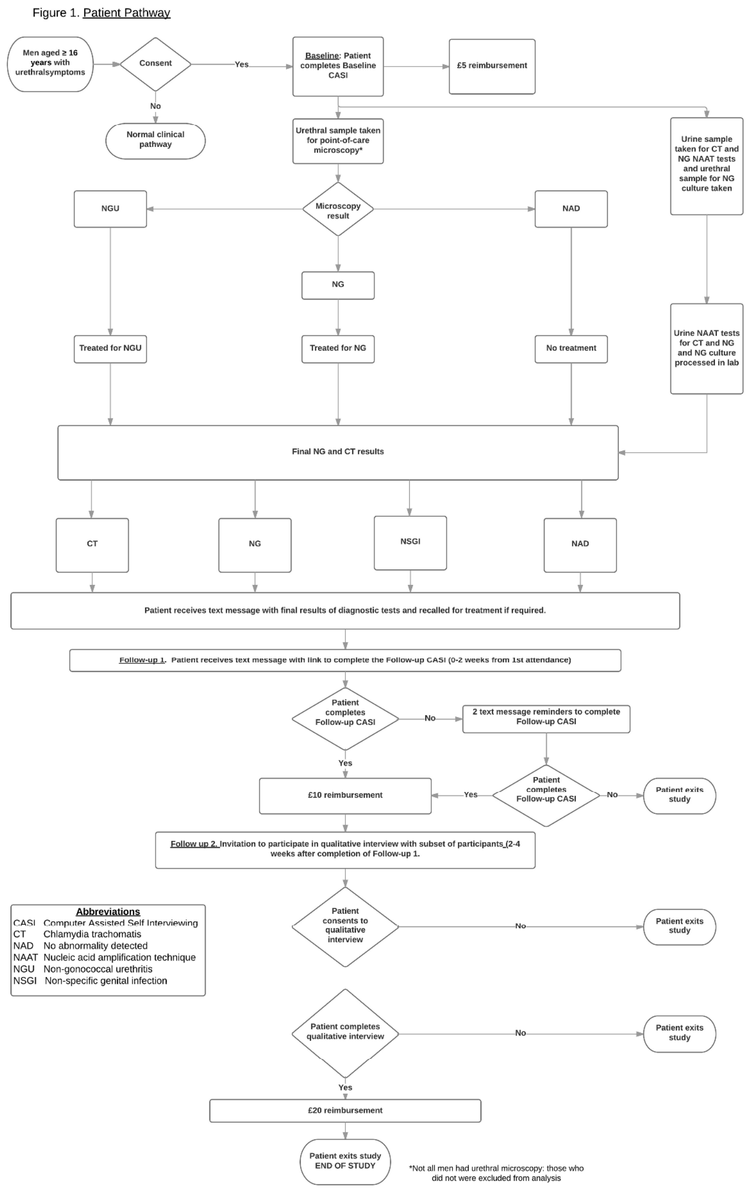

Figure 1. Patient Pathway

279x421mm (300 x 300 DPI)

Figure 2. Enrolment and Follow-up Chart

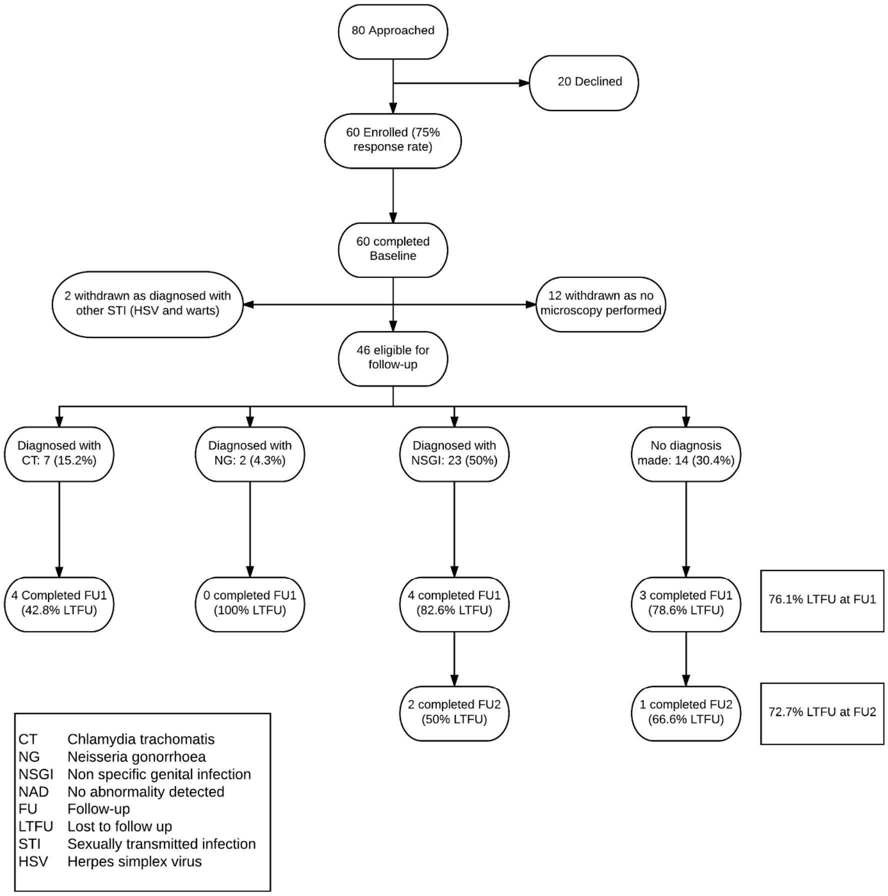

Figure 2. Enrolment and Follow-up Flow-chart

215x279mm (300 x 300 DPI)

Supplementary Appendix 1.

Baseline CASI Questionnaire

The following questions are about your background

1. Are you in full time employment? Yes/No/ Prefer not to answer

2. How old were you when you left full-time education?
   - ☐ 16 years or less
   - ☐ 17 or 18 years
   - ☐ 19 years or over
   - ☐ Still in full-time education

3. What is your highest educational achievement?
   - ☐ No qualifications / No formal qualifications
   - ☐ 1-4 GCSEs or equivalent qualifications
   - ☐ 5 GCSEs or equivalent qualifications.
   - ☐ Apprenticeships
   - ☐ 2 or more A-levels or equivalent qualifications.
   - ☐ Bachelors degree or equivalent, and higher qualifications.
   - ☐ Other qualifications including foreign qualifications.

5. Do you have any of the following long-standing conditions? (Tick ALL that apply)
   - ☐ Deafness or severe hearing impairment
   - ☐ Blindness or partially sighted
   - ☐ A long-standing physical condition
   - ☐ A learning disability
   - ☐ A mental health condition
   - ☐ A long-standing illness, such as cancer, HIV, diabetes, chronic heart disease, or epilepsy

6. Does this condition(s) cause you difficulty with any of the following? (Tick ALL that apply)
   - ☐ Everyday activities that people your age can usually do
   - ☐ At work, in education, or training
   - ☐ Access to buildings, streets or vehicles
   - ☐ Reading or writing
   - ☐ People's attitudes to you because of your condition
   - ☐ Communicating, mixing with others, or socialising
   - ☐ Any other activity
   - ☐ No difficulty with any of these

7. To which of these ethnic groups would you say you belong? (Tick ONE only)

a. WHITE
   - ☐ English/Welsh/Scottish/Northern Irish/British
   - ☐ Irish
   - ☐ Gypsy or Irish Traveller

For peer review only - http://bmjopen.bmj.com/site/about/guidelines.xhtml

- ☐ Any other White background, write in...

b. MIXED / MULTIPLE ETHNIC GROUPS

- ☐ White and Black Caribbean
- ☐ White and Black African
- ☐ White and Asian
- ☐ Any other Mixed/multiple ethnic background, write in...

c. ASIAN / ASIAN BRITISH

- ☐ Indian
- ☐ Pakistani
- ☐ Bangladeshi
- ☐ Chinese
- ☐ Any other Asian background, write in...

d. BLACK / AFRICAN / CARIBBEAN / BLACK BRITISH

- ☐ African
- ☐ Caribbean
- ☐ Any other Black / African / Caribbean background, write in...

e. OTHER ETHNIC GROUP

- ☐ Arab
- ☐ Any other ethnic group, write in...

The following are questions about recent infections

8. Have you ever been diagnosed with a sexually transmitted infection? If so which?
TICK ALL THAT APPLY

- ☐ Chlamydia
- ☐ Gonorrhoea
- ☐ Genital Herpes
- ☐ Non Specific Genital Infection (NSGI)
- ☐ Genital Warts
- ☐ Syphilis
- ☐ HIV
- ☐ Other (write in)
- ☐ Prefer not to answer

The following questions are about relationships and break-ups

9. At present are you...

- ☐ Married and living with wife
- ☐ Cohabiting but not married
- ☐ Separated, divorced, or, widowed
- ☐ Single (that is never married or never registered in a same-sex civil partnership)
- ☐ Prefer not to answer

9a. How long have you been in this relationship for?

- ☐ 0 – 6 months

For peer review only

- ☐ More than 6 months but less than a year
- ☐ 1 – 2 years
- ☐ 2 or more years
- ☐ Prefer not to answer

10. In the last 30 days, have you broken up with someone you considered yourself to be in a relationship with (for example, your girlfriend or wife)?
    - ☐ Yes
    - ☐ No
    - ☐ Prefer not to answer

On the following screens please tap the statement which best describes your own health state today.

11. MOBILITY
    - ☐ I have no problems in walking about
    - ☐ I have slight problems in walking about
    - ☐ I have moderate problems in walking about
    - ☐ I have severe problems in walking about
    - ☐ I am unable to walk about

12. SELF-CARE
    - ☐ I have no problems washing or dressing myself
    - ☐ I have slight problems washing or dressing myself
    - ☐ I have moderate problems washing or dressing myself
    - ☐ I have severe problems washing or dressing myself
    - ☐ I am unable to wash or dress myself

13. USUAL ACTIVITIES (e.g. work, Study, housework, family or leisure activities)
    - ☐ I have no problems doing my usual activities
    - ☐ I have slight problems doing my usual activities
    - ☐ I have moderate problems doing my usual activities
    - ☐ I have severe problems doing my usual activities
    - ☐ I am unable to do my usual activities

14. PAIN / DISCOMFORT
    - ☐ I have no pain or discomfort
    - ☐ I have slight pain or discomfort
    - ☐ I have moderate pain or discomfort
    - ☐ I have severe pain or discomfort
    - ☐ I have extreme pain or discomfort

15. ANXIETY / DEPRESSION
    - ☐ I am not anxious or depressed
    - ☐ I am slightly anxious or depressed

For peer review only

☐ I am moderately anxious or depressed
☐ I am severely anxious or depressed
☐ I am extremely anxious or depressed

We would like to know how good or bad your health is TODAY.

This scale is numbered from 0 to 100.
100 means the best health you can imagine. 0 means the worst health you can imagine.

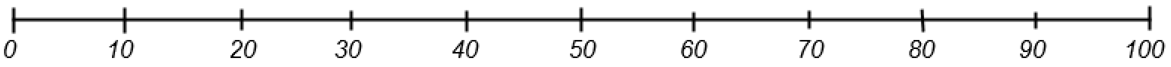

16. Please write the number you think best describes how you feel today. ______________

Below is a list of statements dealing with your general feelings about yourself. Please indicate how strongly you agree or disagree with each statement.

Strongly Agree   Agree   Disagree   Strongly Disagree

17. On the whole, I am satisfied with myself.
18. At times I think I am no good at all.
19. I feel that I have a number of good qualities.
20. I am able to do things as well as most other people.
21. I feel I do not have much to be proud of.
22. I certainly feel useless at times.
23. I feel that I'm a person of worth, at least on an equal plane with others.
24. I wish I could have more respect for myself.
25. All in all, I am inclined to feel that I am a failure.
26. I take a positive attitude toward myself.

Below is a list of statements dealing with your general feelings about your support from people around you. Please indicate how strongly you agree or disagree with each statement.
1 Very Strongly Disagree, 2 Strongly Disagree, 3 Mildly Disagree, 4 Neutral, 5 Mildly Agree, 6 Strongly Agree, 7 Very strongly agree

27. There is a special person who is around when I am in need
28. There is a special person with whom I can share my joys and sorrows
29. My family really tries to help me
30. I get all the emotional help and support I need from my family
31. I have a special person who is a real source of comfort to me
32. My friends really try to help me

For peer review only

33. I can count on my friends when things go wrong
34. I can talk about my problems with my family
35. I have friends with whom I can share my joys and sorrows
36. There is a special person in my life who cares about my feelings
37. My family is willing to help me make decisions.
38. I can talk about my problems with my friends.

We would now like to ask you if you have any feedback for us on how you found completing this questionnaire

39. How did you feel about completing this series of questions? Please write any comments in the space below.

____

____

____

Thank-you for completing this questionnaire
If you would like to speak to someone about the issues raised in this questionnaire please contact us.

For peer review only

Supplementary Appendix 2.

Follow-up 1 CASI Questionnaire

On the following screens please tap the statement which best describes your own health state today.

1. MOBILITY
- ☐ I have no problems in walking about
- ☐ I have slight problems in walking about
- ☐ I have moderate problems in walking about
- ☐ I have severe problems in walking about
- ☐ I am unable to walk about

2. SELF-CARE
- ☐ I have no problems washing or dressing myself
- ☐ I have slight problems washing or dressing myself
- ☐ I have moderate problems washing or dressing myself
- ☐ I have severe problems washing or dressing myself
- ☐ I am unable to wash or dress myself

3. USUAL ACTIVITIES (e.g. work, Study, housework, family or leisure activities)
- ☐ I have no problems doing my usual activities
- ☐ I have slight problems doing my usual activities
- ☐ I have moderate problems doing my usual activities
- ☐ I have severe problems doing my usual activities
- ☐ I am unable to do my usual activities

4. PAIN / DISCOMFORT
- ☐ I have no pain or discomfort
- ☐ I have slight pain or discomfort
- ☐ I have moderate pain or discomfort
- ☐ I have severe pain or discomfort
- ☐ I have extreme pain or discomfort

5. ANXIETY / DEPRESSION
- ☐ I am not anxious or depressed
- ☐ I am slightly anxious or depressed
- ☐ I am moderately anxious or depressed
- ☐ I am severely anxious or depressed
- ☐ I am extremely anxious or depressed

We would like to know how good or bad your health is TODAY.

This scale is numbered from 0 to 100.

For peer review only

100 means the best health you can imagine. 0 means the worst health you can imagine.

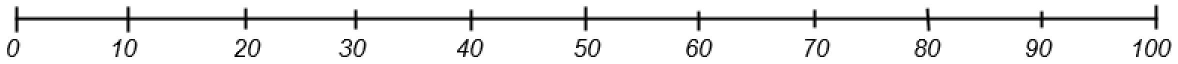

6. Please write the number you think best describes how you feel today. _______________

7. After you had received all your test results from your most recent visit to the GUM clinic were you diagnosed with any of the following infections: Chlamydia, Gonorrhoea or Non-specific urethritis (NSU)? ?
   - ☐ Yes
   - ☐ No
   - ☐ Not sure

The following questions are about your reaction after you were told your test was positive

1= Strongly Disagree 2 = Disagree 3 = Neutral 4 = Agree 5 = Strongly Agree

<u>After I was told that my test was positive:</u>

8. I talked to my friend(s) about it.	1	2	3	4	5
9. I do not think it was a big deal.	1	2	3	4	5
10. I was/am scared to tell my partner(s).	1	2	3	4	5
11. It does not change the way I feel about myself.	1	2	3	4	5
12. My partner thinks that I cheated on him or her.	1	2	3	4	5
13. I am not worried since it is curable.	1	2	3	4	5
14. I feel like I did not take care of myself.	1	2	3	4	5
15. I feel betrayed by my partner.	1	2	3	4	5
16. I worry that it will not go away.	1	2	3	4	5
17. I feel guilty that I could have given someone my infection	1	2	3	4	5
18. It has not changed my relationship with my partner.	1	2	3	4	5
19. I am not very proud of my actions.	1	2	3	4	5
20. I do not blame myself.	1	2	3	4	5
21. I think that my partner cheated on me.	1	2	3	4	5
22. I worry that I might not be able to have children in the future.	1	2	3	4	5
23. I do not really care that much.	1	2	3	4	5
24. I am angry.	1	2	3	4	5
25. I feel really bad about myself.	1	2	3	4	5
26. It does not change how my friends feel about me.	1	2	3	4	5
27. It will be hard for me to trust future partners.	1	2	3	4	5
28. I am not worried about getting it again.	1	2	3	4	5
29. I feel dirty.	1	2	3	4	5
30. I broke up with my partner.	1	2	3	4	5

31. I worry about what other sexually transmitted infections I could have been exposed to.	1	2	3	4	5
32. I feel really embarrassed.	1	2	3	4	5

The next questions are about how you feel generally about the infection you were diagnosed with

33. I think about the infection I was diagnosed with a lot.
1= Strongly Disagree 2 = Disagree 3 = Neutral 4 = Agree 5 = Strongly Agree

34. I am concerned about the infection I was diagnosed with.
1= Strongly Disagree 2 = Disagree 3 = Neutral 4 = Agree 5 = Strongly Agree

Below is a list of statements dealing with your general feelings about yourself. Please indicate how strongly you agree or disagree with each statement.

Strongly Agree Agree Disagree Strongly Disagree

35. On the whole, I am satisfied with myself.
36. At times I think I am no good at all.
37. I feel that I have a number of good qualities.
38. I am able to do things as well as most other people.
39. I feel I do not have much to be proud of.
40. I certainly feel useless at times.
41. I feel that I'm a person of worth, at least on an equal plane with others.
42. I wish I could have more respect for myself.
43. All in all, I am inclined to feel that I am a failure.
44. I take a positive attitude toward myself.

The following questions are about relationships and break-ups

45. At present are you...
- ☐ married and living with wife
- ☐ cohabiting but not married
- ☐ separated, divorced, or, widowed
- ☐ single (that is never married)
- ☐ prefer not to answer

45a. How long have you been in this relationship for?
- ☐ 0 – 6 months
- ☐ More than 6 months but less than a year
- ☐ 1 – 2 years
- ☐ 2 or more years
- ☐ Prefer not to answer

For peer review only

46. In the last 30 days, have you broken up with someone you considered yourself to be in a relationship with (for example, your girlfriend or wife)?

- ☐ Yes
- ☐ No
- ☐ Prefer not to answer

The next questions are about your most recent visit to the GUM Clinic

47. Have you ever visited this GUM Clinic before for the same condition?

- ☐ Yes
- ☐ No
- ☐ Prefer not to answer

48. Did a member of staff explain the results of the tests in a way you could understand?

- ☐ Yes definitely
- ☐ Yes, to some extent
- ☐ No
- ☐ Not sure, can't remember
- ☐ I was told I would get the results at a later date
- ☐ I was never told the results of the tests

49. While you were in the GUM Clinic how much information about your condition or treatment was given to you?

- ☐ Not enough
- ☐ Right amount
- ☐ Too much
- ☐ I was not given any information about my treatment or condition

50. Was the main reason you went to the GUM Clinic dealt with to your satisfaction?

- ☐ Yes, completely
- ☐ Yes, to some extent
- ☐ No

The next questions are about the symptoms you had when attending the clinic (the time you were first asked to join this study)

51. Please choose the option that best fits how you feel

- ☐ My symptoms have completely gone away
- ☐ My symptoms are better but not completely
- ☐ My symptoms are the same
- ☐ My symptoms are worse than they were before
- ☐ Don’t know/not sure
- ☐ Prefer not to answer

For peer review only

We would now like to ask you if you have any feedback for us on how you found completing this questionnaire

52. How did you feel about completing this series of questions? Please write any comments in the space below.

____
____
____

Thank-you for completing this questionnaire
If you would like to speak to someone about the issues raised in this questionnaire please contact us.

For peer review only

Supplementary Appendix 3.

Individual in-depth interview Topic Guide

Topics

Life history / framing (~5 minutes)

Please tell me a bit about yourself.

What is the most important thing you can tell me about yourself?

Probes:

Hobbies/interests

Goals

Tell me about your family.

Who do you include in your family? Partner/spouse? Father/mother? Grandparents? Children?

What sorts of things do you do with your day? / Describe a typical day in your life.

Probes:

Are you in school/university?

What do you do for money?

Clinical experience (~25 minutes)

Please describe how you were feeling while you were at the clinic on the day you were asked to take part in this study.

Probes:

Describe the reasons why you came to clinic

(If symptoms) What affect (if any) did your symptoms have on your day-to-day life?

How did it go/ how did you feel about the visit?

How long were you waiting to be seen? Was this expected /unexpected?

Which infections were you tested for?

What did the doctor tell you they thought you had? (i.e. diagnosis, if any)

Now let's discuss your diagnosis.

What thoughts were going through your mind when you were told you had NSU/Ng/possibly an STI (use patients' own description/language for their diagnosis)?

Probes:

How did you feel about your diagnosis?

For peer review only

Have you been diagnosed with NSU/Ng before? Any other infections? If yes, please describe any differences in your feelings from the first time you were diagnosed compared with this recent diagnosis.

Did you tell anyone (partner/family members/friends) your diagnosis? If yes, who and why? What was their reaction?

Research experience (~15 minutes)

We want to make sure that the research we conduct is done in the best way possible for patients. This next section of our interview is based on your experience with this research study so far. Please remember that your answers to these questions will not affect your clinical care, your legal rights, or future invitations to participate in research. It is important that you speak freely so that we can improve future research in this area.

How did you feel about your participation in the research?

Probes:

Would you consider involving yourself in this kind of research in the future? Why/why not?

Were you able to complete the surveys?

Did you have any problem accessing the CASI? (Logistical/technical problems) If so, what were the problems you faced?

Did you feel comfortable accessing the CASI (inside/outside of clinic)? Why/why not?

How did you feel about the questionnaires?

Discuss each questionnaire individually in turn: demographic and clinic experience questions; EQ5D-5L; social support scale; self-esteem scale:

Did you feel these questions were relevant?

Did you feel comfortable answering these questions? Why/why not?

Discuss any suggestions you may have for improvement of these questionnaires

Anything else?

Thanks

Reimbursement

For peer review only

Supplementary Appendix 4.

Full list of themes for Qualitative Interview (Follow-up 2)

List of nodes
antibiotics
attitudes to safe sex and sexual health
consultation
different SHCs
familiarity with SHC
feelings about attending SHC
feelings about being interviewed
feelings about participation in the research
feelings about positive diagnosis
future research
HIV vs. other STIs
interview
invasive tests
NSGI vs. CT
obstacles to participation in research
perception of CT
reaction to NSGI
reasons for participating in research
relationship
reasons for attending SHC
results
risk perception
social support
STI prevention
suggestions for research
survey - was it easy
survey - was it engaging
survey - was it relevant
survey comfort
treatment
waiting for results
waiting time in SHC

For peer review only

STROBE Statement—checklist of items that should be included in reports of observational studies

	Item No.	Recommendation	Page No.	Relevant text from manuscript
**Title and abstract**	1	(*a*) Indicate the study's design with a commonly used term in the title or the abstract	1-2	We conducted an observational study among symptomatic heterosexual men attending SHCs to evaluate the psychosocial impact of an NSGI diagnosis compared to a diagnosis of CT, NG or no abnormalities detected (NAD) focussing on the feasibility of our study methodology.
		(*b*) Provide in the abstract an informative and balanced summary of what was done and what was found	1-2	**ABSTRACT**<br>**Introduction:**<br>Non-specific genital infection (NSGI; non-*Chlamydia trachomatis* (CT), non-*Neisseria gonorrhoeae* (NG) associated urethritis) is a common diagnosis in symptomatic heterosexual men attending UK Sexual Health Clinics (SHCs) but little is known about the psychosocial impact of this diagnosis.<br>**Methods:**

For peer review only - http://bmjopen.bmj.com/site/about/guidelines.xhtml

We conducted an observational study among symptomatic heterosexual men attending SHCs to evaluate the psychosocial impact of an NSGI diagnosis compared to a diagnosis of CT, NG or no abnormalities detected (NAD) focussing on the feasibility of our study methodology. Participants completed a Computer Assisted Self Interview (CASI) including validated health-related quality-of-life (HRQoL) and self-esteem measures before diagnostic testing and two-weeks after receiving test results, and a qualitative interview. We compared scores between diagnostic groups using paired t-tests, qualitative data were analysed thematically, and feasibility was assessed by process analysis.

Results:

For peer review only - http://bmjopen.bmj.com/site/about/guidelines.xhtml

60 men completed the baseline CASI (75% response rate). 46 (76.6%) were eligible for follow-up; of these, 11 (23.9%) completed the follow-up CASI, and 3 (27.3%) completed the qualitative interview. 81.7% of participants left CASI feedback at baseline: 73.5% reported the questionnaire as ‘fine’ or ‘very good’. Qualitative interview participants reported the study was acceptable. All patients completing FU-1 showed measurable decreases from baseline to FU-1 in self-esteem (CT p=0.009; NSGI p=0.008; NAD p=0.057). Measurable decreases in HRQoL from baseline to FU-1 were found among participants with CT (p=0.002) or NSGI (p=0.001). We did not find differences in baseline sociodemographic factors between participants who completed FU-1 and those that did not.

For peer review only

				**Conclusions:** Although most participants indicated study acceptability at baseline, and we employed measures to increase retention (CASI questionnaires, reminder messages, a focus on men's health), attrition was high. We found that heterosexual men attending SHCs with symptoms of urethritis may experience negative psychosocial impacts regardless of their diagnosis, which warrants further investigation. We recommend exploring novel strategies to decrease attrition in future studies.
**Introduction**				
Background/rationale	2	Explain the scientific background and rationale for the investigation being reported	2-4	**INTRODUCTION** Non-specific Genital Infection (NSGI) or, non-*Chlamydia trachomatis* (CT), non-*Neisseria gonorrhoeae* (NG) associated urethritis (NGU), is the third

For peer review only - http://bmjopen.bmj.com/site/about/guidelines.xhtml

most frequently diagnosed condition in heterosexual men attending sexual health clinics (SHCs) in the United Kingdom (UK), with 30,381 cases/year compared to CT (42,483/year) and first presentation of genital warts (32,656/year) [1]. NSGI is a diagnosis of exclusion; men with urethral symptoms are screened with urethral microscopy in SHCs, and if this demonstrates urethritis with no gonococci, a provisional diagnosis of NGU is made [2]. If urinary Nucleic Acid Amplification Tests (NAATs) sent for NG and CT are both negative, the diagnosis is recorded as NSGI.

Although a common condition, NSGI remains poorly understood. NSGI can be caused by a variety of sexually transmitted infections (STIs), including *Mycoplasma*

For peer review only - http://bmjopen.bmj.com/site/about/guidelines.xhtml

genitalium (MG) and *Trichomonas vaginalis* (TV), which are not routinely tested for in SHCs, and many cases have an uncertain aetiology [2]. Microscopy, on which the diagnosis rests, has poor specificity for known causes of urethritis [3] and is subject to considerable observer variation [4].

National guidelines recommend that men diagnosed with NGU are informed they may have an STI, given same day empirical antibiotics, advised their partners should attend for STI testing and empirical treatment, and to abstain from sex until treatment is completed [2]. Men receive CT/NG NAAT results usually within 8 days [5]. Following first-line antibiotics for NSGI, 10%–20% of men return because of persistent symptoms [6,7].

For peer review only

For peer review only - http://bmjopen.bmj.com/site/about/guidelines.xhtml

Men who undergo STI testing, and are diagnosed with an STI report negative impacts on their psychosocial wellbeing, including feelings of stigma [8–10], however, to our knowledge, there are no published data on the psychosocial impact of NSGI diagnosis among men. Stigma has been shown in men to be associated with a delay in testing for STIs, and decreased willingness to notify their casual partners of their STI diagnosis [11]. Studies on psychosocial impact, partner notification and quality of life impact have predominantly been carried out in women [9,12–14], with few studies recruiting men. A greater understanding of the degree and nature of psychosocial impacts of a diagnosis of an STI, or a 'presumed STI' such as NSGI, in men, is important to optimise

For peer review only - http://bmjopen.bmj.com/site/about/guidelines.xhtml

interventions, including partner notification and risk-behaviour modification, which aim to reduce the negative impact of STIs, such as complications of infection.

Men are reportedly poor users of health services, largely due to culturally dominant ideals of masculinity that equate illness with weakness, which may impede care-seeking [15–18]. In sexual healthcare, there has traditionally been a focus on female reproductive health [19], and this may further discourage male attendance [19,20]. However, men are willing to respond openly to questions about their sexual health, particularly when enquiries are focused specifically on men's needs [19,21].

We decided to focus on heterosexual men in this study

For peer review only - http://bmjopen.bmj.com/site/about/guidelines.xhtml

as there are important differences between men who have sex with men (MSM) and men who have sex exclusively with women (MSEW) in their access of SHC services, reported sexual health risks, their own perception of risk of STIs, and sexual health and well being outcomes, suggesting that their experience of diagnosis of a genital infection may also be different. The Natsal-3 study reported that in comparison with MSEW, MSM are more likely to report higher numbers of partners, higher rates of condomless sex, to perceive themselves at higher rates of STIs, a greater likelihood to attend SHCs, and higher rates of depression, substance use and non-volitional sex [22]. Due to these important differences between MSM and MSEW, we decided that psychosocial impacts of an STI diagnosis in

For peer review only - http://bmjopen.bmj.com/site/about/guidelines.xhtml

				MSM merited studying separately.<br><br>Because of a lack of models to measure the psychosocial impact of an NSGI diagnosis among men, and the potential difficulty in recruiting and retaining this group in sexual health studies, we designed a small-scale longitudinal mixed-method feasibility study to inform on our research methods and better understand the psychosocial and health related quality of life (HRQoL) status of symptomatic heterosexual men before and after receiving a diagnosis of NSGI, CT, NG or nothing abnormal detected (NAD).
Objectives	3	State specific objectives, including any prespecified hypotheses	4	Because of a lack of models to measure the psychosocial impact of an NSGI diagnosis among men, and the potential difficulty in recruiting and retaining this group in sexual health studies

For peer review only

				health studies, we designed a small-scale longitudinal mixed-method feasibility study to inform on our research methods and better understand the psychosocial and health related quality of life (HRQoL) status of symptomatic heterosexual men before and after receiving a diagnosis of NSGI, CT, NG or nothing abnormal detected (NAD).
**Methods**				
Study design	4	Present key elements of study design early in the paper	4	We conducted a longitudinal study in symptomatic heterosexual men using patient completed questionnaires assessing HRQoL and psychosocial status before and after receiving STI test results, and individual qualitative interviews to evaluate experiences at the SHC and acceptability of study methods.
Setting	5	Describe the setting, locations, and relevant dates, including periods of recruitment, exposure, follow-up, and data collection	4-5	Eligibility criteria were: heterosexual men presenting with

For peer review only - http://bmjopen.bmj.com/site/about/guidelines.xhtml

urethral symptoms (dysuria, discharge or urethral discomfort), aged ≥16 years at either of two participating London SHCs between September - October 2016. We excluded men who were asymptomatic, not available for follow-up, and who were unable to understand English.

Following informed consent, participants were invited to complete a baseline computer assisted self-interview (CASI) assessing psychosocial and HRQoL status in clinic prior to consultation, a follow-up CASI after receipt of all test results 1-2 weeks after baseline (FU-1) and a qualitative interview 2-4 weeks after baseline (FU-2).

CASIs were optimised for web-enabled devices including smartphones. We offered participants the choice to complete CASIs using their own

For peer review only - http://bmjopen.bmj.com/site/about/guidelines.xhtml

				device or a study tablet-device and use of a private room in clinic. Study staff sent ≤2 text message reminders for each follow-up. Participants who did not complete FU-1 were considered ineligible for FU-2. Participants received gift voucher reimbursements: £5 (baseline), £10 (FU-1), £20 (FU-2).
Participants	6	(*a*) *Cohort study*—Give the eligibility criteria, and the sources and methods of selection of participants. Describe methods of follow-up<br>*Case-control study*—Give the eligibility criteria, and the sources and methods of case ascertainment and control selection. Give the rationale for the choice of cases and controls<br>*Cross-sectional study*—Give the eligibility criteria, and the sources and methods of selection of participants	4-5	Eligibility criteria were: heterosexual men presenting with urethral symptoms (dysuria, discharge or urethral discomfort), aged ≥16 years at either of two participating London SHCs between September - October 2016. We excluded men who were asymptomatic, not available for follow-up, and who were unable to understand English. Following informed consent, participants were invited to complete a baseline computer assisted self-interview (CASI)

For peer review only - http://bmjopen.bmj.com/site/about/guidelines.xhtml

				assessing psychosocial and HRQoL status in clinic prior to consultation, a follow-up CASI after receipt of all test results 1-2 weeks after baseline (FU-1) and a qualitative interview 2-4 weeks after baseline (FU-2).
		(*b*) *Cohort study*—For matched studies, give matching criteria and number of exposed and unexposed<br>*Case-control study*—For matched studies, give matching criteria and the number of controls per case		
Variables	7	Clearly define all outcomes, exposures, predictors, potential confounders, and effect modifiers. Give diagnostic criteria, if applicable	5	**Diagnostic Criteria**<br>We categorised participants into four diagnostic groups based on microscopy and NAAT results: NSGI: NGU diagnosis on urethral microscopy and CT/NG NAATs negative; NG: NG on urethral microscopy and/or positive NAAT; CT: positive NAAT; NAD: no urethritis on microscopy and CT/NG NAATs negative.<br><br>**Outcome measures**<br>*Primary outcome:* Qualitative and quantitative process data on

For peer review only - http://bmjopen.bmj.com/site/about/guidelines.xhtml

For peer review only

				recruitment and retention rates at baseline and follow-up to inform on feasibility. Evaluation of potential relevance of questions validated in other populations for men diagnosed with NSGI.<br><br>*Secondary outcomes:*<br>Percentage change from baseline to FU-1 in mean EQ-5D-5L and Rosenberg Self-Esteem Scale in men diagnosed with CT, NG, NSGI or NAD.
Data sources/ measurement	8*	For each variable of interest, give sources of data and details of methods of assessment (measurement). Describe comparability of assessment methods if there is more than one group	4-5	The participant pathway is detailed in Figure 1. After completion of the baseline CASI, any participants diagnosed with an STI other than CT, NG or NSGI, or who did not have both urethral microscopy and urinary NAATs, were withdrawn from the study. Once participants were notified of their NAAT CT/NG results by the SHC (phone call/ text message), the research team

For peer review only - http://bmjopen.bmj.com/site/about/guidelines.xhtml

texted participants an electronic link to the FU-1 CASI.

Questionnaire design

Baseline and follow-up CASIs included validated scales measuring psychosocial status and HRQoL and additional questions. The EQ-5D-5L scale [17] as a measure of HRQoL and the Rosenberg Self-Esteem scale [18] as a measure of potential stigmatising effects of screening for STIs, were used in both baseline and FU-1 CASIs.

In addition to the above, the baseline CASI included the Multidimensional Scale of Social Support [19] to assess coping resources and questions on sociodemographic and current relationship status, and the FU-1 CASI included a question on current relationship status and a panel of questions

For peer review only

				previously developed to assess impact of CT diagnosis in women, [20] which we modified for use in men by removing a pregnancy-related question. Participants were asked to indicate acceptability of the CASIs (free-text response) at baseline and FU-1. (Baseline and FU-1 questionnaires and FU-2 topic guide: Appendix 1, Supplementary Information.)
Bias	9	Describe any efforts to address potential sources of bias	5-6	**Data analysis** *Questionnaire data:* Scores for psychosocial and HRQoL scales were calculated according to published methods [24][23][25]. For all eligible men, mean and 95% Confidence Intervals of baseline scores for EQ-5D-5L, Rosenberg Self-Esteem and Multidimensional Social Support Scales were compared in men diagnosed with CT, NG, NSGI and NAD, using paired t-tests. To enable

For peer review only - http://bmjopen.bmj.com/site/about/guidelines.xhtml

us to compare the EQ-5D-5L score across the different diagnoses (CT, NG, NSGI and NAD), a mean score was calculated: we calculated mean values for each of the five dimensions of the EQ-5D-5L, and a final score for the EQ-5D-5L was created using the sum of the mean value for each dimension. We calculated the mean value rather than an index score for the EQ-5D-5L to allow comparison between the outcomes of EQ-5D-5L with the Rosenberg Self-Esteem and Multidimensional Social Support Scales.

To test for non-response bias, we compared sociodemographic data of those who completed both baseline and follow-up to those who were lost to follow-up (LTFU) using Pearson's Chi^2 tests and Kruskal-Wallis tests. Paired t-tests were used to

For peer review only

assess differences in mean score for EQ-5D-5L and Rosenberg Self-Esteem Scales between baseline and follow-up in men diagnosed with CT, NG, NSGI and NAD. We used the students t-test distribution to calculate p-values. Statistical significance was set at $p<0.05$. Statistical analysis was carried out using STATA version 13.1 (StataCorp, 2015).

Qualitative data: Audio recordings were transcribed and transcripts checked for accuracy against the recording. Cleaned transcripts were then imported into NVivo (Version 10, 2012) for analysis. Two researchers assigned themes to text independently, with discrepancies resolved by consensus before final themes reported. All names of participants presented here are pseudonyms.

For peer review only - http://bmjopen.bmj.com/site/about/guidelines.xhtml

Study size	10	Explain how the study size was arrived at	5	As this was a feasibility study with no precedent, no formal sample size could be calculated. We planned to approach 100 patients at baseline as a convenience target, and assuming approximately 50% refusal, we expected 50-60 patients to be enrolled and estimated ~50% attrition at each follow-up.

Continued on next page

For peer review only

Quantitative variables	11	Explain how quantitative variables were handled in the analyses. If applicable, describe which groupings were chosen and why	5-6	**Data analysis** *Questionnaire data:* Scores for psychosocial and HRQoL scales were calculated according to published methods [24][23][25]. For all eligible men, mean and 95% Confidence Intervals of baseline scores for EQ-5D-5L, Rosenberg Self-Esteem and Multidimensional Social Support Scales were compared in men diagnosed with CT, NG, NSGI and NAD, using paired t-tests. To enable us to compare the EQ-5D-5L score across the different diagnoses (CT, NG, NSGI and NAD), a mean score was calculated: we calculated mean values for each of the five dimensions of the EQ-5D-5L, and a final score for the EQ-5D-5L was created using the sum of the mean value for each dimension. We calculated the mean value rather than an index score for the EQ-5D-5L to allow comparison between the outcomes of EQ-5D-5L with the

For peer review only - http://bmjopen.bmj.com/site/about/guidelines.xhtml

				Rosenberg Self-Esteem and Multidimensional Social Support Scales.<br><br>To test for non-response bias, we compared sociodemographic data of those who completed both baseline and follow-up to those who were lost to follow-up (LTFU) using Pearson's Chi^2 tests and Kruskal-Wallis tests. Paired t-tests were used to assess differences in mean score for EQ-5D-5L and Rosenberg Self-Esteem Scales between baseline and follow-up in men diagnosed with CT, NG, NSGI and NAD. We used the students t-test distribution to calculate p-values. Statistical significance was set at $p<0.05$. Statistical analysis was carried out using STATA version 13.1 (StataCorp, 2015).
Statistical methods	12	(*a*) Describe all statistical methods, including those used to control for confounding	5-6	**Data analysis**<br>*Questionnaire data:*<br>Scores for psychosocial and

For peer review only

For peer review only - http://bmjopen.bmj.com/site/about/guidelines.xhtml

	HRQoL scales were calculated according to published methods [24][23][25]. For all eligible men, mean and 95% Confidence Intervals of baseline scores for EQ-5D-5L, Rosenberg Self-Esteem and Multidimensional Social Support Scales were compared in men diagnosed with CT, NG, NSGI and NAD, using paired t-tests. To enable us to compare the EQ-5D-5L score across the different diagnoses (CT, NG, NSGI and NAD), a mean score was calculated: we calculated mean values for each of the five dimensions of the EQ-5D-5L, and a final score for the EQ-5D-5L was created using the sum of the mean value for each dimension. We calculated the mean value rather than an index score for the EQ-5D-5L to allow comparison between the outcomes of EQ-5D-5L with the Rosenberg Self-Esteem and Multidimensional Social Support Scales.

For peer review only - http://bmjopen.bmj.com/site/about/guidelines.xhtml

			To test for non-response bias, we compared sociodemographic data of those who completed both baseline and follow-up to those who were lost to follow-up (LTFU) using Pearson's Chi^2 tests and Kruskal-Wallis tests. Paired t-tests were used to assess differences in mean score for EQ-5D-5L and Rosenberg Self-Esteem Scales between baseline and follow-up in men diagnosed with CT, NG, NSGI and NAD. We used the students t-test distribution to calculate p-values. Statistical significance was set at p<0.05. Statistical analysis was carried out using STATA version 13.1 (StataCorp, 2015).
	(*b*) Describe any methods used to examine subgroups and interactions		
	(*c*) Explain how missing data were addressed	6	Pearson's Chi^2 tests and Kruskal-Wallis test results for non-response bias were not statistically significant so we assumed data were missing at random.
	(*d*) *Cohort study*—If applicable, explain how loss to follow-up was addressed<br>*Case-control study*—If applicable, explain how matching of cases and controls was addressed		

For peer review only

		Cross-sectional study—If applicable, describe analytical methods taking account of sampling strategy		
		(*e*) Describe any sensitivity analyses		
**Results**				
Participants	13*	(a) Report numbers of individuals at each stage of study—eg numbers potentially eligible, examined for eligibility, confirmed eligible, included in the study, completing follow-up, and analysed	6	We approached 80 patients, of whom 60 (75%) enrolled and completed the baseline CASI. 46 (76.6%) were eligible for follow-up; of these, 11 (23.9%) completed the FU-1 CASI, and 3 of these (27.3%) completed FU-2 (Figure 2.)
		(b) Give reasons for non-participation at each stage		See Figure 2
		(c) Consider use of a flow diagram		See Figure 2
Descriptive data	14*	(a) Give characteristics of study participants (eg demographic, clinical, social) and information on exposures and potential confounders	6-7	Participants' sociodemographic factors at baseline are presented in Table 1. Most were between 25-35 years old (60.9%) and over half reported their ethnicity as non-White (52.2%). 80.4% indicated they were not in a relationship and many had previously been diagnosed with STIs (30.4% NSGI, 28.3% CT, 13.0% genital herpes or warts and 8.7% NG).
		(b) Indicate number of participants with missing data for each variable of interest		

For peer review only - http://bmjopen.bmj.com/site/about/guidelines.xhtml

		(c) *Cohort study*—Summarise follow-up time (eg, average and total amount)	
Outcome data	15*	*Cohort study*—Report numbers of outcome events or summary measures over time	
		Case-control study—Report numbers in each exposure category, or summary measures of exposure	
		Cross-sectional study—Report numbers of outcome events or summary measures	
Main results	16	(*a*) Give unadjusted estimates and, if applicable, confounder-adjusted estimates and their precision (eg, 95% confidence interval). Make clear which confounders were adjusted for and why they were included	See Table 2
		(*b*) Report category boundaries when continuous variables were categorized	
		(*c*) If relevant, consider translating estimates of relative risk into absolute risk for a meaningful time period	

Continued on next page

For peer review only

Other analyses	17	Report other analyses done—eg analyses of subgroups and interactions, and sensitivity analyses		
**Discussion**				
Key results	18	Summarise key results with reference to study objectives	8-11	In this small feasibility study we detected decreases in mean HRQoL and self-esteem scores in participants with a final diagnosis of CT or NSGI from the time of clinical presentation to approximately one week after receiving NAAT results. Although participants diagnosed with CT and NSGI showed greater negative impact in this time period, interestingly, participants who ultimately received a NAD diagnosis also experienced decreased self-esteem scores. Studies using qualitative methods and non-validated questionnaires have shown men diagnosed with CT experience negative psychosocial impacts [7, 8]. However, this is the first time that a negative impact of diagnosis of NSGI has been demonstrated to our knowledge.

For peer review only - http://bmjopen.bmj.com/site/about/guidelines.xhtml

				We experienced high LTFU that resulted in low numbers of respondents within diagnostic categories, which meant we were unable to adjust our analysis for common confounders, such as education and socioeconomic status, known to be associated with gonorrhoea [26,27] . This may be a source of bias in our results.<br>In interviews, participants with NSGI reported concerns regarding the uncertainty of the diagnosis, and fear of the impact of a possible STI diagnosis on their relationship. These findings suggest receipt of an STI or presumptive STI diagnosis in this population may have complex psychosocial impacts. These impacts may be multi-factorial and anxiety seems to be highly contextual and affected by relationship status and knowledge about STIs.
Limitations	19	Discuss limitations of the study, taking into account sources of potential bias or imprecision. Discuss both direction and magnitude of any potential bias	9-11	Despite these mechanisms, and

For peer review only - http://bmjopen.bmj.com/site/about/guidelines.xhtml

positive participant feedback on the baseline CASI and in our qualitative interviews, we experienced high LTFU. High LTFU resulted in low numbers of respondents within diagnostic categories, which meant we were unable to adjust our analysis for common confounders, such as education and socioeconomic status, known to be associated with gonorrhoea [32,33] . This may be a source of bias in our results. We did not find significant differences between groups in terms of non-response bias and other characteristics, however this is not surprising as the power to detect differences between groups is greatly affected by sample size and our study included a small number of participants. Recruiting larger numbers of participants in future studies will increase the statistical power to detect any important differences between groups.

A weakness of our study is that we

For peer review only

For peer review only - http://bmjopen.bmj.com/site/about/guidelines.xhtml

did not include asymptomatic men in our study, hence we cannot exclude the possibility that the negative psychosocial impacts observed with men diagnosed with CT or NSGI were not related to the confounding factors of being symptomatic or the attendance at an SHC (including undergoing genital examination, and a urethral swab). Few studies have examined the impact of an SHC clinic visit on psychosocial and/or self-esteem outcomes for men, but those that have been conducted did report negative impacts; studies among heterosexual men attending SHCs who are diagnosed with an STI reported stigma, anxiety, isolation, concerns regarding relationships, feeling 'contaminated' and fear of exposure, as well as reluctance to attend an SHC clinic for help [27,36]. A focus group of young male adolescents reported that accessing SHC services was stressful due to fear of stigma and a

For peer review only

For peer review only - http://bmjopen.bmj.com/site/about/guidelines.xhtml

loss of social status, shame, and embarrassment, a lack of privacy/confidentiality, and challenges in accessing and negotiating the healthcare system[36]. Shoveller et al reported that factors involved in STI testing, such as urethral swab testing, were seen by men as fearful, and as a process where men felt very vulnerable; most of these male participants reported feeling anxious waiting for potentially bad news in addition to feeling that their masculinity had been compromised through an admission of needing help [37]. Limitations of these studies are that they did not specify whether patients had symptoms, or if men had experienced (invasive or non-invasive) testing procedures, and tended to recruit patients who had already been diagnosed with an STI, which makes the impact of testing and receiving a diagnosis difficult to separate [10,27,36,37]. Men who used postal screening for

For peer review only - http://bmjopen.bmj.com/site/about/guidelines.xhtml

CT and had negative test results were not found to experience a negative impact on self-esteem or anxiety, suggesting that STI testing at home may reduce negative impacts associated with visiting an SHC, however this study did not follow-up men with positive CT diagnoses[12]. Future studies should consider including asymptomatic men alongside symptomatic men, to better evaluate the impact of attending clinic, and the experience of having genital examination and invasive testing or not, on psychosocial wellbeing and self-esteem.

The issue of gender concordance between participants and interviewers/researchers has been discussed in the literature [38] and the fact that our study recruiter was female may have affected recruitment and retention. A study investigating attitudes to Viagra in heterosexual Hispanic men, using telephone qualitative interviews,

For peer review only - http://bmjopen.bmj.com/site/about/guidelines.xhtml

found that changing from female to male recruiters and data collectors increased their recruitment [26]. Participants were offered the option of a male or female interviewer but may not have felt comfortable with asking for a particular interviewer, and so this may not have been sufficient to reduce the potential impact of gender discordance on acceptance of the invitation to an interview in our study. We suggest future studies involving qualitative interviews should consider the gender of recruiters and interviewers.

We received feedback from our participants that some of the wording of questions on our CASIs did not appear to be directly relevant to our study aims. Future studies should ensure that data collection measures use terms that are clear and familiar to participants, possibly through development of questionnaires with

For peer review only - http://bmjopen.bmj.com/site/about/guidelines.xhtml

focus groups. Some men in our study also saw participation as an opportunity to learn more about their own health and requested that future studies offer research participants material on health promotion, i.e. material on the infection they were diagnosed with and how to reduce risk of infection.

Although the Rosenberg Self Esteem scale and the EQ-5D-5L scales we used in this study have been validated in men and women, the panel of questions used to assess men's feelings about a diagnosis of CT or NSGI in the FU-1 CASI has not been validated in men, or for non-CT STI diagnoses. In addition, we assessed psychosocial and HRQoL impact at a single time-point, which does not measure durability of the observed decrease in self-esteem in this population, and data suggest impact of STI diagnoses may decrease over time [39].

For peer review only - http://bmjopen.bmj.com/site/about/guidelines.xhtml

Interpretation	20	Give a cautious overall interpretation of results considering objectives, limitations, multiplicity of analyses, results from similar studies, and other relevant evidence	12	In conclusion, our study shows that heterosexual men with urethral symptoms may experience negative psychosocial effects following SHC attendance. Given that incidence of NSGI is high [16] and up to 20% of men affected have recurrent symptoms [5, 6], a significant number of men may be adversely affected. Future research in this area is needed, firstly to address the high level of LTFU, and then to test the generalisability of our findings as to whether there are significant psychosocial harms of giving presumptive and non-specific diagnoses.
Generalisability	21	Discuss the generalisability (external validity) of the study results	12	Future research in this area is needed, firstly to address the high level of LTFU, and then to test the generalisability of our findings as to whether there are significant psychosocial harms of giving presumptive and non-specific diagnoses.

For peer review only

**Other information**				
Funding	22	Give the source of funding and the role of the funders for the present study and, if applicable, for the original study on which the present article is based	15	This study was funded by an Innovate UK (SBRI grant no. 971452) awarded to Atlas Genetics Ltd. Neither Atlas Genetics nor Innovate UK had a role in the design, collection, management, analysis, interpretation of data or decision to submit a report.

*Give information separately for cases and controls in case-control studies and, if applicable, for exposed and unexposed groups in cohort and cross-sectional studies.

Note: An Explanation and Elaboration article discusses each checklist item and gives methodological background and published examples of transparent reporting. The STROBE checklist is best used in conjunction with this article (freely available on the Web sites of PLoS Medicine at http://www.plosmedicine.org/, Annals of Internal Medicine at http://www.annals.org/, and Epidemiology at http://www.epidem.com/). Information on the STROBE Initiative is available at www.strobe-statement.org.

For peer review only

For peer review only - http://bmjopen.bmj.com/site/about/guidelines.xhtml
